# OTUB2-mediated deubiquitination upregulates U2AF2 to promote colorectal cancer evasion of autophagy-ferroptosis

**DOI:** 10.1038/s41419-026-08415-8

**Published:** 2026-05-07

**Authors:** Xi Chen, Yanxin Qi, Qigang Nie, Kai Zhou, Ao Mo

**Affiliations:** 1 Department of Gastric Surgery, The First Affiliated Hospital of University of Science and Technology of China, Hefei, Anhui China; 2 Division of Life Sciences and Medicine, University of Science and Technology of China, Hefei, Anhui China; 3 Day Surgery Ward, The First Affiliated Hospital of University of Science and Technology of China, Hefei, Anhui China; 4 Department of General Surgery, The First Affiliated Hospital of Army Medical University, Chongqing, China

**Keywords:** Apoptosis, Colon cancer

## Abstract

Colorectal cancer (CRC) is one of the leading causes of cancer-related mortality worldwide. Ferroptosis, an iron-dependent form of programmed cell death, has emerged as a potential therapeutic target. However, the regulatory mechanisms that allow CRC cells to evade ferroptosis are not fully understood. This study focuses on OTUB2, a deubiquitinating enzyme, and its role in stabilizing U2AF2, which allows CRC cells to resist ferroptosis and autophagy. We analyzed CRC cells and clinical samples to evaluate the effects of OTUB2 on U2AF2 deubiquitination. OTUB2 knockdown and overexpression models were established in CRC cell lines (LoVo, RKO, SW480, HT115) to assess ferroptosis and autophagy activity. Various assays, including western blotting, immunoprecipitation, colony formation, and transwell migration assays, were used to evaluate cell proliferation, migration, and iron metabolism markers. In vivo xenograft models were also employed to assess tumor growth under OTUB2-U2AF2 axis disruption. OTUB2 was highly expressed in CRC tissues compared to normal controls. Knockdown of OTUB2 significantly increased ferroptosis, while enhancing autophagy. Conversely, OTUB2 overexpression reduced ferroptosis and autophagy, maintaining CRC cell survival and proliferation. In vivo studies confirmed that disrupting the OTUB2-U2AF2 axis impaired tumor growth by activating both ferroptosis and autophagy. Importantly, a reciprocal activation relationship between ferroptosis and autophagy was observed under OTUB2-U2AF2 axis deficiency. OTUB2 stabilizes U2AF2 in CRC cells, enabling them to evade ferroptosis and autophagy. Disruption of the OTUB2-U2AF2 axis activates both processes, suppressing tumor growth. Targeting this axis presents a promising therapeutic strategy for CRC treatment.

## Introduction

Colorectal cancer (CRC) originates from the epithelial cells lining the colon or rectum. It typically begins as a small, non-cancerous polyp, which may eventually develop into cancer over time. Early-stage CRC often presents with few or no symptoms; however, as the disease progresses, patients may experience abdominal pain, rectal bleeding, and changes in bowel habits [1, 2]. Certain inherited syndromes, such as familial adenomatous polyposis (FAP) and Lynch syndrome, significantly increase the risk of developing CRC. Additionally, lifestyle factors like a high-fat and low-fiber diet, along with the consumption of red and processed meats, are associated with a higher CRC risk, whereas diets rich in fruits, vegetables, and whole grains are protective. Other risk factors include physical inactivity, obesity, smoking, and excessive alcohol consumption. Individuals with inflammatory bowel disease (IBD), such as Crohn’s disease and ulcerative colitis, are also at higher risk of developing CRC [3].

OTUB2 (Otubain 2), a deubiquitinating enzyme belonging to the Ubiquitin C-terminal hydrolase (UCH) family, plays a pivotal role in regulating protein ubiquitination, particularly in maintaining protein stability through deubiquitination [4]. OTUB2 has been implicated in cell cycle regulation, influencing cell proliferation and apoptosis [5], and it plays a key role in DNA damage repair by deubiquitinating critical repair proteins to maintain genomic stability [6, 7]. Abnormal OTUB2 expression is associated with various cancers, potentially affecting tumor development through the regulation of tumor suppressors and promoters [8]. Given its significant biological functions, OTUB2 is considered a potential therapeutic target, particularly in cancer treatment, and efforts are underway to develop strategies targeting OTUB2 [9]. U2AF2 (U2 small nuclear RNA auxiliary factor 2) is a crucial splicing factor involved in pre-mRNA splicing [10, 11]. In partnership with U2AF1, U2AF2 forms a complex that recognizes branch points and polyadenylation signals in pre-mRNA [12, 13]. U2AF2 plays an important role in alternative splicing, influencing the combination of exons and introns, leading to diverse mRNA isoforms and increased protein diversity. Aberrant U2AF2 expression has been linked to several diseases, including cancer, vascular remodeling disorders, and neurodegenerative diseases [14–17].

The ubiquitination-deubiquitination balance refers to the dynamic regulation of protein degradation and stability by the ubiquitin-proteasome system [18]. This process involves the attachment of ubiquitin molecules to target proteins, typically facilitated by E3 ubiquitin ligases, leading to their degradation or functional modification. Ubiquitination is crucial for cell cycle regulation, signal transduction, and cellular stress responses [19–23]. In tumor cells, the balance between ubiquitination and deubiquitination is often disrupted, allowing cancer cells to evade programmed cell death, enhance proliferation, and promote metastasis [19, 24, 25]. Understanding this balance in tumorigenesis could identify novel therapeutic targets for anti-tumor treatments [26].

Ferroptosis is an iron-dependent form of cell death characterized by excessive iron accumulation, lipid peroxidation, and a failure of antioxidant defenses [27, 28]. Unlike apoptosis and necrosis, ferroptosis is closely linked to lipid peroxidation in the cell membrane [29]. Autophagy, the process of cellular self-degradation, involves the encapsulation of damaged organelles and proteins into autophagosomes, which are then degraded after fusion with lysosomes [30]. Recent research has revealed that tumor cell robustness is partly due to their ability to evade ferroptosis, whereas inducing ferroptosis can inhibit tumor growth [31, 32]. Autophagy plays a dual role in tumor biology, as appropriate levels can support cancer cell survival, but in certain contexts, autophagy can enhance ferroptosis [33–35]. For instance, autophagy induction may promote iron accumulation and ROS production, thereby amplifying ferroptosis [36, 37].

In this study, we demonstrate that OTUB2 is highly expressed in various CRC models and stabilizes U2AF2 through deubiquitination, thereby increasing tumor cell resistance to ferroptosis. Inhibition of the OTUB2-U2AF2 axis effectively induces both autophagy and ferroptosis, significantly suppressing the proliferation and migration of CRC cells. Additionally, there is a reciprocal activation relationship between autophagy and ferroptosis in the context of OTUB2-U2AF2 deficiency. In vivo inhibition of the OTUB2-U2AF2 axis significantly impedes tumor growth.

## Materials and methods

### Cell culture and cell lines

We utilized HEK293T cells, normal human colon epithelial mucosal cells (NCM460), and colorectal cancer cell lines LoVo, RKO, SW480, and HT115, all procured from the Shanghai Institute of Life Sciences (Shanghai, China). HEK293T cells were cultured in Dulbecco’s Modified Eagle Medium (DMEM) supplemented with 10% fetal bovine serum (FBS) and 1% penicillin-streptomycin (Life Technologies). The NCM460 cells and colorectal cancer cell lines were maintained in Roswell Park Memorial Institute-1640 (RPMI-1640) medium, also supplemented with 10% FBS and 1% penicillin-streptomycin. All cell cultures were kept at 37 °C in a humidified incubator with 5% CO₂.

### Plasmid constructs, transfections, and lentiviral production

To generate the plasmids Flag-OTUB2, Flag-OTUB2(C51S), and Myc-U2AF2, we inserted the respective full-length cDNAs into 3xFlag-pcDNA3.1 or Myc-pcDNA3.1 vectors. For stable overexpression, OTUB2 and U2AF2 were cloned into the pCDH-puro vector. For knockdown experiments, shRNA targeting OTUB2 (shOTUB2) and a scramble control shRNA were inserted into the pLKO.1-puro vector. HA-tagged ubiquitin constructs, including wild-type ubiquitin, ubiquitin-Lys48 (pRK5-HA-Ub-Lys48), and ubiquitin-Lys63 (pRK5-HA-Ub-Lys63), were obtained from Addgene. Cells were seeded in 6-well plates and allowed to grow for 24 h to reach 70–80% confluence before transfection. Transfections were performed using Lipofectamine 3000 (Invitrogen, catalog number L3000150) according to the manufacturer’s protocol. After 48 h, puromycin was used to select transfected cells for stable expression. For lentiviral packaging, HEK293T cells were co-transfected with psPAX2, pMD2.G, and either shOTUB2-pLKO.1-puro or OTUB2-pCDH-puro. The resulting viral particles were used to infect LoVo, RKO, SW480, and HT115 cells in the presence of 10 μg/mL Polybrene, followed by puromycin selection to establish stable cell lines.

### Immunoprecipitation and in vivo deubiquitination assays

Cells treated under various conditions were lysed in buffer containing protease inhibitors. The lysates were incubated overnight at 4 °C with protein A/G agarose beads (Santa Cruz) and the appropriate antibodies. After washing three times with NETN buffer to remove non-specifically bound proteins, the immunoprecipitated complexes were eluted and analyzed by SDS-PAGE followed by immunoblotting. For ubiquitination studies, cells were co-transfected with combinations of Flag-OTUB2, Flag-OTUB2(C51S), Myc-U2AF2, and HA-Ub constructs (wild-type, Lys48-only, or Lys63-only) and treated with MG132 for 24 h to inhibit proteasomal degradation. Cells were lysed in NETN buffer, and immunoprecipitation was carried out overnight at 4 °C using anti-U2AF2 or anti-Myc antibodies. The ubiquitination levels of U2AF2 were assessed by western blot analysis of the immunoprecipitates.

### In vitro deubiquitination and pull-down assays

To investigate U2AF2 deubiquitination in vitro, HEK293T cells were co-transfected with HA-Ub and Myc-U2AF2 plasmids. Cells were treated with 20 μM MG132 for 8 h to enrich ubiquitinated proteins before harvesting. Ubiquitinated Myc-U2AF2 was immunoprecipitated, and protein concentration was measured using a BCA protein assay kit (Pierce, 23225). One microgram of purified ubiquitinated Myc-U2AF2 was incubated with 1 μg of recombinant GST-OTUB2 (wild-type or C51S mutant) in deubiquitination buffer at 37 °C for 4 h. Subsequently, glutathione sepharose 4B beads (GE Healthcare, 17075601) or anti-Myc beads (ACE, BR0046) were added and incubated at 4 °C for 2 h. The precipitated proteins were then analyzed by western blot using the specified antibodies.

### Western blotting (WB) and antibody information

Proteins were denatured by boiling, separated on 10% SDS-PAGE gels, and transferred onto PVDF membranes. After blocking with 5% non-fat milk for 1 h at room temperature, membranes were incubated overnight at 4 °C with primary antibodies. Membranes were then washed and incubated with HRP-conjugated secondary antibodies for 1 h at room temperature. Detection was performed using enhanced chemiluminescence (ECL), and band intensities were quantified using ImageJ software. Primary antibodies used included OTUB2 (12066-1-AP), U2AF2 (15624-1-AP), GAPDH (60004-1-Ig), ubiquitin (10201-2-AP), Myc-Tag (10828-1-AP), Flag-Tag (20543-1-AP), HA (60004-1-Ig), ACSL4 (81196-1-RR), FTH1 (11682-1-AP), GPX4 (67763-1-Ig), ATG5 (66744-1-Ig), p62 (66184-1-Ig), and LC3 (14600-1-AP), all primarily obtained from Proteintech (Wuhan, China).

### Clinical sample collection

Clinical specimens were collected from patients at the First Affiliated Hospital of University of Science and Technology of China. Cases with insufficient or equivocal pathological/clinical information were excluded. The storage, analysis, and disposal of these samples were conducted in compliance with the guidelines approved by the Clinical Medical Ethics Committee of First Affiliated Hospital of University of Science and Technology of China.

### Colony formation assay

For the colony formation assay, 500 cells were plated per well in 6-well plates and cultured in RPMI-1640 medium with 10% FBS for 2 weeks. The colonies formed were fixed with methanol and stained with 0.1% crystal violet solution (1 mg/mL).

### Transwell migration assay

In Transwell migration assays, 2 × 10⁴ cells were seeded into the upper chambers containing serum-free medium. The lower chambers were filled with DMEM supplemented with 10% FBS to serve as a chemoattractant. After a 24-h incubation, cells that migrated to the lower surface were fixed with 4% paraformaldehyde for 15 min and stained with 0.1% crystal violet. Migrated cells were counted under a microscope in randomly selected fields.

### Cell adhesion assay

HUVEC cells were suspended in endothelial cell medium containing 10% fetal bovine serum (FBS) and seeded at 150,000 cells per well in 24-well plates. After overnight adhesion, cancer cells labeled with CFDA were added. After 3 h, images were taken using an inverted fluorescence microscope to observe the adhesion of cancer cells (green fluorescence) on HUVEC cells (white light), followed by image merging to assess overlap.

### Measurement of iron content, glutathione, and lipid peroxidation levels

Iron levels in cell lysates were measured using a commercial iron assay kit (ab83366, Abcam). Glutathione (GSH) concentrations were determined using a GSH assay kit (CS0260, Sigma), and lipid peroxidation was assessed by measuring malondialdehyde (MDA) levels using a lipid peroxidation assay kit (ab118970, Abcam).

### Molecular probes and cell staining assays

For CFDA staining, treated colorectal cancer cells were incubated with 5 μM CFDA in pre-warmed PBS at 37 °C for 15 min. After replacing the medium, fluorescence was measured within 30 min using a fluorescence microscope with an excitation wavelength of 488 nm. Reactive oxygen species (ROS) levels were detected using the BODIPY 581/591 C11 probe (S0043S, Beyotime), which exhibits a shift from red (reduced form) to green fluorescence (oxidized form) upon oxidation. For cell viability assays, CCK-8 solution (HB-CCK-8-500, HanBio) was added, and absorbance was measured at 450 nm using a microplate reader.

### Animal xenograft models

Male BALB/c nude mice (6–8 weeks old, 18–20 g, *n* = 4/group) were subcutaneously injected with 2 × 10⁷ SW480 cells to establish xenograft tumors. Mice were randomly assigned to treatment groups using a random number table by an independent researcher. Tumor sizes were measured weekly. After 35 days, mice were euthanized under anesthesia, and tumors were excised, weighed, and prepared for histological analysis. Starting 1 week post-inoculation, mice received daily intraperitoneal injections of erastin at 5 mg/kg, while control mice were administered DMSO. Tumor measurements and histological scoring were performed by investigators blinded to group allocation. All animal procedures were performed following NIH guidelines and were approved by the Institutional Animal Care and Use Committee of First Affiliated Hospital of University of Science and Technology of China.

### Statistical analysis

All in vitro experiments were obtained from at least three independently technical repeats. Data points were excluded if technical errors occurred during processing. Statistical analyses were performed using GraphPad Prism version 8.0. Two-tailed Student’s *t* tests were used for comparisons between two groups, while one-way ANOVA followed by Tukey’s post hoc test was applied for multiple group comparisons. Data are presented as mean ± standard deviation (SD). A *p* value of less than 0.05 was considered statistically significant. Sample sizes were determined by power analysis using GPower (*α* = 0.05, power = 0.8).

## Results

### OTUB2 is highly expressed in CRC

**Fig. 1:**
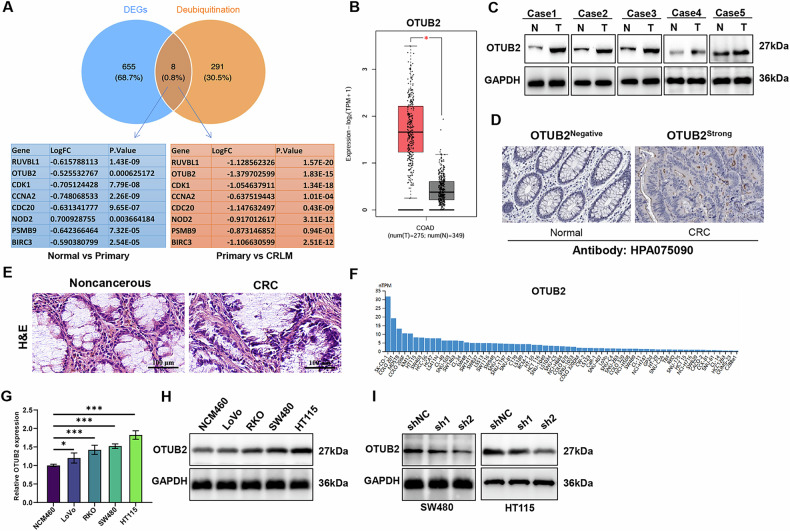
Significant overexpression of OTUB2 in CRC. **A** Venn diagram showing the intersection between differentially expressed genes (DEGs) and deubiquitination genes in CRC. Numbers represent gene counts; percentages indicate their proportion in the total number of genes. **B** Comparison of OTUB2 mRNA expression levels between CRC patients and healthy controls. *p < 0.001. Data obtained from the open-source database GEPIA2. **C** WB analysis of OTUB2 protein expression in tumor tissues and adjacent normal tissues from CRC patients. **D** Immunohistochemical staining of OTUB2 in colonic tissues from normal individuals and CRC patients. Data obtained from the open-source database Human Protein Atlas. **E** Hematoxylin and eosin (H&E) staining of adjacent non-cancerous tissues and cancerous tissues from CRC patients. Scale bar = 100 μm. **F** Ranking of OTUB2 levels in CRC-related cell lines. Data obtained from the open-source database Human Protein Atlas. **G** Quantitative PCR (qPCR) analysis measuring and ranking OTUB2 transcription levels in the normal human colonic mucosal epithelial cell line NCM460 and CRC cell lines LoVo, RKO, SW480, and HT115. *p < 0.05; ***p < 0.001. **H** WB analysis measuring OTUB2 protein levels in the normal cell line NCM460 and CRC cell lines LoVo, RKO, SW480, and HT115. **I** WB results showing the knockdown efficiency of two shRNAs, shOTUB2-1 and shOTUB2-2, on OTUB2 expression in SW480 and HT115 cell lines. All experiments were performed in triplicate. Data are presented as mean ± standard deviation. Statistical significance was determined using one-way ANOVA followed by Tukey’s post hoc test.

Previous studies have suggested that the combination of ferroptosis and autophagy serves as an effective anticancer strategy in cancer cells. To explore and validate this strategy in CRC, we employed bioinformatics approaches to identify potential targets. We obtained a list of human autophagy-related genes from the Human Autophagy Database (HADb), ferroptosis-related genes from the FerrDb V2 database, and deubiquitination-related genes from the Misgdb database. Based on the definitions of gene associations provided by these databases, we analyzed the GSE68468 dataset from the GEO database (Fig. S1). Our analysis identified 663 differentially expressed genes (DEGs) associated with both primary CRC tumors and metastatic tumors (Figs. S1E and 1A). Among these, eight genes were further filtered as being involved in deubiquitination regulation. Data from Gepia2 also revealed that OTUB2 expression levels were higher in colorectal adenocarcinoma tissues (a subtype of CRC) compared to normal tissues (Fig. 1B).

WB analysis of clinical pathological samples confirmed that OTUB2 expression was elevated in CRC tissues compared to adjacent non-tumor tissues (Fig. 1C). Additionally, immunohistochemical results from The Human Protein Atlas demonstrated minimal or absent OTUB2 expression in normal colon tissues, while positive OTUB2 expression was detected in CRC tissues (Fig. 1D). Hematoxylin and eosin (H&E) staining of our clinical samples further showed alterations in crypt structures between cancerous and adjacent non-cancerous tissues (Fig. 1E). We ranked the transcription levels of CRC-related cell lines based on publicly available data (Fig. 1F). Using the normal NCM460 cell line as a control, we examined the transcription levels of OTUB2 in cancer-related LoVo, RKO, SW480, and HT115 cell lines. qPCR (Fig. 1G) and WB (Fig. 1H) analyses confirmed the progressively higher OTUB2 transcription and expression levels in these cell lines. Next, we designed two plasmids (sh-1 and sh-2) to knock down OTUB2 in SW480 and HT115 cell lines, which exhibited higher baseline OTUB2 levels (Fig. 1I).

### OTUB2 knockdown increases ferroptosis and reduces cell viability in CRC cells

**Fig. 2:**
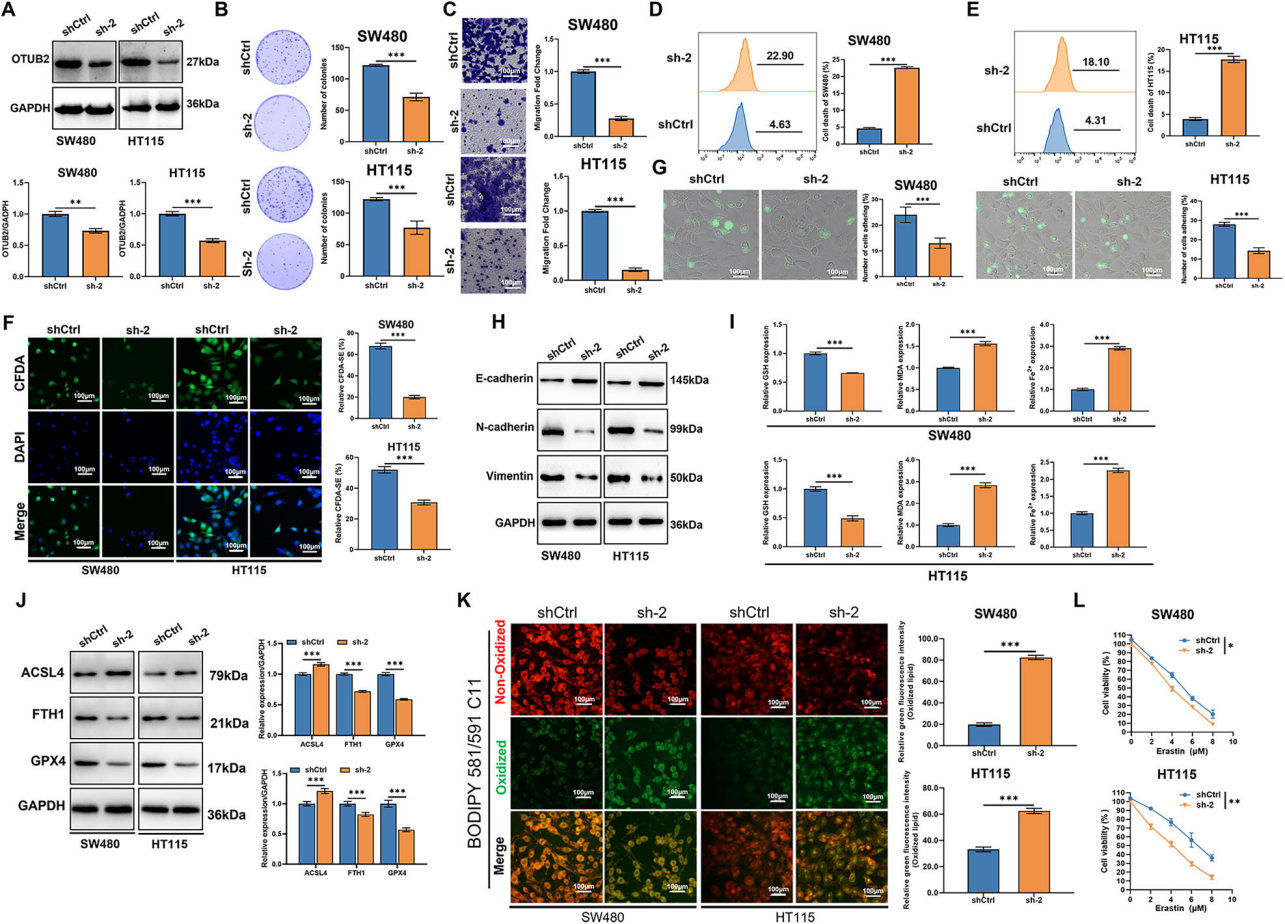
Knockdown of OTUB2 reduces CRC cell survival and increases ferroptosis events. **A** WB results showing the knockdown efficiency of shOTUB2-2, on OTUB2 expression in SW480 and HT115 cell lines. **B** Colony formation assays showing the effect of OTUB2 knockdown on the proliferative capacity of SW480 and HT115 cells. **C** Transwell assays demonstrating the impact of OTUB2 knockdown on the migratory ability of SW480 and HT115 cells. Scale bar = 100 μm. **D** PI staining and flow cytometry analysis indicating the effect of OTUB2 knockdown on cell death in SW480 cells. **E** PI staining and flow cytometry analysis showing the effect of OTUB2 knockdown on cell death in HT115 cells. **F** CFDA and DAPI staining of SW480 and HT115 cells after OTUB2 knockdown, illustrating cell viability proportions. Scale bar = 100 μm. **G** Endothelial adhesion assays demonstrating the effect of OTUB2 knockdown on the metastatic adhesion capability of SW480 cells. Scale bar = 100 μm. **H** WB analysis showing the impact of OTUB2 knockdown on the expression levels of epithelial-mesenchymal transition-related proteins E-Cadherin, N-Cadherin, and Vimentin in HT115 cells. **I** Effects of OTUB2 knockdown on ferroptosis-related biochemical indicators GSH, Fe²⁺, and MDA in SW480 and HT115 cells. **J** Impact of OTUB2 knockdown on ferroptosis-related proteins ACSL4, FTH1, and GPX4 in SW480 and HT115 cells. **K** BODIPY C11 probe staining displaying intracellular ROS levels in SW480 and HT115 cell lines after OTUB2 knockdown. Scale bar = 100 μm. **L** CCK-8 assays indicating the cell viability of SW480 and HT115 cells treated with different concentrations of Erastin following OTUB2 knockdown. All experiments were performed in triplicate. Data are presented as mean ± standard deviation. Statistical significance was determined using one-way ANOVA followed by Tukey’s post hoc test. *p < 0.05, **p < 0.01, ***p < 0.001 compared to control groups.

Given the marked overexpression of OTUB2 in CRC tissues and cell lines, we next investigated its functional impact on malignant phenotypes and ferroptosis. We first transfected SW480 and HT115 cells with the sh-2 plasmid, which exhibited higher knockdown efficiency, and successfully constructed the OTUB2 knockdown cell model, as confirmed by Western blot (Fig. 2A). Knockdown of OTUB2 in SW480 and HT115 cell lines significantly reduced colony formation in colony formation assays, indicating a decrease in cellular proliferative capacity (Fig. 2B). In migration assays, OTUB2 knockdown resulted in a marked reduction in the migration ability of cancer cells (Fig. 2C). Flow cytometry analysis of PI-stained cancer cells revealed increased cell death following OTUB2 knockdown (Fig. 2D, E). This finding was further confirmed by microscopy, where CFDA staining of live cells demonstrated a reduced proportion of viable cells, consistent with the PI flow cytometry results (Fig. 2F). To assess the ability of cancer cells to colonize during metastasis, we performed an endothelial cell adhesion assay, simulating the interaction between tumor cells and vascular endothelial cells in vitro. The results indicated that OTUB2 knockdown significantly impaired tumor cell adhesion to endothelial cells (Fig. 2G), suggesting that the expression or transport of adhesion proteins on the cell membrane was effectively inhibited. We subsequently examined the expression of E-cadherin, N-cadherin, and Vimentin. The results showed a significant increase in E-cadherin expression, alongside reductions in N-cadherin and Vimentin levels (Fig. 2H), supporting the hypothesis that OTUB2 knockdown suppresses epithelial-mesenchymal transition (EMT).

In terms of ferroptosis, OTUB2 knockdown significantly reduced glutathione (GSH) levels, a key indicator of cellular antioxidant potential, while increasing malondialdehyde (MDA) and Fe^2+^ levels, both essential for ferroptosis (Fig. 2I). Additionally, WB analysis showed a significant increase in ACSL4, a protein positively correlated with ferroptosis, along with a marked depletion of ferritin heavy chain (FTH), which is crucial for iron homeostasis. GPX4, an antioxidant protein, was also significantly reduced (Fig. 2J). To further investigate the effects of OTUB2 knockdown on reactive oxygen species (ROS), we used the C11 probe, which fluoresces red in its non-oxidized state and green when oxidized by ROS. The intensity of green fluorescence, reflecting intracellular ROS levels, was dramatically increased in tumor cells following OTUB2 knockdown, indicating a substantial rise in ROS levels (Fig. 2K). We treated the tumor cells with the ferroptosis inducer Erastin and counted the viable cells using CCK-8 staining. The results showed that, under the same concentration of Erastin, the OTUB2-knockdown cells underwent significantly more ferroptosis compared to the control cells (Fig. 2L). This indicates that OTUB2 knockdown not only activates ferroptosis but also sensitizes cancer cells to ferroptosis inducers. These results suggest that OTUB2 knockdown enhances ferroptosis and increases CRC cell sensitivity to ferroptosis-inducing agents.

In addition, we overexpressed OTUB2 in LoVo and RKO cell lines, which had lower baseline OTUB2 levels, using plasmids containing the OTUB2 gene sequence. WB experiments confirmed the successful construction of OTUB2-overexpressing CRC cells (Fig. S2A). Conversely, the overexpression of OTUB2 in CRC strengthens iron homeostasis and antioxidant potential (Fig. S2G–I), thereby conferring resistance to ferroptosis (Fig. S2J). Beyond cell survival and death, OTUB2 expression also regulates cancer cell viability (Fig. S2D), influencing proliferation (Fig. S2B), migration (Fig. S2C), and EMT (Fig. S2E, F). In summary, the above results suggest that OTUB2 affects the proliferation and migration levels of CRC cells by regulating the ferroptosis process.

### OTUB2 regulates autophagy in CRC cells

**Fig. 3:**
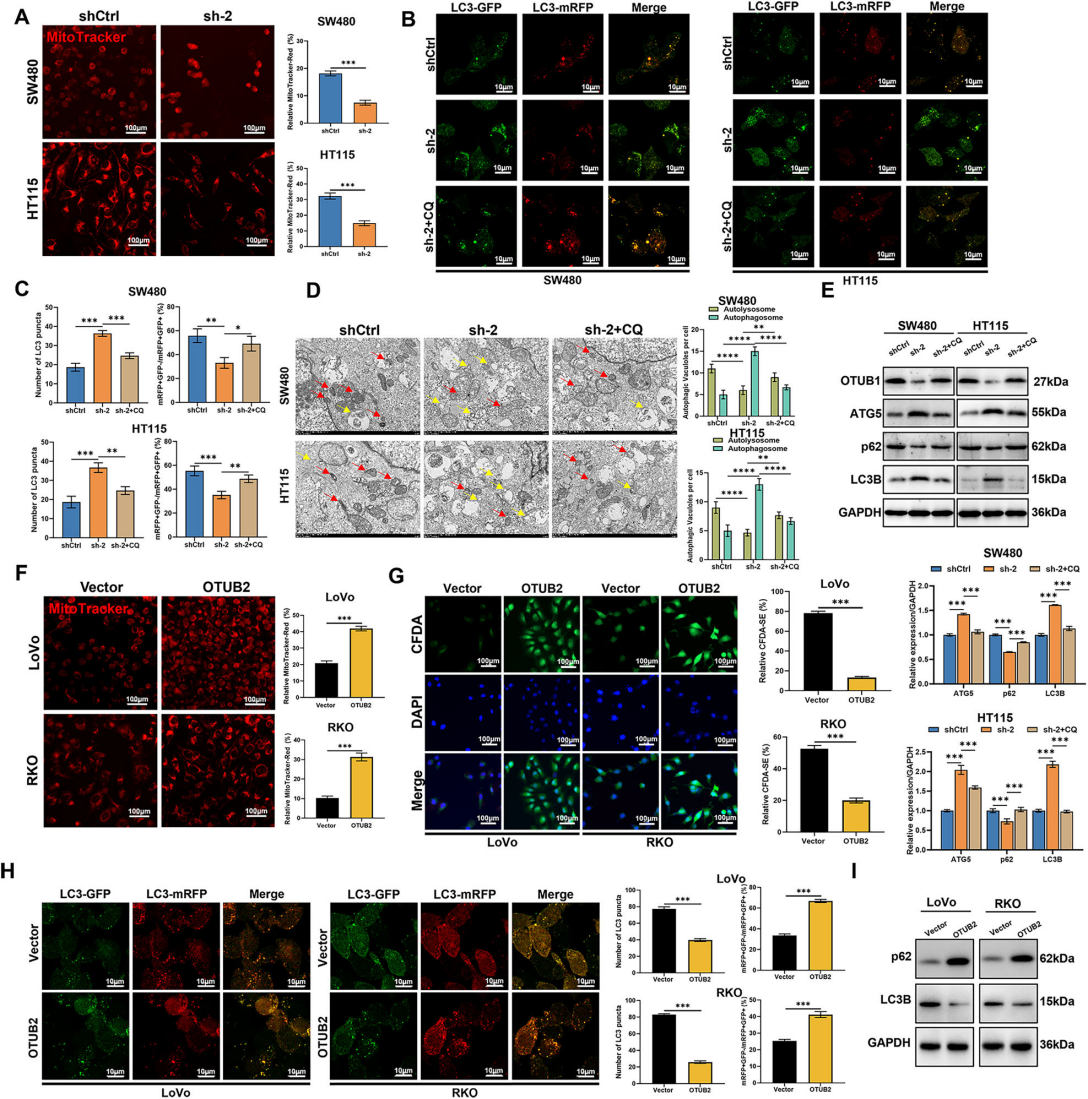
OTUB2 negatively regulates autophagosome initiation in tumor cells. **A** Visualization of mitochondria in SW480 and HT115 cells after OTUB2 knockdown using the MitoTracker probe. Scale bar = 100 μm. **B** mRFP-GFP-LC3 dual fluorescence labeling showing autophagosomes (yellow puncta indicating colocalization of red and green fluorescence) and autolysosomes (red-only puncta) in SW480 and HT115 cells following OTUB2 knockdown or/and CQ treatment. Scale bar = 10 μm. **C** Bar graphs representing the fluorescence intensity of mRFP-GFP-LC3 dual labeling in SW480 and HT115 cells after OTUB2 knockdown or/and CQ treatment. **D** Transmission electron microscopy images displaying autophagosomes in SW480 and HT115 cells post OTUB2 knockdown or/and CQ treatment. Scale bar = 1 μm. **E** WB analysis showing the expression levels of autophagy-related proteins ATG5, p62, and LC3 in SW480 cells after OTUB2 knockdown or/and CQ treatment. **F** Visualization of mitochondria in LoVo and RKO cells following OTUB2 overexpression using the MitoTracker probe. Scale bar = 100 μm. **G** CFDA and DAPI staining of LoVo and RKO cells after OTUB2 overexpression, indicating cell viability. Scale bar = 100 μm. **H** mRFP-GFP-LC3 dual fluorescence labeling showing autophagosomes (yellow puncta) and autolysosomes (red-only puncta) in LoVo and RKO cells following OTUB2 overexpression. Scale bar = 10 μm. **I** WB analysis displaying the expression levels of autophagy-related proteins p62 and LC3 in SW480 cells after OTUB2 knockdown and CQ treatment. All experiments were performed in triplicate. Data are presented as mean ± standard deviation. Statistical significance was determined using one-way ANOVA followed by Tukey’s post hoc test. *p < 0.05, **p < 0.01, ***p < 0.001 compared to control groups.

To further elucidate the mechanism by which OTUB2 regulates the ferroptosis process, it is known that ferroptosis activation is often accompanied by mitochondrial dysfunction. Subsequently, we treated tumor cells with MitoTracker staining to assess mitochondrial activity. Upon OTUB2 knockdown in SW480 and HT115 cells, mitochondrial signals diminished (Fig. 3A). Conversely, overexpression of OTUB2 in LoVo and RKO cells, which have lower baseline OTUB2 levels, resulted in increased mitochondrial signals (Fig. 3F). This indicates that OTUB2 positively regulates mitochondrial activity. To eliminate damaged mitochondria and mitigate ROS production, cells must activate appropriate responses, including autophagy [38]. We employed an mRFP-GFP-LC3 dual fluorescence-tagged lentiviral probe to track autophagic activity. LC3, an autophagy-related protein, localizes to the inner membranes of autophagosomes and autolysosomes. The red fluorescent mRFP tag is unstable and present only in neutral autophagosomes, whereas GFP is more stable and exhibits green fluorescence in both neutral autophagosomes and acidic autolysosomes. By measuring fluorescence intensity and the ratio of red to green signals, we can evaluate autophagic flux and the relative proportion of autophagosomes to autolysosomes.

OTUB2 knockdown increased the number of LC3-positive puncta, while the ratio of mRFP^+^GFP^−^ to mRFP^+^GFP^+^ puncta decreased, indicating enhanced autophagy, with a greater increase in autophagosome formation compared to their fusion with lysosomes (Fig. 3B, C). This suggests that the accumulation of autophagosomes in OTUB2 knockdown cells is a primary driver of the observed increase in autophagic activity. In contrast, OTUB2 overexpression reduced the number of LC3-positive puncta, while the autolysosome-to-autophagosome ratio increased significantly, suggesting that OTUB2 overexpression may reduce autophagy by limiting autophagosome formation (Fig. 3H). Transmission electron microscopy confirmed these results, showing an increase in autophagosomes and a decrease in autolysosomes following OTUB2 knockdown (Fig. 3D), consistent with the red-green fluorescence ratio. WB analysis of autophagy-related proteins further confirmed that OTUB2 negatively regulates autophagy in CRC cells (Fig. 3E, I). Crucially, autophagy inhibitor chloroquine (CQ) rescue in OTUB2 knockdown cells reversed the enhanced autophagic activity (Fig. 3B–E). As OTUB2 overexpression reduced autophagy, cell survival rates in LoVo and RKO cells increased (Fig. 3G). In summary, these results indicate that OTUB2 has a negatively regulatory effect on autophagy.

### Crosstalk between OTUB2-mediated ferroptosis and autophagy

**Fig. 4:**
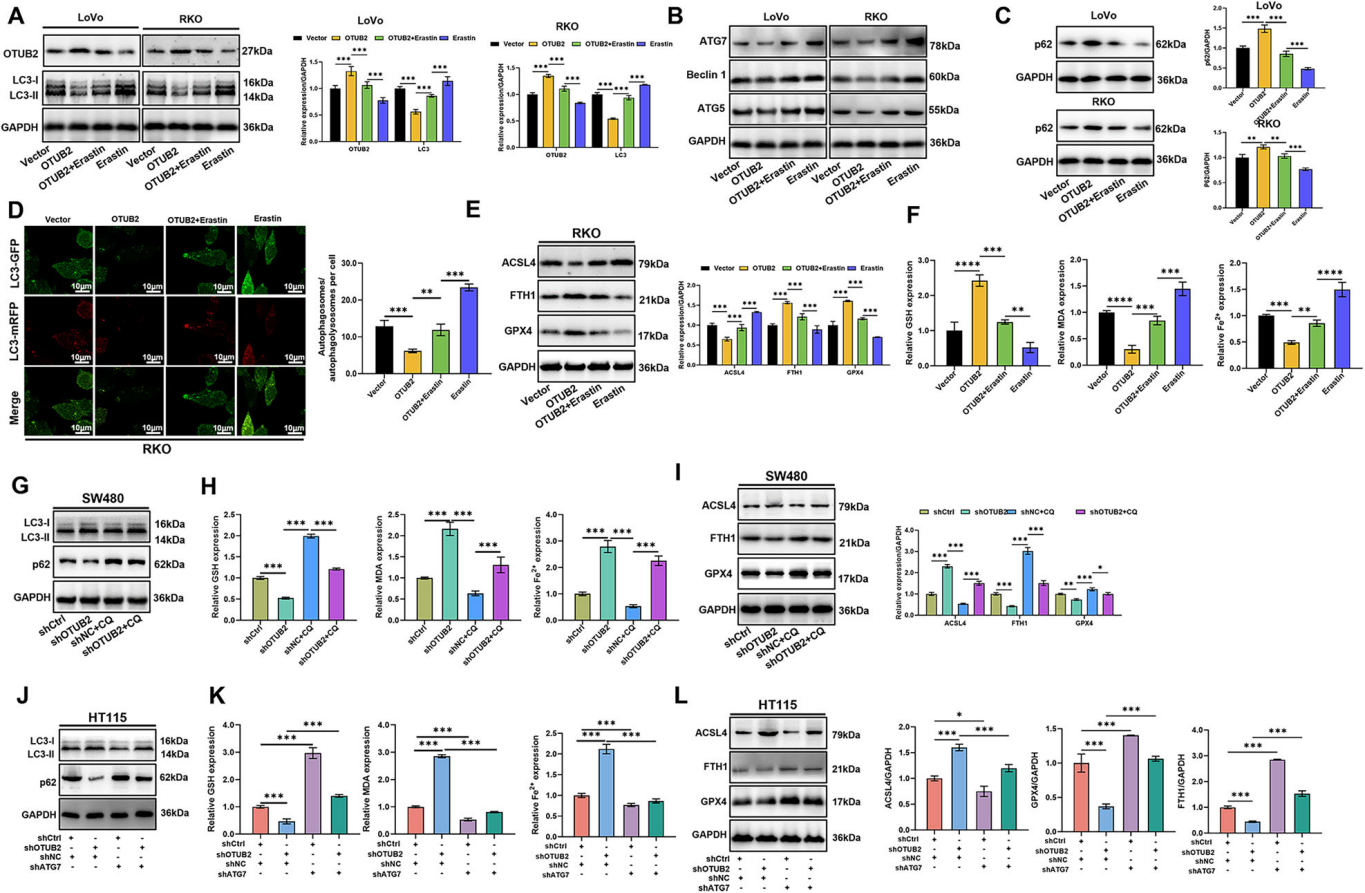
OTUB2-mediated ferroptosis and autophagy mutually positively regulate each other. **A** WB analysis showing the expression levels of the autophagosome maturation-related protein LC3 in LoVo and RKO cells after overexpression of OTUB2 and treatment with Erastin. **B** WB results displaying the expression of autophagy initiation and formation regulatory proteins ATG7, Beclin 1, and ATG5 in LoVo and RKO cells following OTUB2 overexpression and Erastin treatment. **C** WB analysis indicating the expression of the autophagosome maturation-related protein p62 in LoVo and RKO cells after OTUB2 overexpression and Erastin treatment. **D** mRFP-GFP-LC3 dual fluorescence labeling in RKO cells showing autophagosomes (yellow puncta indicating colocalization of red and green fluorescence) and autolysosomes (red-only puncta) after OTUB2 overexpression and Erastin treatment. Scale bar = 10 μm. **E** Immunoblotting showing the expression of ferroptosis-related proteins ACSL4, FTH1, and GPX4 in RKO cells after OTUB2 overexpression and Erastin treatment. **F** Biochemical assays measuring the relative intracellular levels of ferroptosis-related molecules GSH, MDA, and Fe^2+^ in RKO cells after OTUB2 overexpression and Erastin treatment. **G** WB analysis of autophagosome maturation and degradation-related proteins LC3 and p62 in SW480 cells following OTUB2 knockdown and CQ treatment. **H** Biochemical assays measuring the relative intracellular levels of ferroptosis-related molecules GSH, MDA, and Fe^2+^ in SW480 cells after OTUB2 knockdown and CQ treatment compared to control groups. **I** Immunoblotting showing the expression of ferroptosis-related proteins ACSL4, FTH1, and GPX4 in SW480 cells following OTUB2 knockdown and CQ treatment. **J** WB analysis of autophagosome maturation and degradation-related proteins LC3 and p62 in HT115 cells after simultaneous knockdown of OTUB2 and ATG7. **K** Biochemical assays measuring the relative intracellular levels of ferroptosis-related molecules GSH, MDA, and Fe²⁺ in HT115 cells after simultaneous knockdown of OTUB2 and ATG7. **L** Immunoblotting showing the expression of ferroptosis-related proteins ACSL4, FTH1, and GPX4 in HT115 cells after simultaneous knockdown of OTUB2 and ATG7. All experiments were performed in triplicate. Data are presented as mean ± standard deviation. Statistical significance was determined using one-way ANOVA followed by Tukey’s post hoc test. **p < 0.01; ***p < 0.001 compared to control groups.

Having confirmed that OTUB2 independently negatively regulates both ferroptosis and autophagy in CRC cells, we investigated whether the ferroptosis and autophagy activated by OTUB2 knockdown are interconnected. We overexpressed OTUB2 in LoVo and RKO cells and introduced the ferroptosis inducer Erastin. WB analysis revealed that the expression of the autophagy marker protein LC3 and OTUB2 significantly increased with Erastin treatment (Fig. 4A). The autophagy-related protein ATG5, Beclin 1 and ATG7 also showed significant upregulation under the influence of Erastin (Fig. 4B). Conversely, levels of the autophagy-negative regulator p62 increased with OTUB2 overexpression but decreased following Erastin treatment (Fig. 4C). Results from the mRFP-GFP-LC3 dual fluorescence assay indicated that Erastin treatment led to a substantial increase in autophagosomes in RKO cells, while OTUB2 overexpression counteracted the Erastin-induced increase in autophagosome formation (Fig. 4D). Furthermore, we further demonstrated that OTUB2 overexpression inhibits ferroptosis, based on decreased expression of ferroptosis markers and altered iron metabolism profiles, while Erastin treatment triggered ferroptotic activation (Fig. 4E, F). These findings indicate that Erastin-induced ferroptosis promotes autophagy in LoVo and RKO cells, and this ferroptosis-dependent autophagy is inhibited by OTUB2.

To further explore this relationship, we modulated the autophagy negatively regulated by OTUB2 by treating SW480 cells, where autophagy was enhanced due to OTUB2 knockdown, with CQ. WB analysis showed a significant increase in p62 after CQ treatment, indicating effective autophagy inhibition (Fig. 4G). Analysis of key biochemical markers of ferroptosis revealed that GSH, representing cellular antioxidant potential, significantly decreased under OTUB2 knockdown; however, this effect was markedly suppressed by CQ treatment. MDA and Fe^2+^ levels significantly increased with OTUB2 knockdown and subsequently decreased after CQ treatment, though not completely reversing the increases caused by OTUB2 knockdown (Fig. 4H). This suggests that OTUB2-dependent autophagy positively cross-talks with ferroptosis, and inhibition of autophagy suppresses ferroptosis. WB analysis of ferroptosis-related proteins showed significant decreased in ACSL4 levels, while FTH1 and GPX4 levels significantly increased after CQ treatment. Moreover, regardless of CQ treatment, OTUB2 knockdown consistently reduced FTH1 and GPX4 levels (Fig. 4I).

Beyond pharmacological intervention, we also inhibited autophagy by knocking down the essential autophagy protein ATG7. WB analysis indicated that following the ATG7 knockdown, LC3 levels significantly decreased and p62 levels significantly increased under both OTUB2 knockdown and control conditions (Fig. 4J), indicating effective inhibition of autophagy. Analysis of ferroptosis markers revealed that GSH levels, which decreased significantly under OTUB2 knockdown, sharply increased after ATG7 knockdown. Interestingly, in the absence of ATG7 knockdown, OTUB2 knockdown led to increased MDA and Fe^2+^ levels; however, with ATG7 knockdown, OTUB2 knockdown did not result in increased MDA and Fe^2+^ levels (Fig. 4K). This suggests that the inhibition of autophagosome initiation by ATG7 occurs upstream of OTUB2’s negative regulation. Correspondingly, WB results showed that after ATG7 knockdown, ACSL4 levels decreased, while FTH1 and GPX4 levels increased, with these changes being insensitive to OTUB2 knockdown (Fig. 4L). This indicates that OTUB2 knockdown activates autophagosome initiation, promoting the degradation of FTH1 to release iron ions, degrading GPX4 to disrupt antioxidant defenses, and enhancing MDA accumulation through ACSL4 pathways, thereby increasing lipid peroxidation levels and ultimately promoting ferroptosis. In summary, OTUB2 knockdown independently activates both ferroptosis and autophagy, and the ferroptosis and autophagy induced by OTUB2 knockdown can mutually enhance each other without a unidirectional dependency.

### OTUB2 stabilizes U2AF2 through deubiquitination

**Fig. 5:**
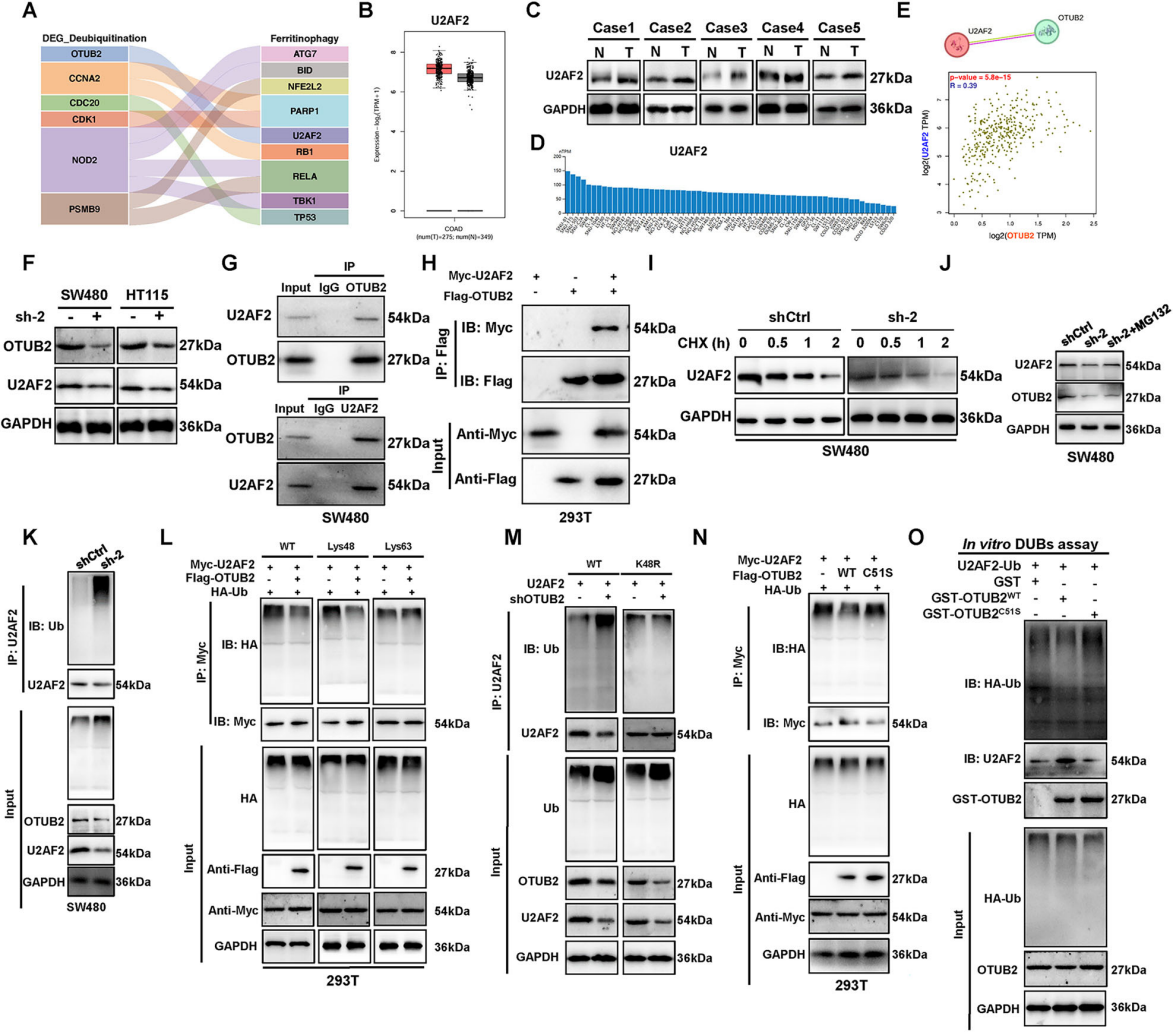
OTUB2 deubiquitinates U2AF2, preventing its degradation via the proteasomal pathway. **A** Sankey diagram illustrating the association between differentially expressed deubiquitinating enzyme genes and ferroptosis-related genes in CRC. **B** Comparison of U2AF2 mRNA expression levels between CRC patients and healthy controls. **C** WB analysis of U2AF2 protein expression in tumor tissues and adjacent normal tissues from CRC patients. **D** Ranking of OTUB2 levels in CRC cell lines, obtained from the open-source database Human Protein Atlas. **E** Correlation between OTUB2 and U2AF2, derived from the open-source database STRING. **F** WB results showing the effect of shOTUB2-2 knockdown on U2AF2 levels in SW480 and HT115 cell lines. **G** Co-IP of U2AF2 using anti-OTUB2 antibody in SW480 cells (upper panel), and Co-IP of OTUB2 using anti-U2AF2 antibody (lower panel). **H** SW480 cells were transfected with Myc-tagged U2AF2 and Flag-tagged OTUB2; cell lysates were immunoprecipitated with anti-Flag antibody, followed by immunoblotting with anti-Myc (U2AF2) and anti-Flag (OTUB2) antibodies. **I** SW480 cells transfected with sh-Ctrl or shOTUB2-2 were treated with CHX (10 μg/mL) for different durations; remaining intracellular U2AF2 protein levels were measured. **J** SW480 cells with OTUB2 knockdown were treated with MG132; levels of U2AF2 and OTUB2 were determined by WB. **K** After OTUB2 knockdown in SW480 cells, cell lysates were immunoprecipitated with anti-U2AF2 antibody, followed by immunoblotting with anti-ubiquitin and anti-U2AF2 antibodies to assess U2AF2 ubiquitination and residual protein levels post-degradation. **L** Under treatment with the proteasome inhibitor MG132, HEK 293 T cells were co-transfected with Myc-U2AF2, Flag-OTUB2, and plasmids encoding Ub WT, Ub Lys48-only, or Ub Lys63-only; subsequently, ubiquitination levels of U2AF2 were analyzed. **M** In control and OTUB2-knockdown HEK 293 T cells expressing Ub WT or Ub Lys48R, after 72 h of culture, ubiquitination levels of U2AF2 were analyzed. **N** Cell lysates from HEK 293 T cells transfected with HA-tagged ubiquitin, Myc-tagged U2AF2, and either Flag-tagged OTUB2 (WT) or Flag-tagged OTUB2 (C51S) were immunoprecipitated with anti-Myc antibody, followed by immunoblotting with anti-HA and anti-Myc antibodies. **O** In vitro deubiquitination assay assessing purified ubiquitinated U2AF2 incubated with purified GST-OTUB2-WT or GST-OTUB2-C51S. Experiments were independently repeated three times with similar results. All experiments were performed in triplicate. Data are presented as mean ± standard deviation. Statistical significance was determined using one-way ANOVA followed by Tukey’s post hoc test. *p < 0.05; **p < 0.01; ***p < 0.001 compared to control groups.

To investigate the mechanism by which OTUB2 negatively regulates autophagy and ferroptosis, we analyzed DEGs related to deubiquitation obtained from our previous screening and assessed their correlation with known ferroptosis-related genes. We identified a correlation between OTUB2 and U2AF2 [8] (Fig. 5A, E). Online database analyses supported this finding, showing that the transcription level of U2AF2 in CRC tissues was slightly elevated compared to normal tissues, although not significantly different (Fig. 5B). WB analysis of U2AF2 protein levels in paired patient samples of adjacent non-cancerous and cancerous tissues revealed a significant increase in U2AF2 levels in CRC tissues (Fig. 5C). Based on the ranking of U2AF2 levels in CRC-related cell lines (Fig. 5D), we selected SW480 and HT115 cell lines, which exhibited moderate to high U2AF2 expression. Knockdown of OTUB2 in these cells resulted in a notable decrease in U2AF2 and OTUB2 levels (Fig. 5F).

In co-immunoprecipitation (Co-IP) experiments, U2AF2 was detected in protein complexes captured by anti-OTUB2 antibodies, and reciprocally, OTUB2 was detected in complexes precipitated with anti-U2AF2 antibodies (Fig. 5G). Additionally, we transiently transfected HEK293T cells with tagged recombinant full-length proteins for Co-IP assays. Flag-tagged OTUB2 and Myc-tagged U2AF2 were successfully expressed and effectively co-precipitated in HEK293T cells (Fig. 5H). These endogenous and exogenous OTUB2-U2AF2 co-precipitation experiments confirmed a direct interaction between OTUB2 and U2AF2. Given that the protein levels of U2AF2 differed significantly, we hypothesized that U2AF2 stability was regulated post-translationally, leading to differences in protein abundance. We used cycloheximide (CHX) to inhibit protein synthesis and observed the net degradation rate of U2AF2. As expected, U2AF2 degradation was significantly accelerated upon OTUB2 knockdown (Fig. 5I). Considering that OTUB2 is a deubiquitinating enzyme, we speculated that OTUB2 regulates U2AF2 stability via the ubiquitin-proteasome pathway. Therefore, we treated cells with the proteasome inhibitor MG132. WB results showed that MG132 treatment partially restored U2AF2 levels but did not affect the reduced OTUB2 levels due to knockdown, suggesting that the decrease in U2AF2 caused by OTUB2 knockdown might be related to ubiquitin-mediated degradation (Fig. 5J).

We assessed the ubiquitination level of U2AF2 in SW480 cells with or without OTUB2 knockdown using Co-IP. The result indicated that U2AF2 ubiquitination significantly increased following OTUB2 knockdown (Fig. 5K). To determine which ubiquitin linkage OTUB2 targets in its interaction with U2AF2, we introduced HA-tagged recombinant ubiquitin constructs—HA-Ub-WT, HA-Ub-K48-only, and HA-Ub-K63-only—into HEK293T cells. In the presence of OTUB2, ubiquitin conjugated to U2AF2 decreased for both the WT and K48-only ubiquitin constructs (Fig. 5L). Subsequently, we introduced a mutant ubiquitin resistant to K48 linkage (K48R) into SW480 cells. Under OTUB2 knockdown conditions, wild-type ubiquitin promoted U2AF2 degradation and ubiquitination level, whereas the K48R mutant did not reduce U2AF2 level and the U2AF2 ubiquitination level remained unchanged (Fig. 5M). This demonstrated that lysine 48 (K48) is the critical site for U2AF2 degradation, and that OTUB2 deubiquitinates K48-linked ubiquitin chains on U2AF2. Next, we sought to confirm the active site of OTUB2 responsible for its deubiquitinating activity. We introduced a catalytically inactive mutant OTUB2 (C51S) into cells. Co-IP experiments (Fig. 5N) and GST pull-down assays (Fig. 5O) showed that the C51S mutant failed to reduce ubiquitin attached to U2AF2. In summary, OTUB2 stabilizes U2AF2 by deubiquitinating K48-linked ubiquitin chains on U2AF2 via its catalytic cysteine at residue 51. This stabilization increases U2AF2 levels, enhancing its functions in inhibiting ferroptosis and autophagy.

### Knockdown of U2AF2 inhibits the proliferative phenotype induced by OTUB2 overexpression in tumor cells

**Fig. 6:**
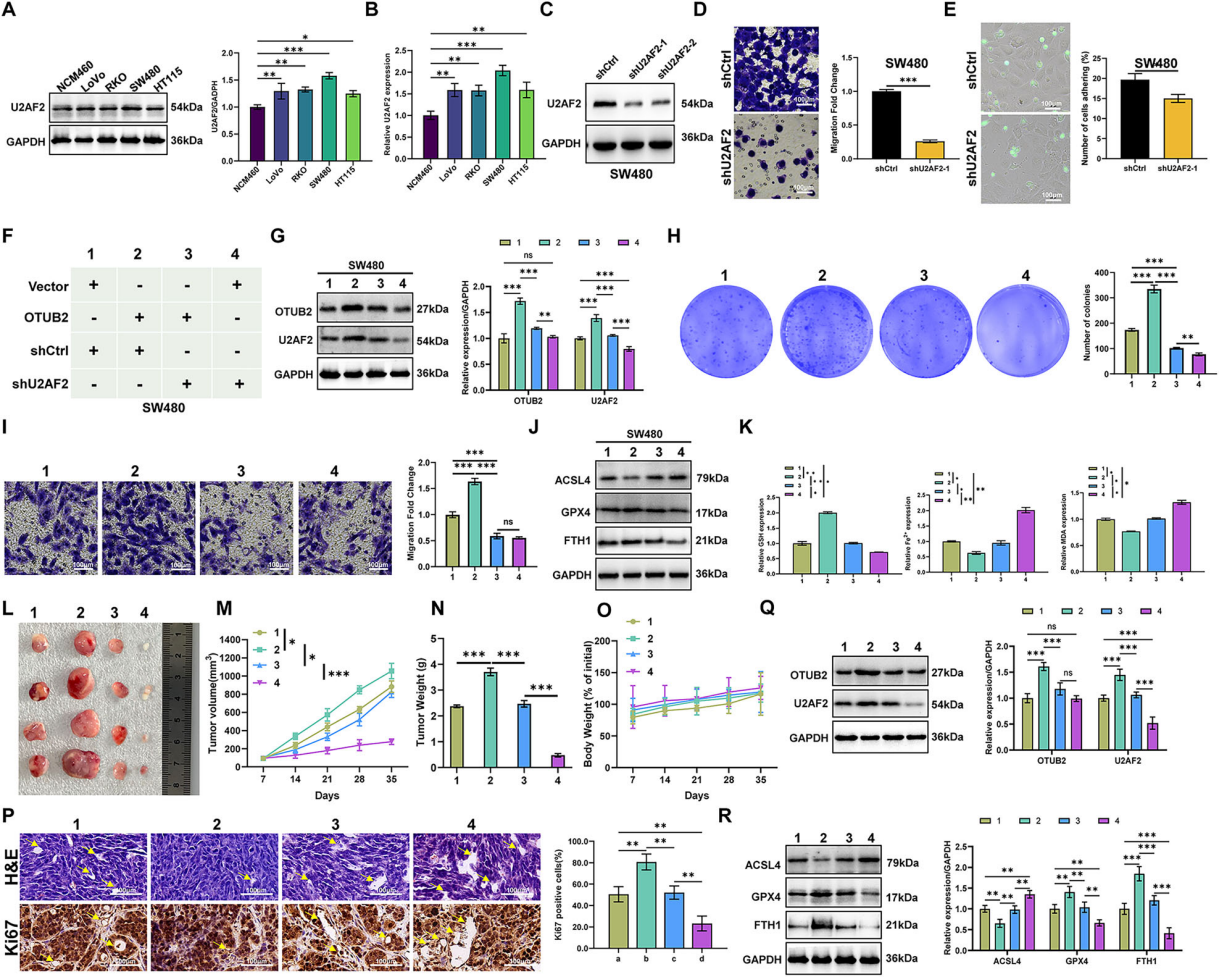
Knockdown of U2AF2 inhibits CRC cells in vitro and in vivo. **A** The protein levels of U2AF2 in the normal cell line NCM460 and CRC cell lines LoVo, RKO, SW480, and HT115 were measured by WB. **B** The transcription levels of U2AF2 in the normal cell line NCM460 and CRC cell lines LoVo, RKO, SW480, and HT115 were measured by qPCR. **C** WB results showing the efficiency of U2AF2 knockdown by two shRNAs, shU2AF2-1 and shU2AF2-2, in SW480 cells. **D** Transwell assays demonstrating the effect of U2AF2 knockdown on the migration ability of SW480 cells. Scale bar = 100 μm. **E** Endothelial cell adhesion assays showing the impact of U2AF2 knockdown on the cell surface adhesion capability of SW480 cells. Scale bar = 100 μm. **F** SW480 cells were subjected to treatments of OTUB2 overexpression and U2AF2 knockdown, respectively. **G** WB analysis showing the expression levels of OTUB2 and U2AF2 proteins in cultured SW480 cells after OTUB2 overexpression or/and U2AF2 knockdown treatments. **H** Colony formation assays indicating the effects of OTUB2 overexpression and U2AF2 knockdown on the proliferation of SW480 cells. **I** Transwell assays displaying the effects of OTUB2 overexpression and U2AF2 knockdown on the migration ability of SW480 cells. Scale bar = 100 μm. **J** WB analysis showing the expression levels of ferroptosis-related proteins in cultured SW480 cells after OTUB2 overexpression or/and U2AF2 knockdown treatments. **K** Levels of ferroptosis-related markers GSH, Fe²⁺, and MDA in cultured SW480 cells after OTUB2 overexpression or/and U2AF2 knockdown treatments. **L** Representative images of tumors formed 35 days after subcutaneous inoculation of SW480 cells into nude mice following OTUB2 overexpression and U2AF2 knockdown treatments. **M** Tumor growth curves depicting volume changes over time in SW480 cell-derived tumors after OTUB2 overexpression and U2AF2 knockdown treatments. **N** Average tumor weights of SW480 cell-derived tumors following OTUB2 overexpression and U2AF2 knockdown treatments. **O** The body weight of nude mice during this experiment. **P** Representative images of H&E-stained tumor sections from SW480 cell-derived tumors after OTUB2 overexpression and U2AF2 knockdown treatments. Scale bar = 100 μm. **Q** WB analysis showing the expression levels of OTUB2 and U2AF2 proteins in SW480 cell-derived tumors after OTUB2 overexpression and U2AF2 knockdown treatments. **R** WB analysis showing the expression levels of ferroptosis-related proteins in SW480 cell-derived tumors after OTUB2 overexpression and U2AF2 knockdown treatments. All experiments were performed in triplicate. Data are presented as mean ± standard deviation. Statistical significance was determined using one-way ANOVA followed by Tukey’s post hoc test. *p < 0.05, **p < 0.01, ***p < 0.001 compared to control groups.

Having established that OTUB2 positively regulates U2AF2 through deubiquitination and that OTUB2 negatively influences the survival rate of CRC tumor cells via ferroptosis, we hypothesized that U2AF2, regulated by OTUB2, might also participate in ferroptosis regulation and ultimately affect tumor phenotypes. We assessed U2AF2 levels in normal NCM460 cells and CRC-related cell lines LoVo, RKO, SW480, and HT115. The SW480 cell line, exhibiting the highest baseline level of U2AF2, was selected for knockdown experiments (Fig. 6A–C). Transwell migration assays and cell adhesion assays were performed on SW480 cells with U2AF2 knockdown. The results showed significant reductions in both cell migration (Fig. 6D) and adhesion abilities (Fig. 6E), mirroring the phenotypes observed with OTUB2 knockdown (Fig. 2C, G).

Subsequently, we knocked down U2AF2 in cells overexpressing OTUB2 (Fig. 6F). WB assays confirmed the expression of OTUB2 and U2AF2 (Fig. 6G). In colony formation and Transwell migration assays, U2AF2 knockdown not only counteracted the proliferative and migratory phenotypes induced by OTUB2 overexpression but also resulted in significantly less proliferation and migration compared to the control group without OTUB2 overexpression (Fig. 6H). In migration assays, the reduction in migration caused by U2AF2 knockdown could not be compensated for by OTUB2 overexpression (Fig. 6I), indicating that U2AF2 has a higher regulatory priority over tumor cell proliferation and migration. In ferroptosis-related analyses, U2AF2 knockdown led to a significant increase in ACSL4 and a significant decrease in GPX4 levels (Fig. 6J). GSH levels decreased markedly, while MDA and Fe²⁺ levels increased significantly in U2AF2 knockdown cells (Fig. 6K), indicating enhanced ferroptosis. Notably, the effects of OTUB2 overexpression and U2AF2 knockdown on ferroptosis were comparable, suggesting that U2AF2 may regulate tumor cell proliferation and migration through ferroptosis-independent pathways.

To further elucidate the in vivo functions of OUTB2 and U2AF2, we transplanted these treated cells subcutaneously into mice, an in vivo tumor development model was established (Fig. 6L). The tumor volume growth curves clearly showed that the cell line with U2AF2 knockdown alone exhibited the slowest growth. U2AF2 knockdown suppressed the accelerated tumor cell proliferation induced by OTUB2 overexpression, resulting in growth similar to the control group (Fig. 6M). The final tumor weights corroborated the growth curve measurements (Fig. 6N). Additionally, there is no significant difference in body weight was noted between the four groups (Fig. 6O). Histological staining of tumor sections revealed that the degree of cavitation within tumors was proportional to the ferroptosis levels observed in previous experiments (Fig. 6P). OTUB2 overexpression in tumor tissues significantly increased OTUB2 levels and concurrently inhibited ferroptosis through ACSL4 downregulation with concomitant GPX4/FTH1 upregulation. Crucially, these anti-ferroptotic effects were negated by U2AF2 knockdown (Fig. 6Q, R), confirming U2AF2’s essential role in OTUB2-mediated ferroptosis suppression. These findings indicate that targeting and inhibiting U2AF2 in CRC tumors with high OTUB2 expression can produce significant anticancer effects.

### OTUB2 promotes tumorigenesis by enhancing resistance to ferroptosis in CRC cells

**Fig. 7:**
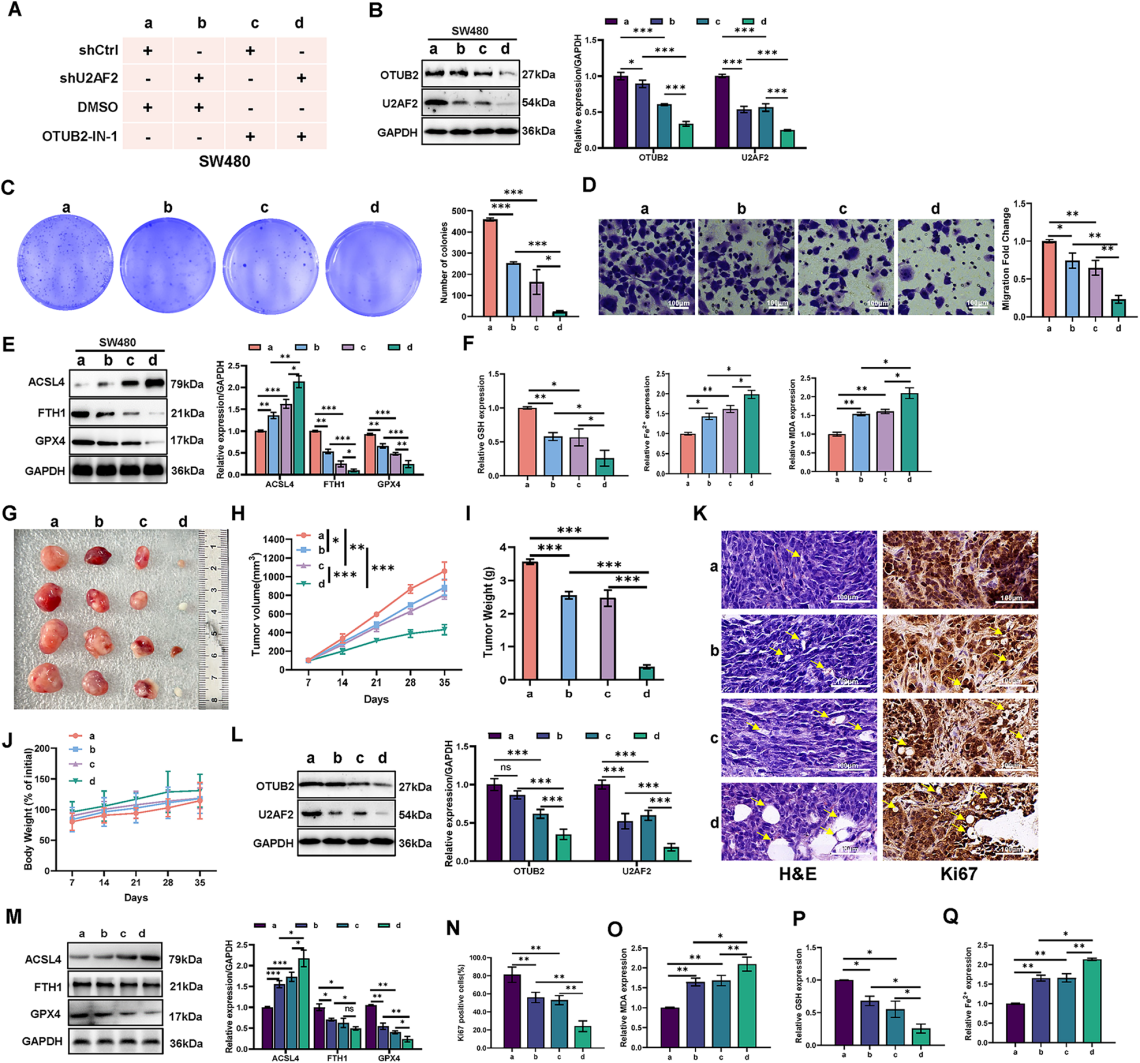
Combined inhibition of OTUB2 and U2AF2 synergistically suppresses CRC in vitro and in vivo. **A** SW480 cells were subjected to U2AF2 knockdown via siRNA transfection and treated with OTUB2-IN-1 separately. **B** WB analysis showing the expression levels of OTUB2 and U2AF2 proteins in cultured SW480 cells following U2AF2 knockdown and OTUB2-IN-1 treatment. **C** Effects of U2AF2 knockdown and OTUB2-IN-1 treatment on the proliferation of SW480 cells, assessed by the MTT assay. **D** Effects of U2AF2 knockdown and OTUB2-IN-1 treatment on the migration ability of SW480 cells, evaluated using a wound healing assay. Scale bar = 100 μm. **E** Expression levels of ferroptosis-related proteins in cultured SW480 cells after U2AF2 knockdown and OTUB2-IN-1 treatment. Representative WB images are shown (upper panel), with corresponding quantification presented as histograms (lower panel). **F** Levels of ferroptosis-related markers GSH, Fe²⁺, and MDA in cultured SW480 cells following U2AF2 knockdown and OTUB2-IN-1 treatment. **G** Representative images of tumors formed 35 days after subcutaneous inoculation of different cell lines into nude mice. **H** Tumor growth curves depicting volume changes over time in SW480 cell-derived tumors after U2AF2 knockdown and OTUB2-IN-1 treatment. **I** Average tumor weights of SW480 cell-derived tumors following U2AF2 knockdown and OTUB2-IN-1 treatment. **J** The body weight of nude mice during this experiment. **K** Representative images of H&E-stained tumor sections from SW480 cell-derived tumors after U2AF2 knockdown and OTUB2-IN-1 treatment. Scale bar = 100 μm. **L** Expression levels of OTUB2 and U2AF2 proteins in SW480 cell-derived tumors post U2AF2 knockdown and OTUB2-IN-1 treatment. **M** Expression levels of ferroptosis-related proteins in SW480 cell-derived tumors post U2AF2 knockdown and OTUB2-IN-1 treatment. Representative WB images are displayed (left panel), with quantification shown as histograms (right panel). **N** Ki-67 staining quantitative analysis. **O**–**Q** Levels of ferroptosis-related markers GSH, Fe²⁺, and MDA in SW480 cell-derived tumors after U2AF2 knockdown and OTUB2-IN-1 treatment. All experiments were performed in triplicate. Data are presented as mean ± standard deviation. Statistical significance was determined using one-way ANOVA followed by Tukey’s post hoc test. *p < 0.05, **p < 0.01, ***p < 0.001 compared to control groups.

In our previous experiments, we observed that knockdown of U2AF2 could negate the effects of OTUB2 overexpression. This prompted us to investigate whether the regulation of OTUB2 and U2AF2 could be synergistic when the OTUB2 and U2AF2 are regulated in the same direction. Therefore, we treated cells with the OTUB2 enzymatic inhibitor OTUB2-IN-1 alongside U2AF2 knockdown (Fig. 7A). WB assays confirmed the expression of OTUB2 and U2AF2 decreased after OTUB2-IN-1 treatment in U2AF2 knockdown cells (Fig. 7B). Colony formation and Transwell migration assays demonstrated that both U2AF2 knockdown and inhibition of OTUB2 enzymatic activity effectively suppressed the proliferation and migration of SW480 cells, and their effects were additive (Fig. 7C, D). Similar stepwise trends were observed in the levels of key ferroptosis-related proteins (Fig. 7E) and biochemical markers (Fig. 7F). Specifically, factors positively correlated with ferroptosis were inversely proportional to tumor proliferation and migration, while factors negatively correlated with ferroptosis were directly proportional. This indicates that OTUB2-U2AF2-dependent ferroptosis is negatively associated with tumor cell proliferation and invasion.

In vivo models confirmed that daily intraperitoneal injections of OTUB2-IN-1, starting 1 week after tumor cell inoculation in mice, significantly inhibited tumor growth, achieving effects similar to those observed with U2AF2 knockdown. When U2AF2-knockdown cells were further treated with OTUB2-IN-1, the tumor growth curve was the most attenuated (Fig. 7G), resulting in the smallest tumor volume and weight (Fig. 7H, I). And OTUB2-IN-1 treatment was not associated with any apparent toxicity, as there were no significant body weight changes observed between the four groups (Fig. 7J). Histopathological analysis revealed that tumor cells in the control group were uniform and dense with almost no vacuolization. U2AF2 knockdown led to sporadic vacuolization; OTUB2-IN-1 treatment resulted in clustered vacuolization; and combining U2AF2 knockdown with OTUB2-IN-1 treatment produced spongiform vacuolization and loose tumor tissue (Fig. 7K, N). These experiments confirm that enhancing the resistance of CRC tumor cells to ferroptosis is a key mechanism by which OTUB2 promotes tumorigenesis. Consistent with in vitro observations, the suppression of both OTUB2 and U2AF2 expression in tumor tissues was confirmed by WB upon dual intervention of U2AF2 knockdown and OTUB2-IN-1 treatment (Fig. 7L). The detection of key ferroptosis proteins (Fig. 7M) and biochemical markers (Fig. 7O–Q) in in vivo tumor tissue samples showed similar stepwise trends to those observed in vitro. These results suggest that dual regulation of the OTUB2-U2AF2 axis through genetic targeting and pharmacological methods can effectively inhibit tumors in CRC models in vivo, indicating that this strategy is a potential clinical therapeutic approach for CRC.

## Discussion

The present study elucidates the pivotal role of OTUB2-mediated deubiquitination in stabilizing U2AF2, which plays a critical function in promoting resistance to autophagy and ferroptosis in CRC cells. Our findings provide evidence that the disruption of the OTUB2-U2AF2 axis significantly enhances both ferroptosis and autophagy, leading to pronounced inhibition of tumor growth both in vitro and in vivo. These results not only deepen our understanding of how CRC cells evade cell death but also offer new therapeutic possibilities that may exploit these mechanisms to target tumor survival. By interfering with OTUB2’s ability to stabilize U2AF2, we uncover a dual vulnerability in CRC cells—autophagy and ferroptosis—that could be therapeutically exploited to enhance cancer cell death.

Ubiquitination and deubiquitination are essential for the maintenance of protein homeostasis, which is a fundamental process in all cells, but particularly in cancer cells, where proteostasis is often dysregulated to support uncontrolled growth and survival [39, 40]. OTUB2, as a deubiquitinating enzyme, stabilizes U2AF2, a splicing factor that plays an important role in RNA processing, especially in alternative splicing events that can drive oncogenesis [8]. Alternative splicing allows cancer cells to adapt to various stressors, including chemotherapeutic agents and nutrient deprivation, by generating protein isoforms that promote survival. The stabilization of U2AF2 by OTUB2 contributes to the survival of CRC cells under such stress conditions. Our study supports previous research, which suggests that dysregulation of the ubiquitin-proteasome system in cancer leads to resistance against not only apoptosis but also other forms of programmed cell death [41–44]. Ferroptosis, an iron-dependent form of programmed cell death, has recently garnered significant attention as a promising target for cancer therapy. This process has been shown to play a critical role in overcoming resistance in cancer cells that have become unresponsive to traditional therapies [45]. In our study, we observed that knocking down OTUB2 in CRC cells resulted in increased lipid peroxidation and a reduction in antioxidant defenses, thereby enhancing ferroptosis. This aligns with the growing body of literature that suggests ferroptosis and autophagy are closely linked processes, often regulated in parallel or even co-activated within the tumor microenvironment [46–48].

Moreover, the relationship between autophagy and ferroptosis in cancer cells is complex and context-dependent [34]. Autophagy is a cellular degradation process that recycles damaged or unnecessary cellular components, allowing cells to survive under nutrient deprivation and other stress conditions. While autophagy has been traditionally viewed as a survival mechanism, it can also act as a tumor suppressor by clearing damaged mitochondria and other cellular debris that could otherwise lead to uncontrolled proliferation. However, in the context of CRC, our results show that OTUB2 not only inhibits ferroptosis but also suppresses autophagy, creating a dual layer of protection for cancer cells against death. Upon OTUB2 knockdown, we observed a significant increase in autophagosome formation, along with enhanced mitochondrial damage and ROS accumulation, indicating that OTUB2 actively suppresses autophagy to prevent ferroptosis in CRC cells. Despite the progress made in understanding the role of OTUB2 and U2AF2 in regulating CRC cell survival, several key questions remain unanswered. For instance, the precise mechanisms through which U2AF2 stabilizes key components of both the ferroptosis and autophagy pathways remain unclear. Additionally, the broader implications of targeting the OTUB2-U2AF2 axis in other cancers, as well as in normal tissues, remain to be explored. Since ubiquitination and deubiquitination are fundamental to a wide range of cellular processes, it is important to consider the potential off-target effects that may arise from inhibiting OTUB2, particularly in normal cells where these pathways are equally essential for homeostasis.

While our in vivo studies showed no significant weight loss in treated mice, comprehensive toxicology assessments-particularly regarding normal intestinal crypt cells and systemic iron homeostasis-are essential. Encouragingly, OTUB2 inhibitors (e.g., OTUB2-IN-1) exhibit cancer cell selectivity in preclinical models [9], suggesting a viable therapeutic window. Furthermore, the gut microbiome is a key modulator of CRC progression and therapy response. Though not directly examined here, microbiota-derived metabolites may influence ferroptosis sensitivity [49] or regulate proteasomal degradation pathways relevant to CRC growth [50]. Future studies should investigate whether microbiome composition impacts the OTUB2-U2AF2 axis or therapeutic efficacy of its inhibitors.

Future research should focus on exploring the potential of combining ferroptosis inducers with OTUB2 inhibitors as a therapeutic strategy. By targeting both the ferroptosis and autophagy pathways simultaneously, it may be possible to create a synergistic effect that overcomes the resistance mechanisms employed by CRC cells. Developing OTUB2-specific inhibitors or U2AF2 degraders-potentially leveraging PROTAC (Proteolysis-Targeting Chimera) technology for targeted protein degradation-could benefit CRC patients with high OTUB2 expression. Additionally, investigating the role of other deubiquitinating enzymes that may also influence the ferroptosis-autophagy axis could provide a more comprehensive understanding of how these processes are regulated in cancer cells. Expanding this research to include clinical trials would also be critical to determining the safety and efficacy of targeting the OTUB2-U2AF2 axis in CRC and potentially other cancers. Future efforts should prioritize pharmacokinetic optimization and validation in patient-derived organoids/PDX models to accelerate clinical translation.

**Fig. 8:**
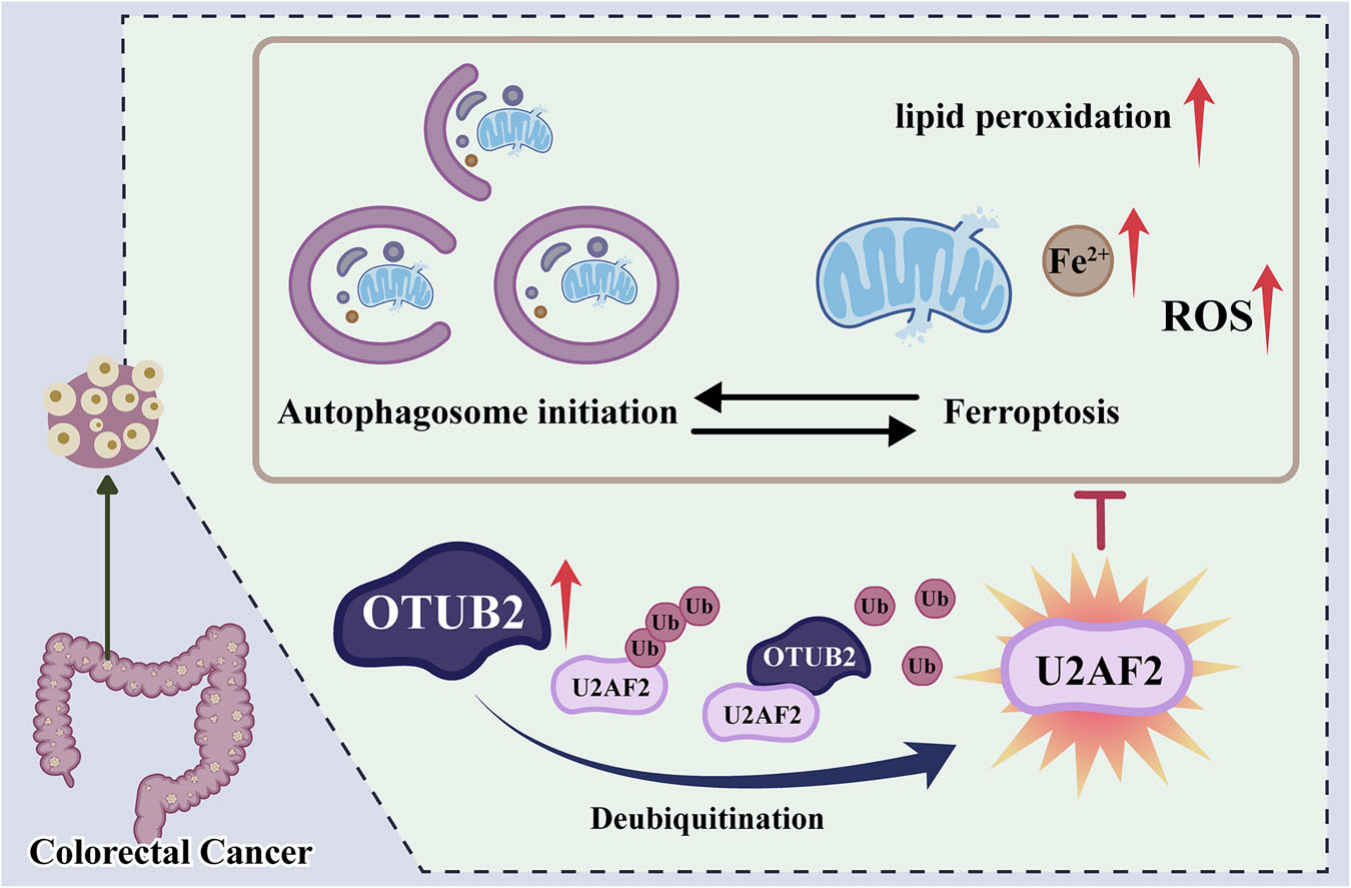
Schematic of the OTUB2-U2AF2 axis in the CRC model that targets autophagy-ferroptosis. OTUB2 functions as a deubiquitinase, exhibiting deubiquitinating activity through its interaction with U2AF2. Regulation of the OTUB2-U2AF2 axis, achieved by knocking down OTUB2 or U2AF2, can activate autophagy-coupled ferroptosis in CRC cells.

In conclusion, this study demonstrates that the OTUB2-U2AF2 axis plays a critical role in regulating autophagy-ferroptosis in CRC cells (Fig. 8). By disrupting this axis, we have shown that cancer cells become significantly more sensitive to ferroptosis, leading to impaired tumor growth. These findings not only contribute to the growing body of research on the regulation of ferroptosis in cancer therapy but also open up new avenues for developing more effective treatments for CRC. Targeting the balance between ubiquitination and deubiquitination, particularly in the context of ferroptosis, may hold great promise for improving outcomes in patients with CRC and potentially other cancers characterized by resistance to cell death.

## Supplementary information

Supplementary Figures legends

Reproducibility checklist

Uncropped Blots

Supplementary Figure S1

Supplementary Figure S2

## Data Availability

The data analyzed during the current study are available from the corresponding author on reasonable request.
