# Supplementary material for: OTUB2-mediated deubiquitination upregulates U2AF2 to promote colorectal cancer evasion of autophagy-ferroptosis
Source: Cell Death Dis. 2026 May 7;17(1):607. doi: 10.1038/s41419-026-08415-8 (PMC13319764; doi:10.1038/s41419-026-08415-8)
Supplement: Supplementary file 1 — Supplementary Figures legends [file 41419_2026_8415_MOESM1_ESM.docx]

**Supplementary Figure legends**

| **Figure S1. Differentially expressed genes in CRC.** |
| --- |
| (A) Hierarchical clustering heatmap of gene expression profiles of differentially expressed genes (DEGs) between adjacent normal mucosa (blue) and primary CRC tissues (red), with \|LogFC\| ≥ 0 and p-value < 0.05. (B) Volcano plot showing fold changes (LogFC) and p-values from significance tests for DEGs between primary CRC tissues and adjacent normal mucosa, highlighting significant differences between the two groups. (C) Hierarchical clustering heatmap of gene expression profiles of DEGs between colorectal liver metastases (blue) and primary CRC tissues (red), with \|LogFC\| ≥ 0 and p-value < 0.05. (D) Volcano plot showing fold changes (LogFC) and p-values from significance tests for DEGs between colorectal liver metastases and primary CRC tissues, highlighting significant differences between the two groups. (E) Venn diagram illustrating the intersection of DEGs between adjacent normal mucosa and primary CRC tissues, and DEGs between colorectal liver metastases and primary CRC tissues. Numbers represent the quantity of DEGs, and percentages indicate their proportion in the total number of genes. (F) Enrichment analysis of DEGs based on the Kyoto Encyclopedia of Genes and Genomes (KEGG) and Gene Ontology (GO). GO functions include Biological Processes (BP), Cellular Components (CC), and Molecular Functions (MF). |
| **Figure S2. Overexpression of OTUB2 increases CRC cell viability and confers resistance to ferroptosis.** |
| (A) WB results demonstrating the overexpression of OTUB2 in LoVo and RKO cell lines using plasmids containing the full-length OTUB2 sequence. (B) Colony formation assays demonstrating the effect of OTUB2 overexpression on the proliferative capacity of LoVo and RKO cells. (C) Transwell assays showing the impact of OTUB2 overexpression on the migration ability of LoVo and RKO cells. Scale bar = 100 μm. (D) PI staining and flow cytometry analyses indicating the effect of OTUB2 overexpression on cell death in LoVo and RKO cells. (E) Endothelial adhesion assays demonstrating the effect of OTUB2 overexpression on the metastatic adhesion capability of LoVo and RKO cells. Scale bar = 100 μm. (F) WB analyses showing the impact of OTUB2 overexpression on the expression levels of epithelial-mesenchymal transition-related proteins E-Cadherin, N-Cadherin, and Vimentin in LoVo and RKO cells. (G) Effects of OTUB2 overexpression on ferroptosis-related biochemical indicators GSH, Fe²⁺, and MDA in LoVo and RKO cells. (H) Impact of OTUB2 overexpression on the expression of ferroptosis-related proteins ACSL4, FTH1, and GPX4 in LoVo and RKO cells. (I) BODIPY C11 probe staining showing intracellular ROS levels in LoVo and RKO cells after OTUB2 overexpression. Scale bar = 100 μm. (J) CCK-8 assays indicating cell viability of LoVo and RKO cells treated with different concentrations of Erastin following OTUB2 overexpression.  All experiments were performed in triplicate. Data are presented as mean ± standard deviation. Statistical significance was determined using one-way ANOVA followed by Tukey's post hoc test. *P < 0.05, **P < 0.01, ***P < 0.001 compared to control groups. |
