# Supplementary figures and images for: OTUB2-mediated deubiquitination upregulates U2AF2 to promote colorectal cancer evasion of autophagy-ferroptosis
Source: Cell Death Dis. 2026 May 7;17(1):607. doi: 10.1038/s41419-026-08415-8 (PMC13319764; doi:10.1038/s41419-026-08415-8)

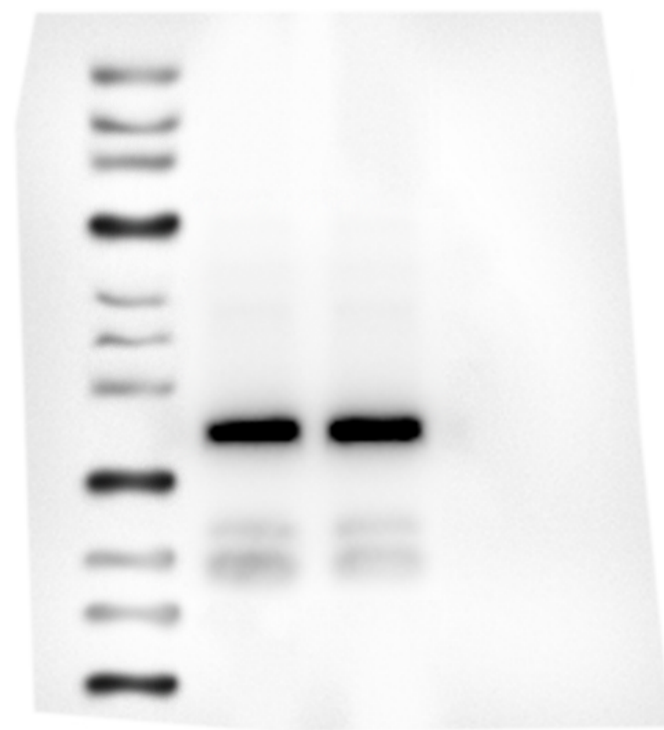

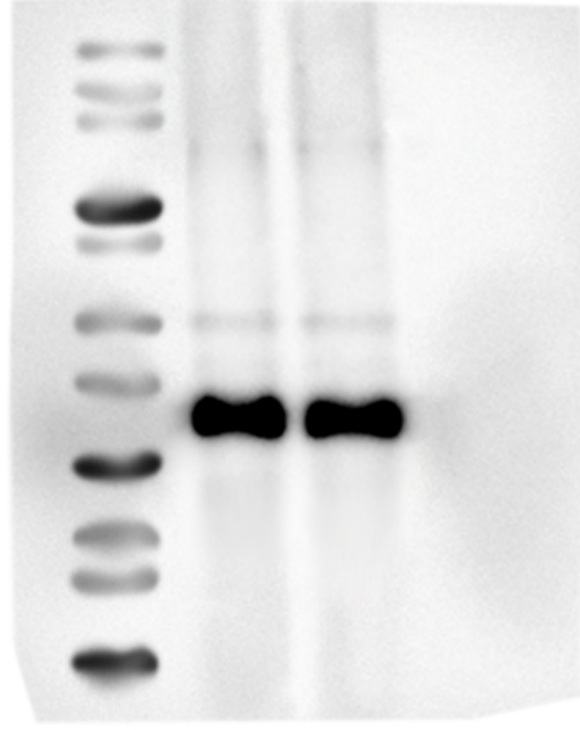

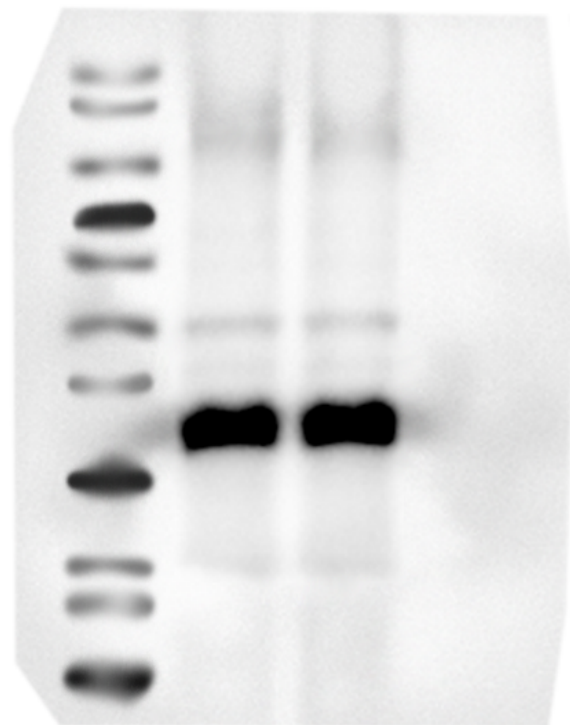

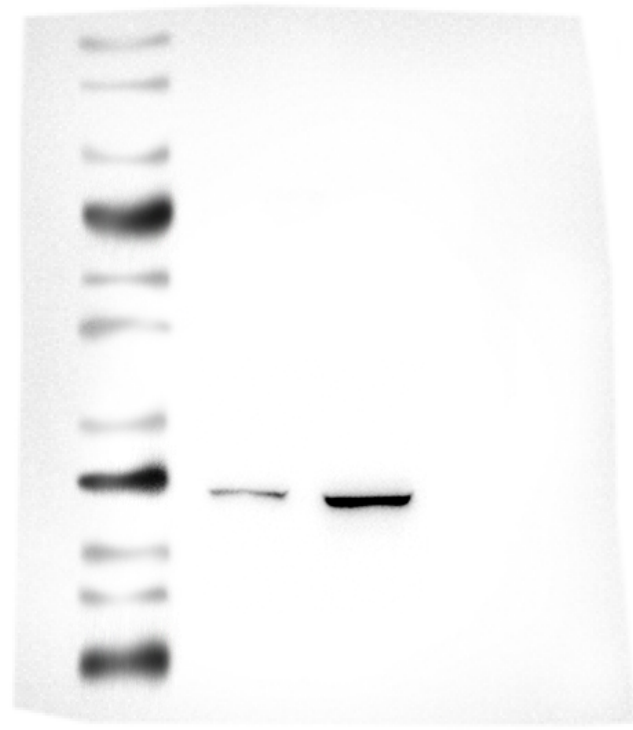

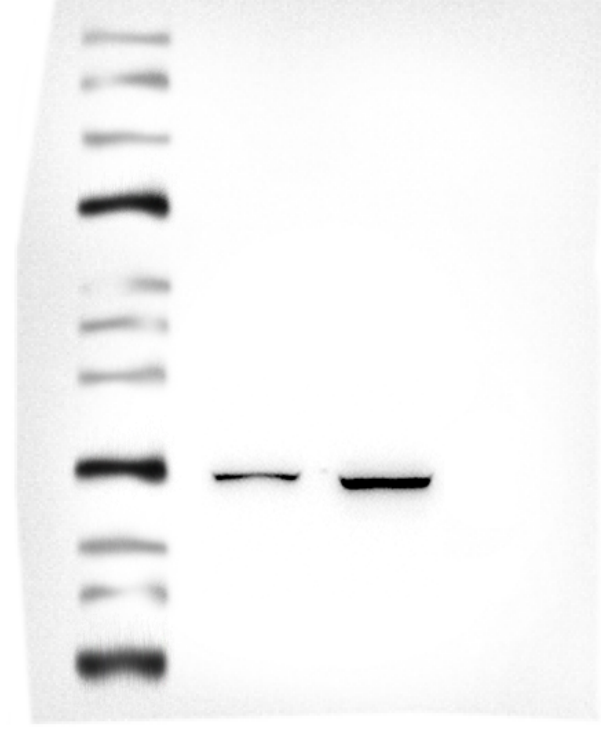

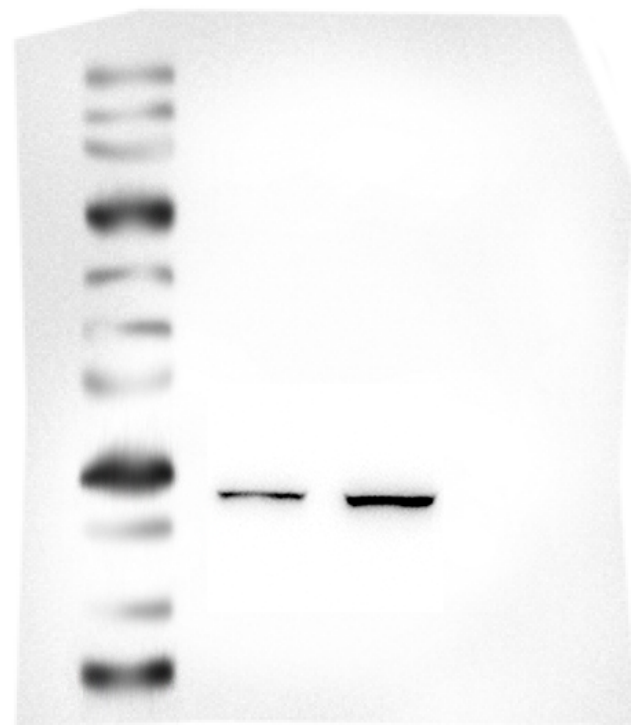

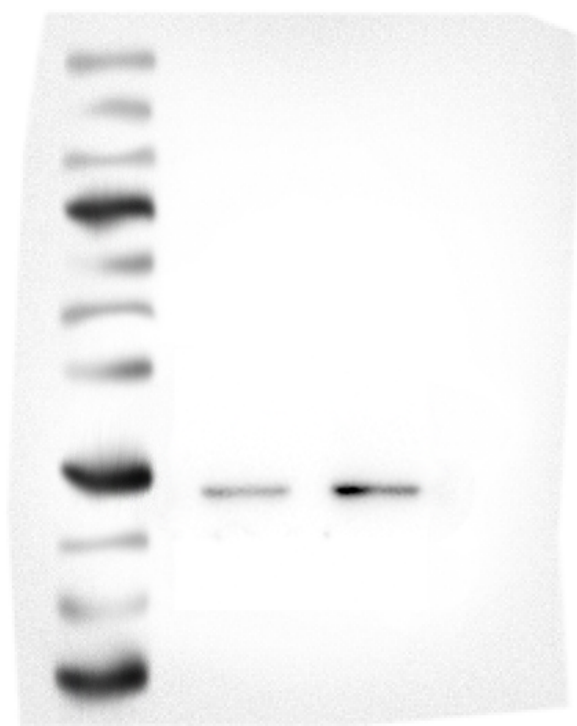

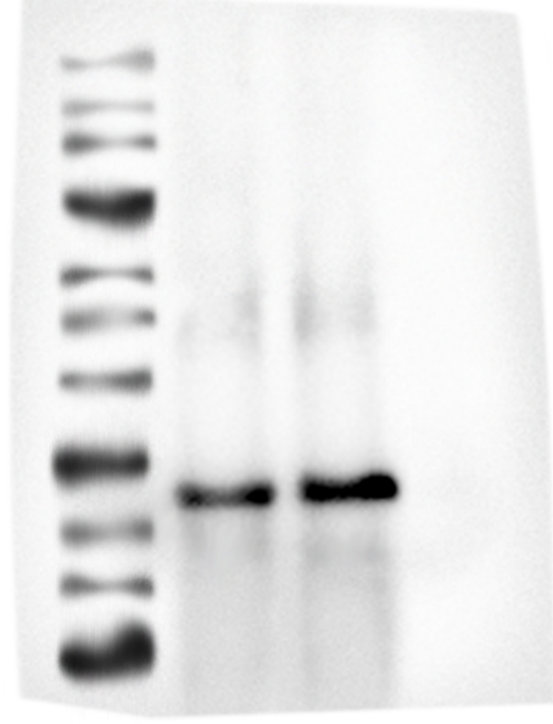

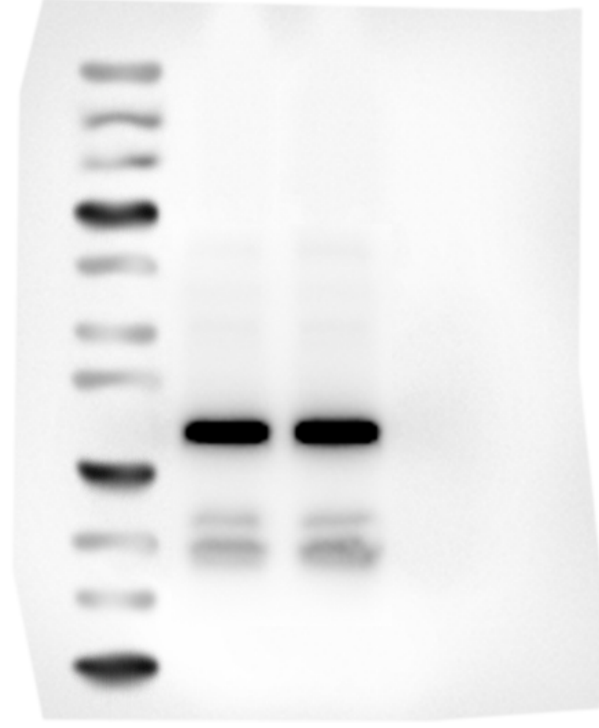

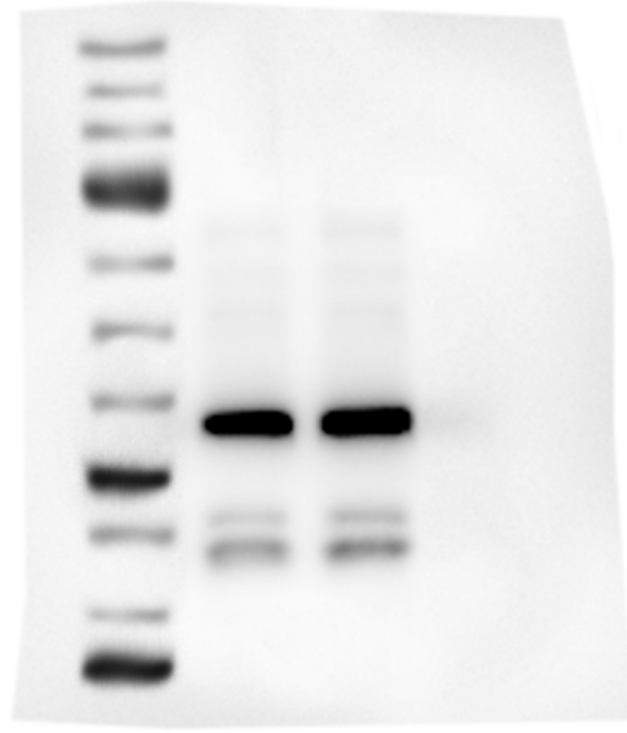

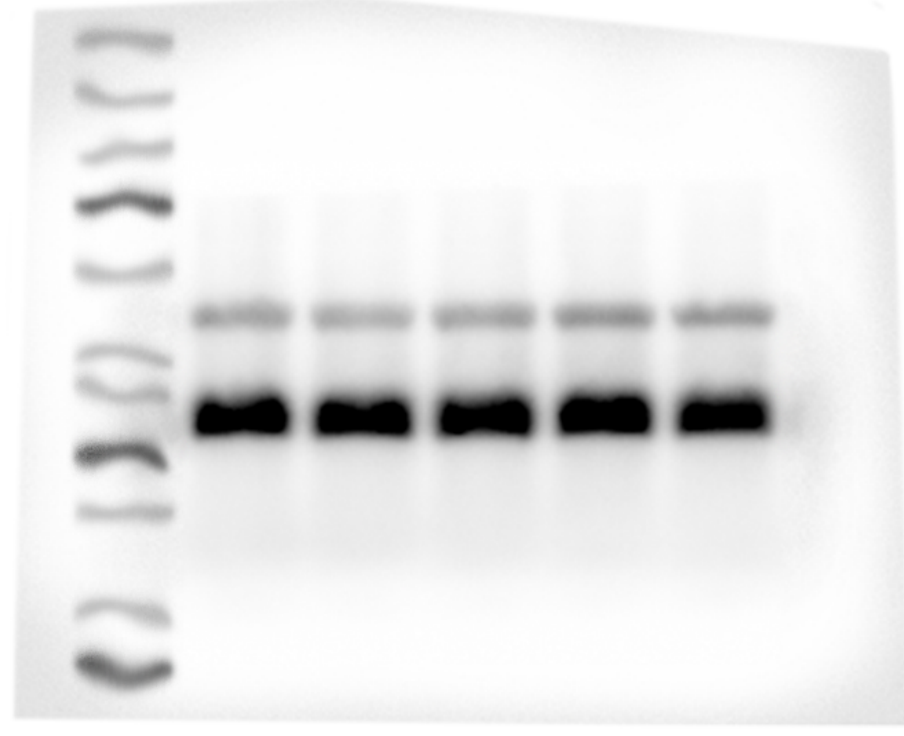

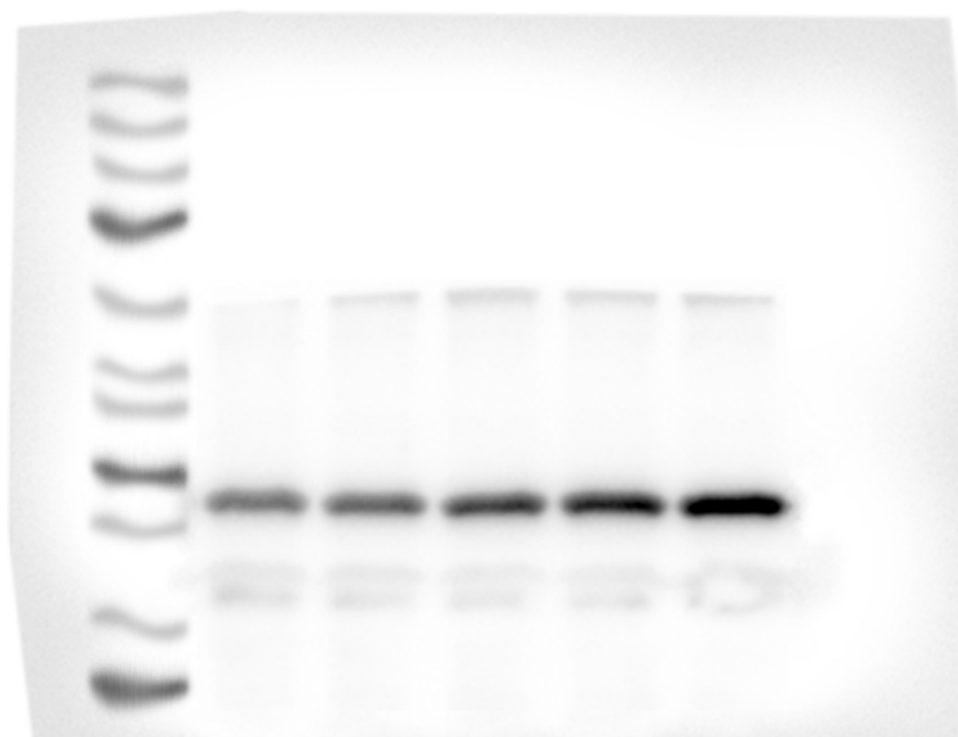

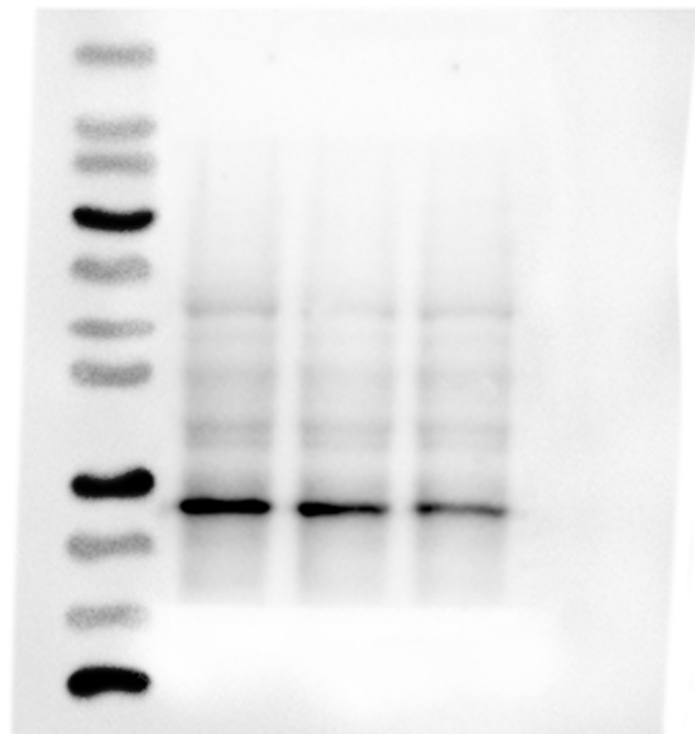

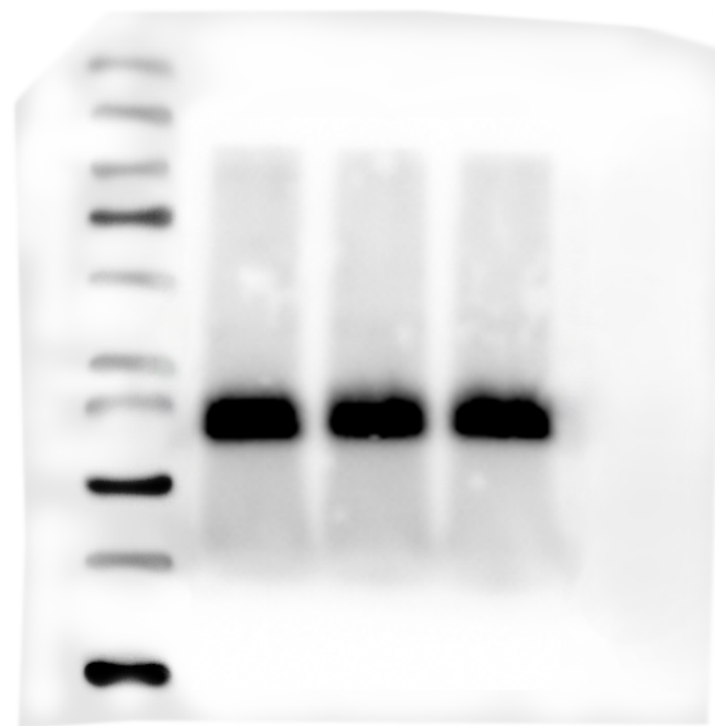

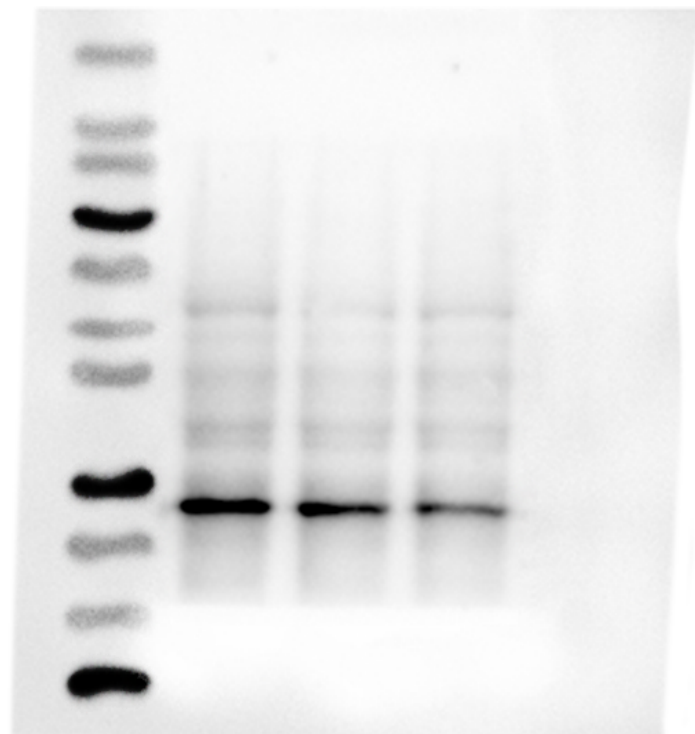

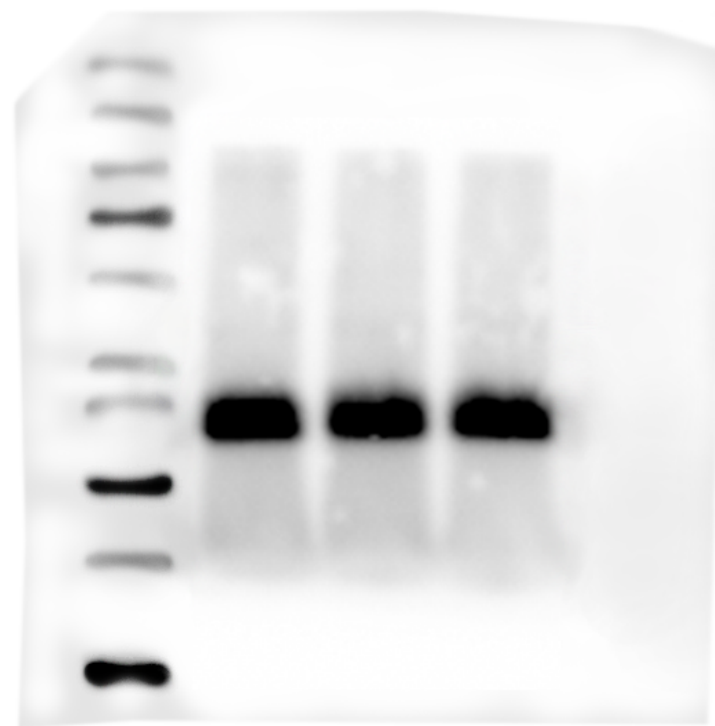

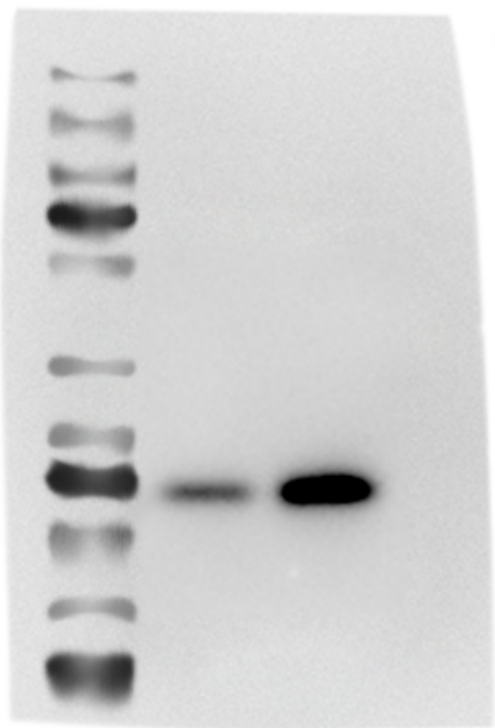

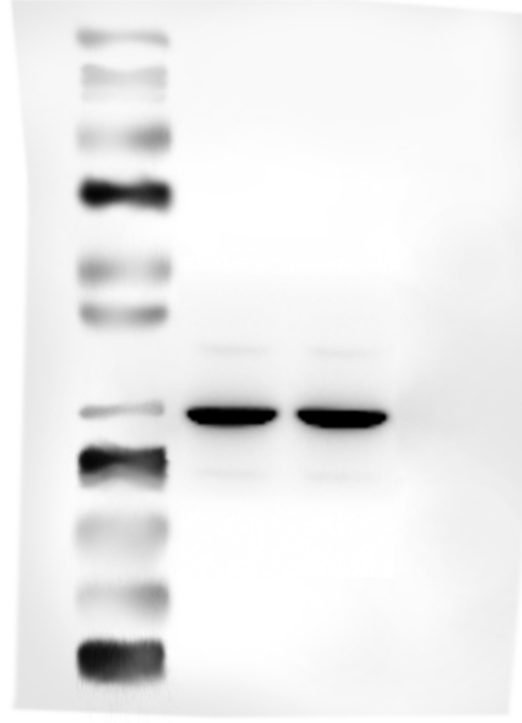

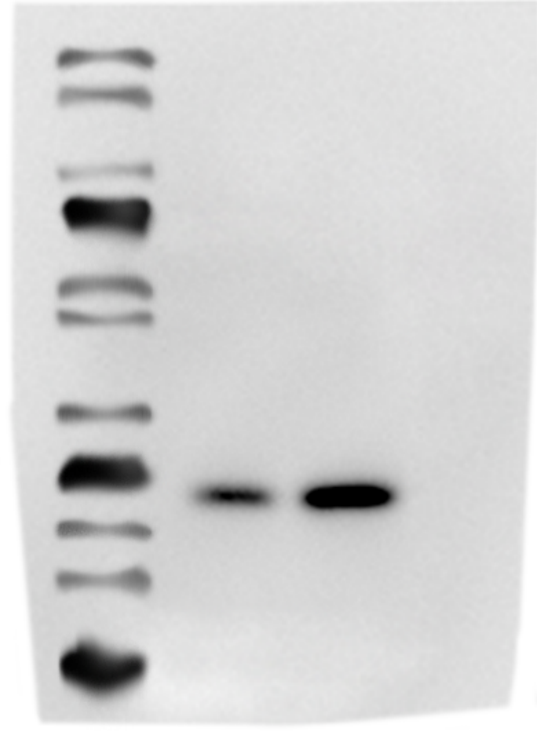

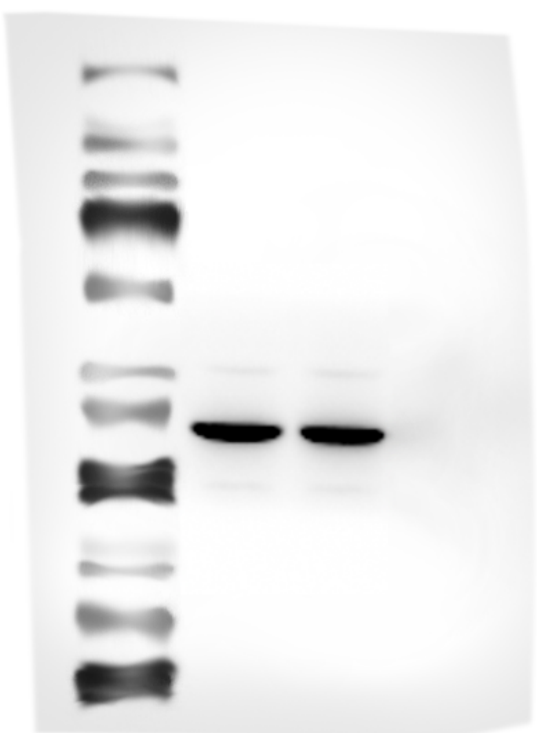

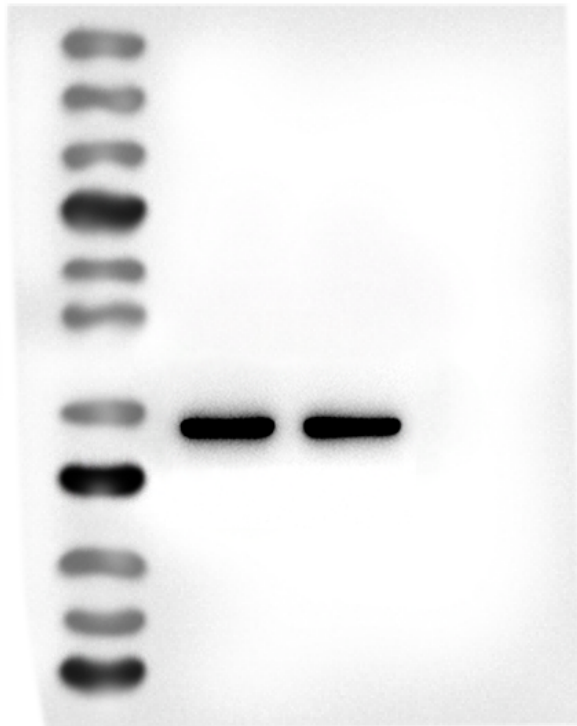

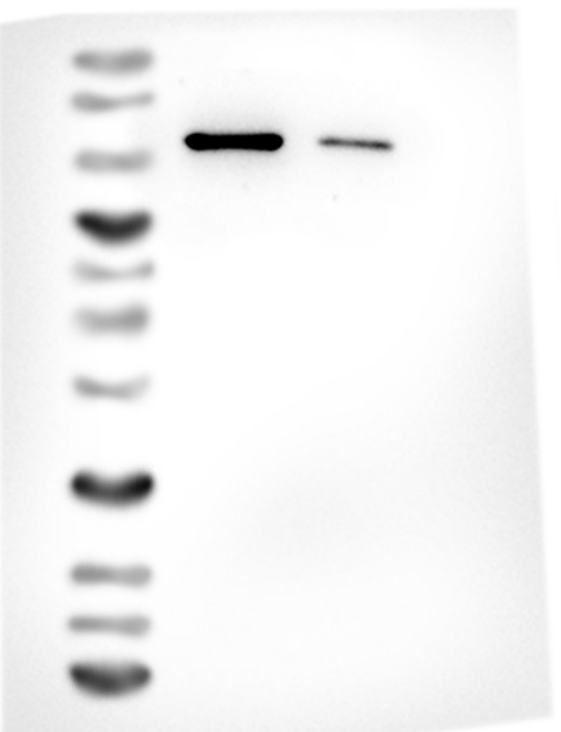

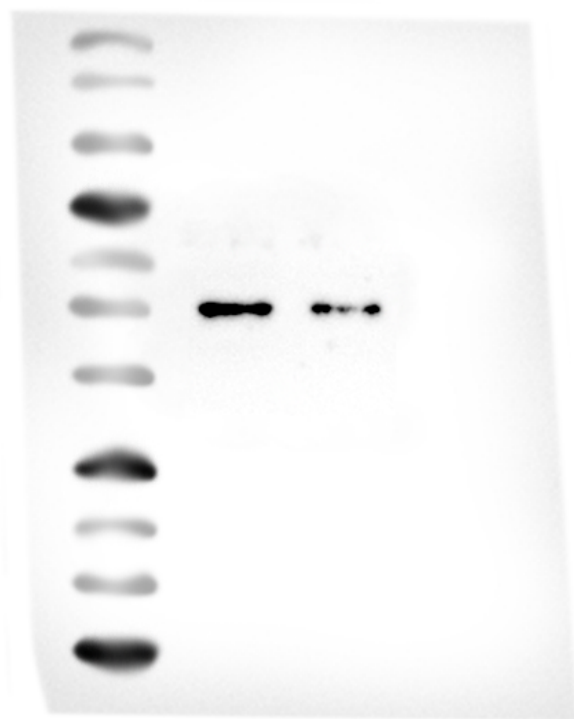

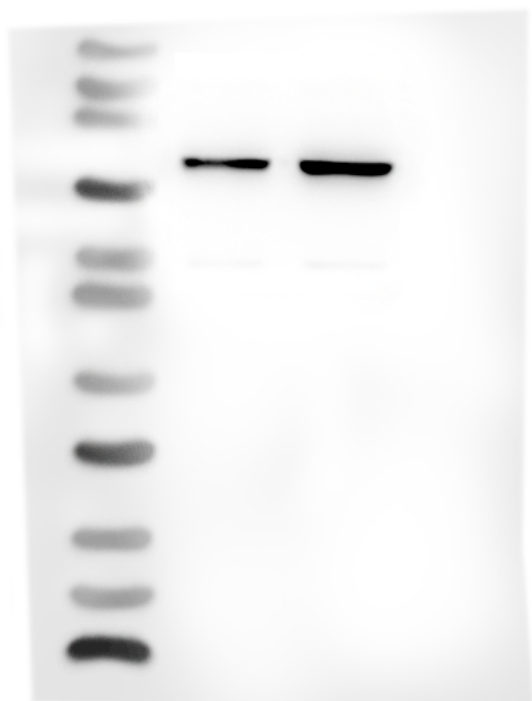

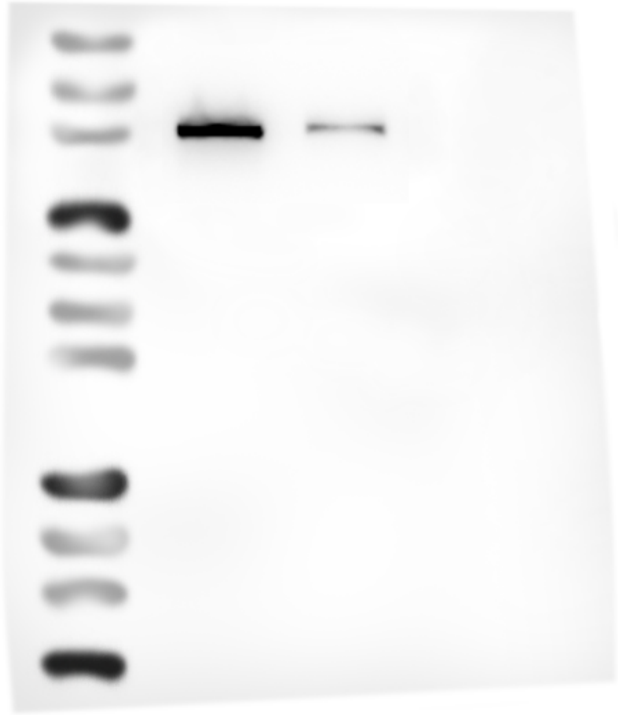

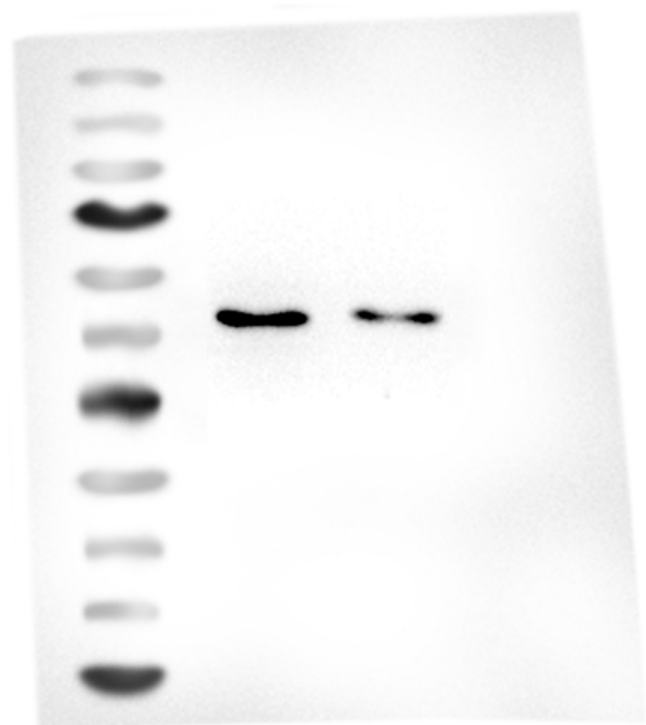

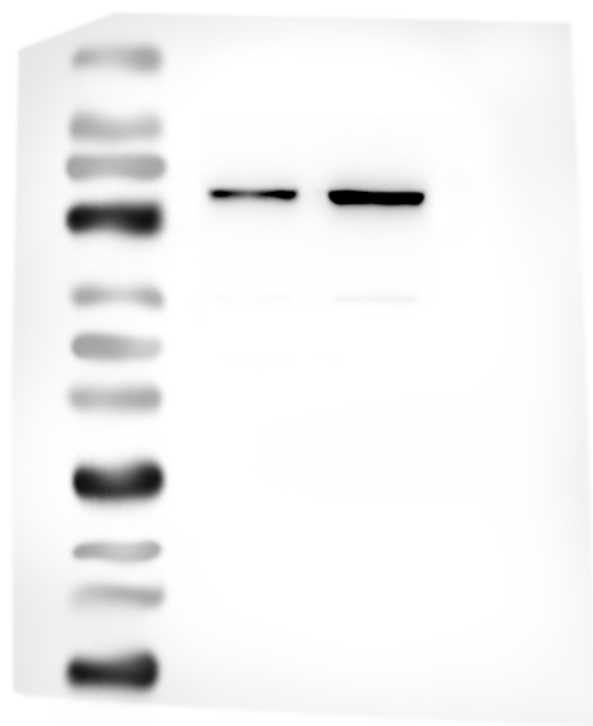

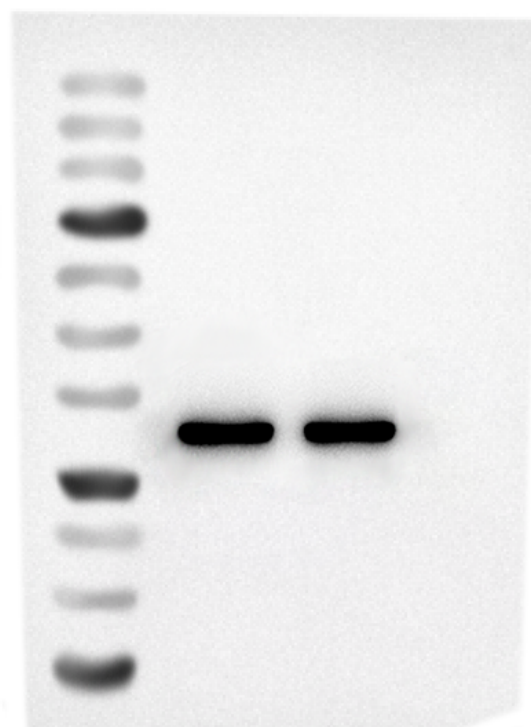

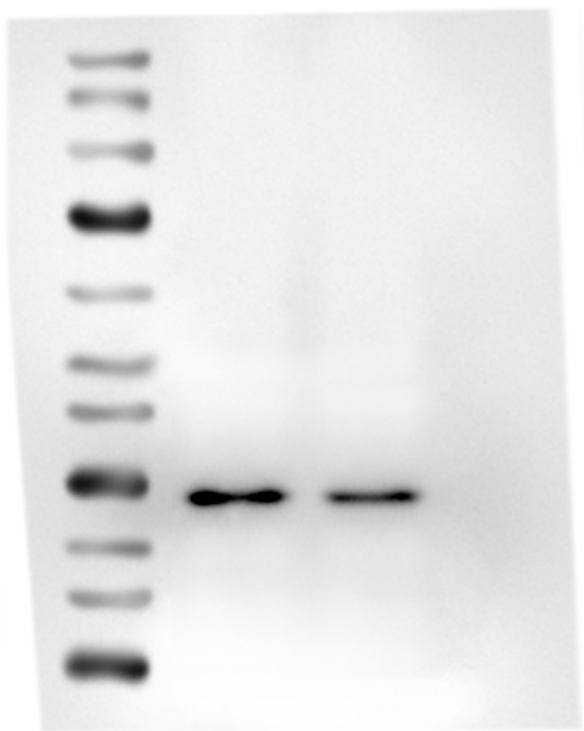

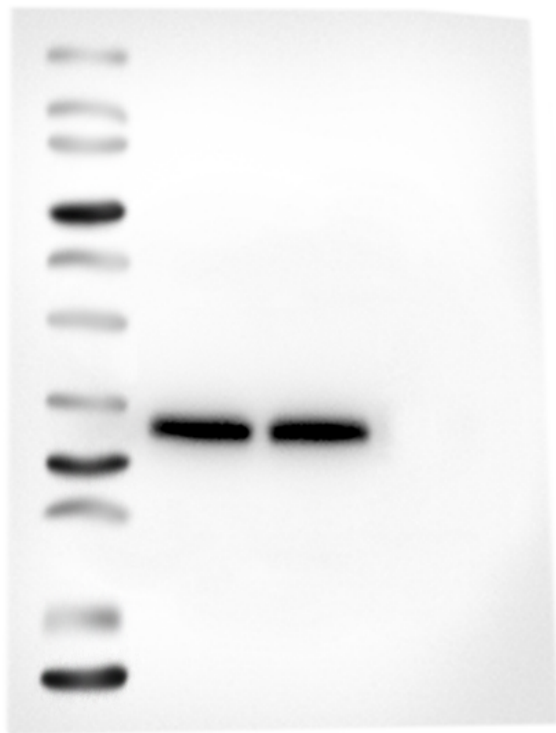

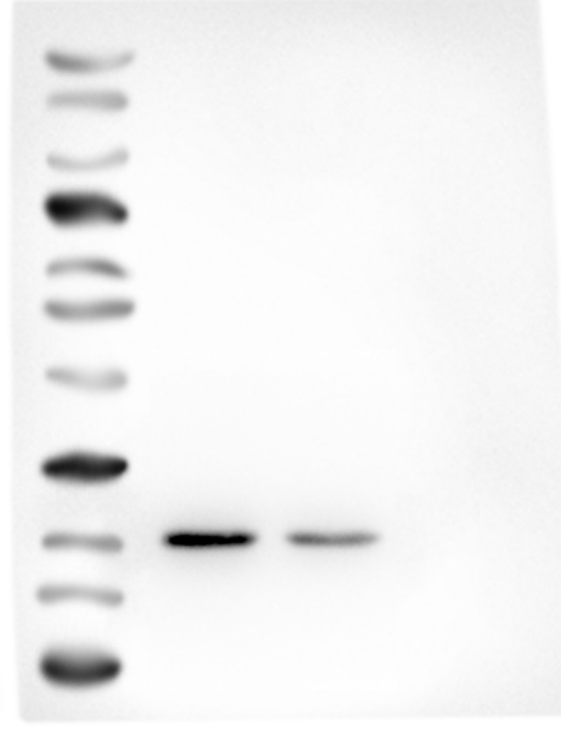

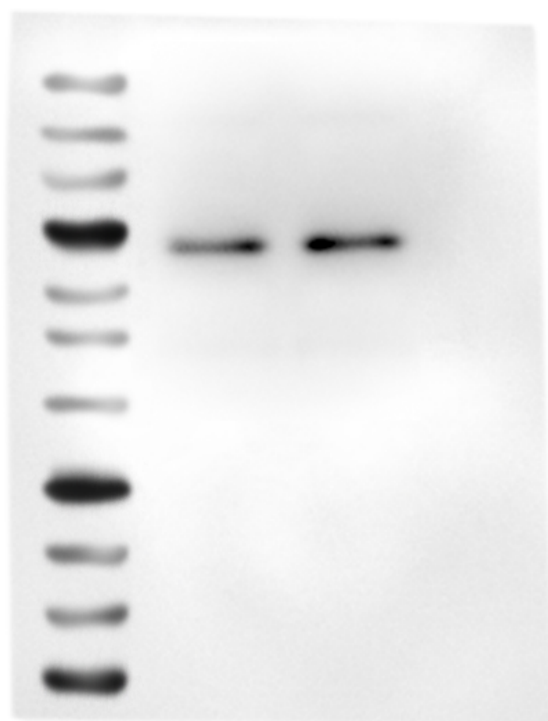

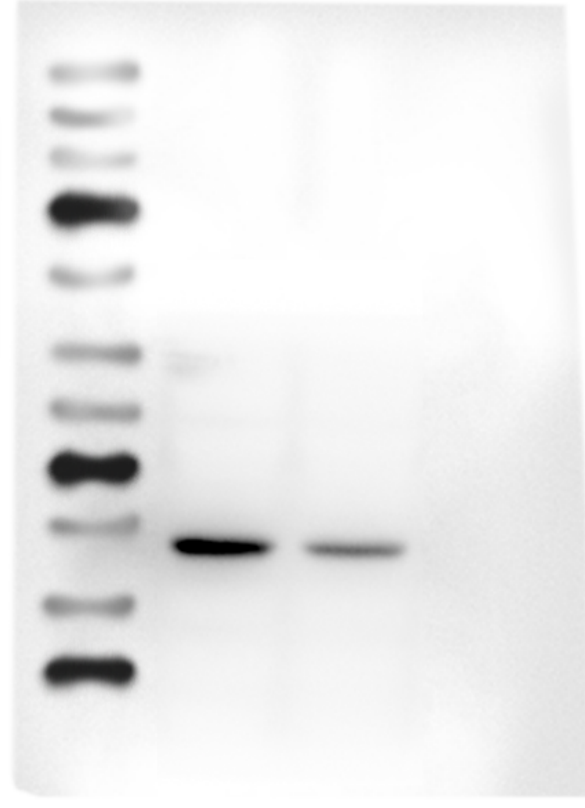

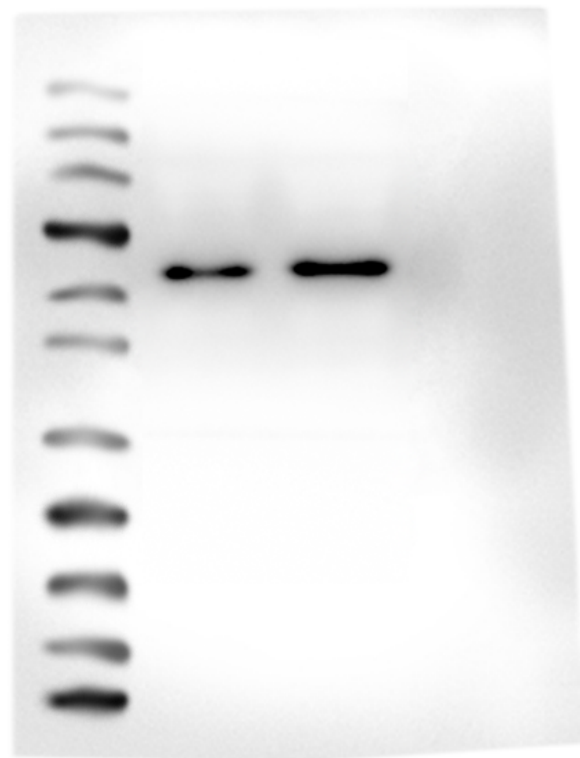

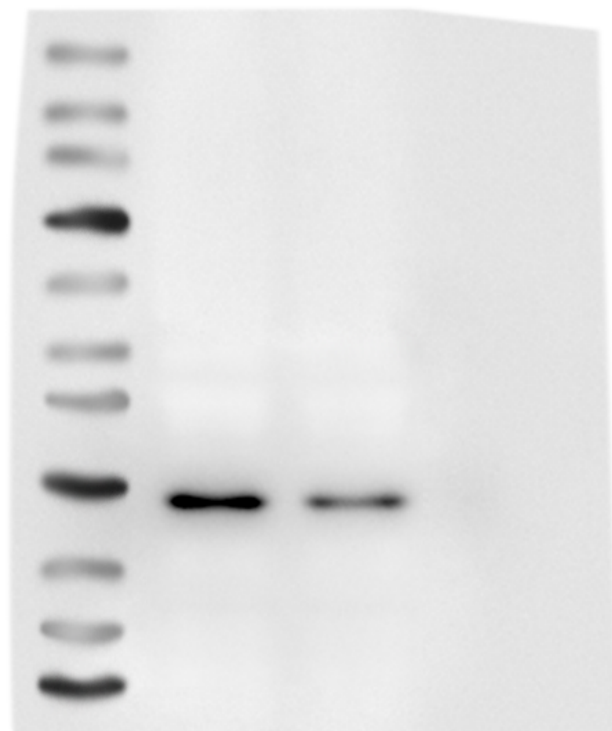

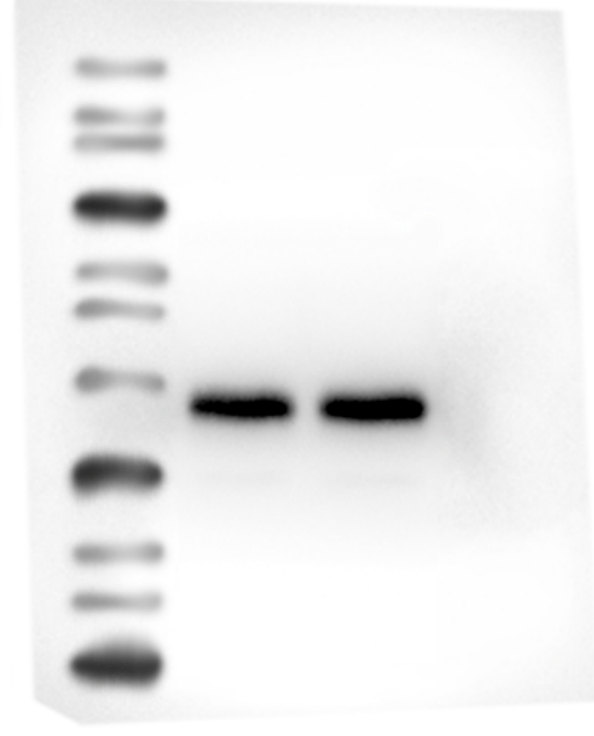

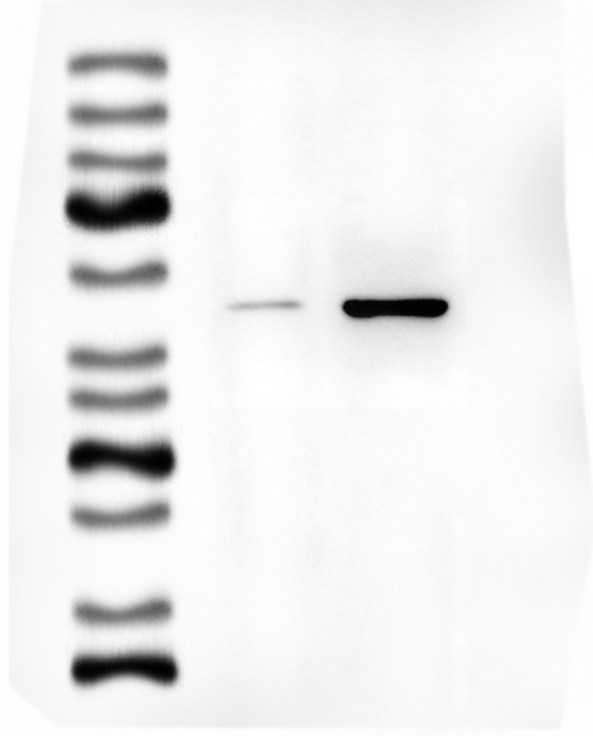

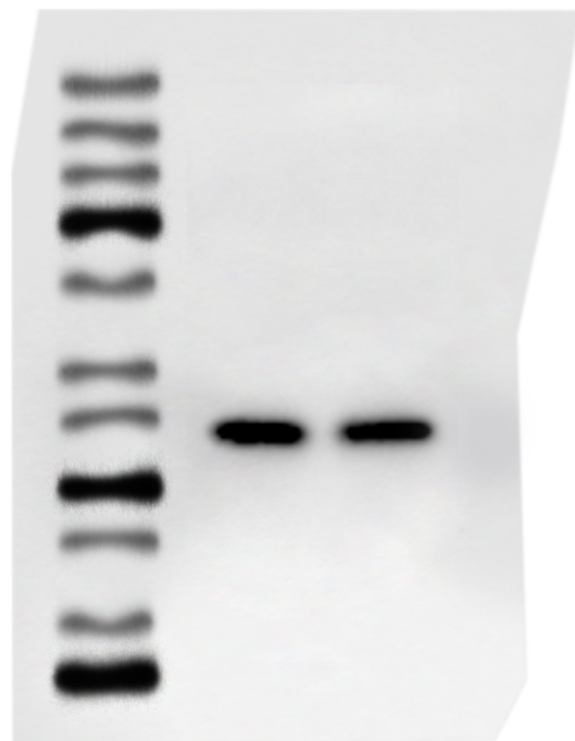

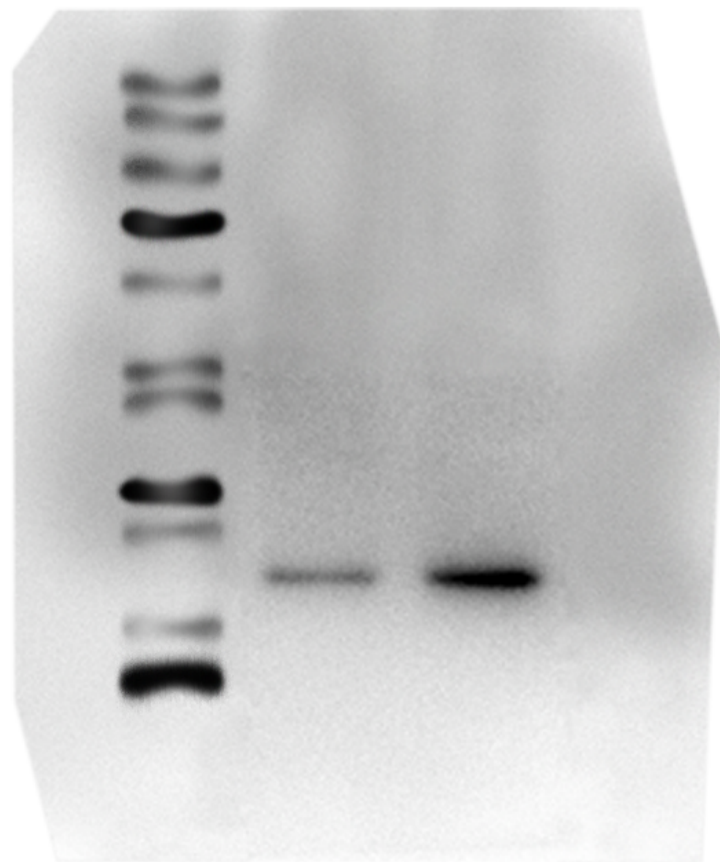

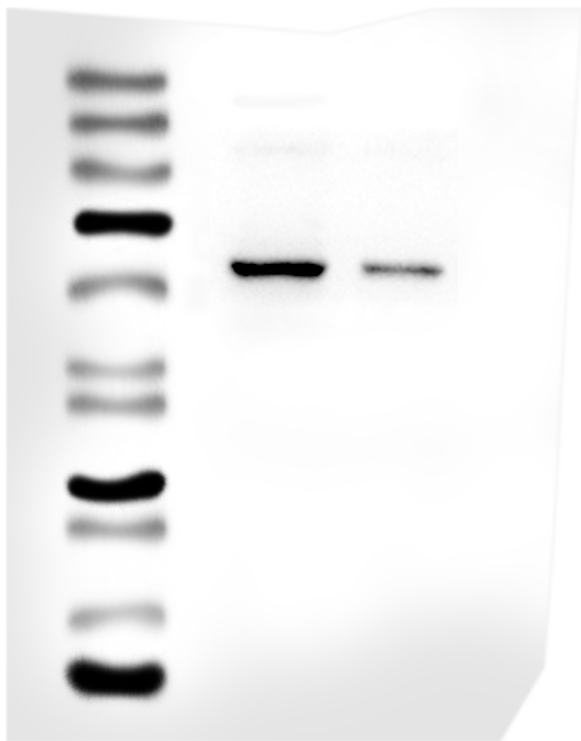

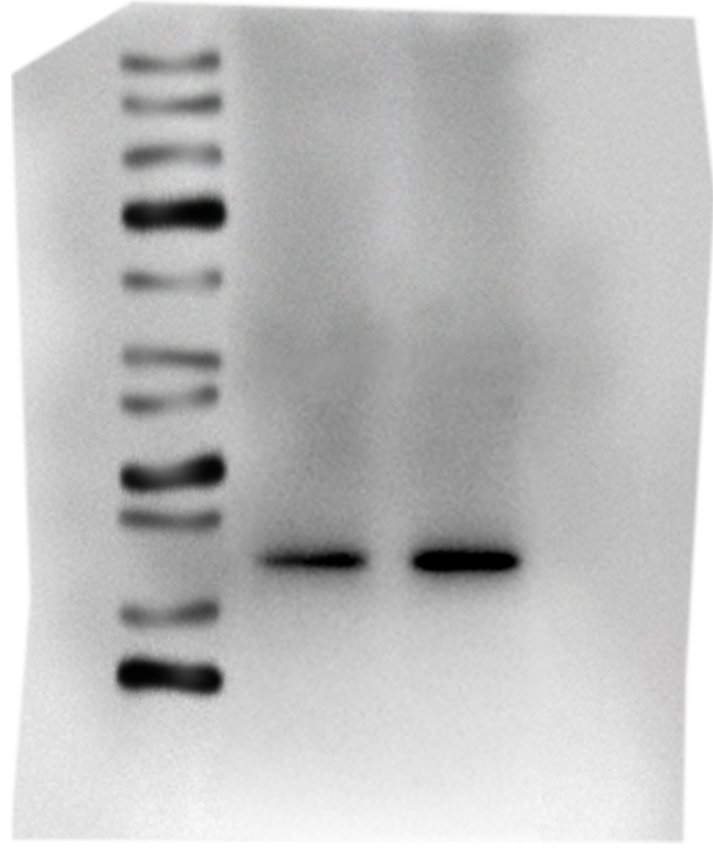

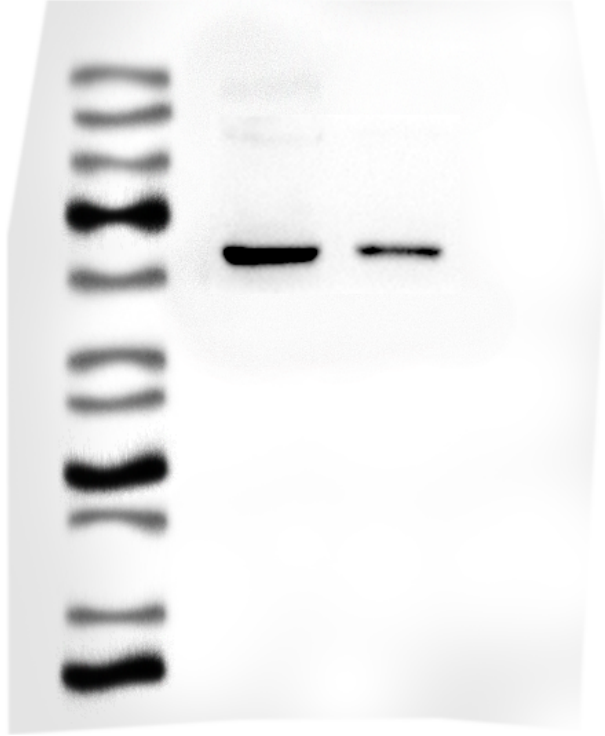

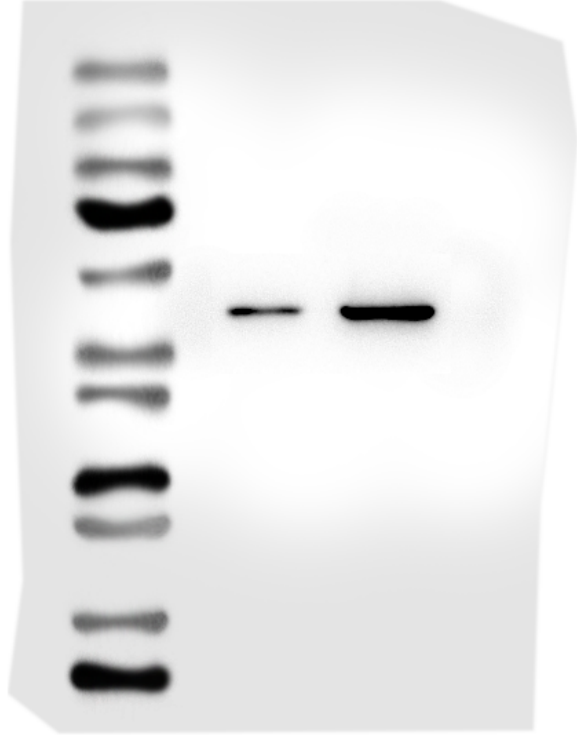

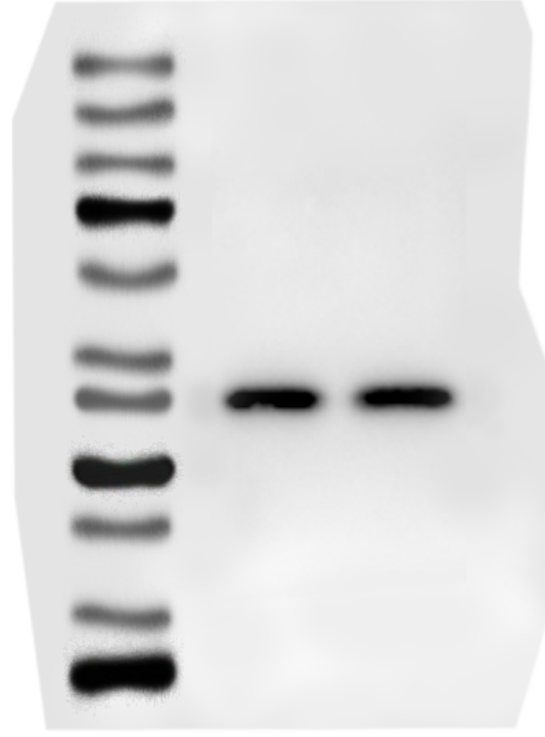

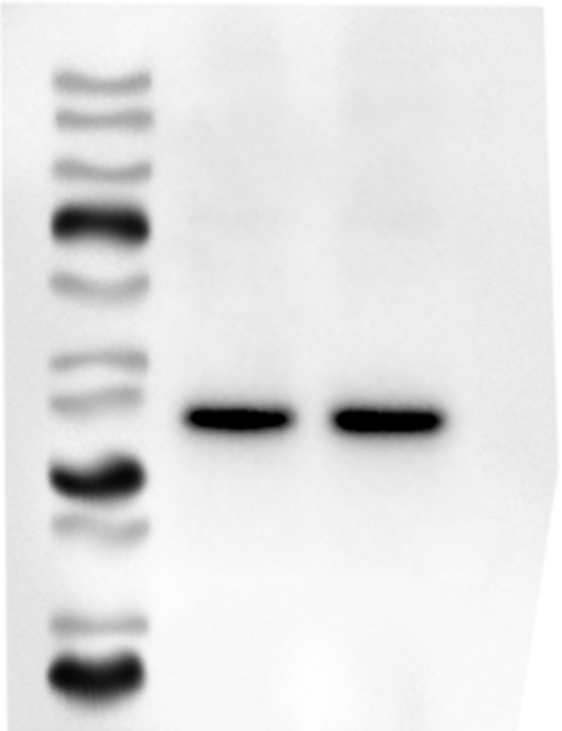

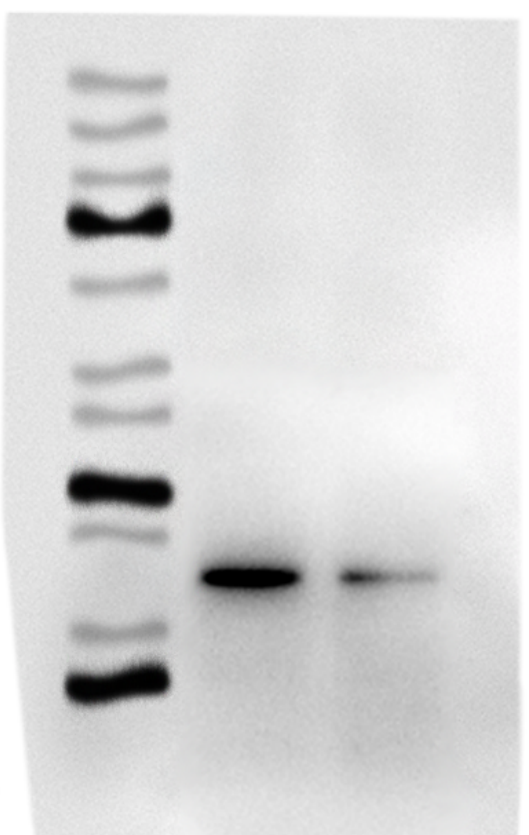

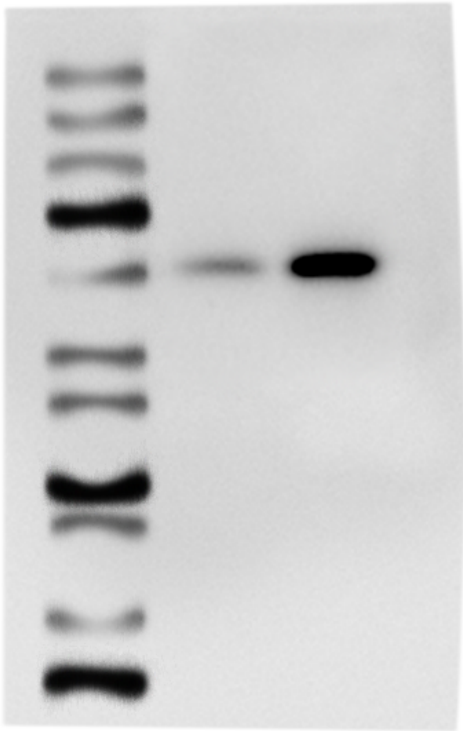

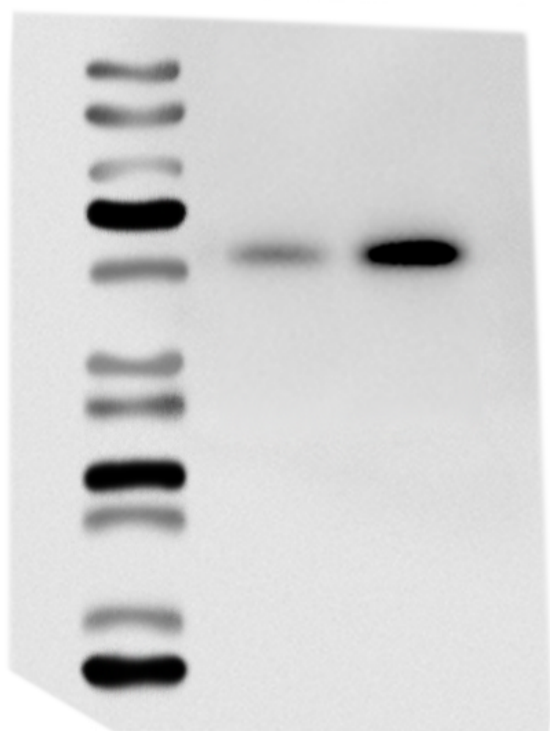

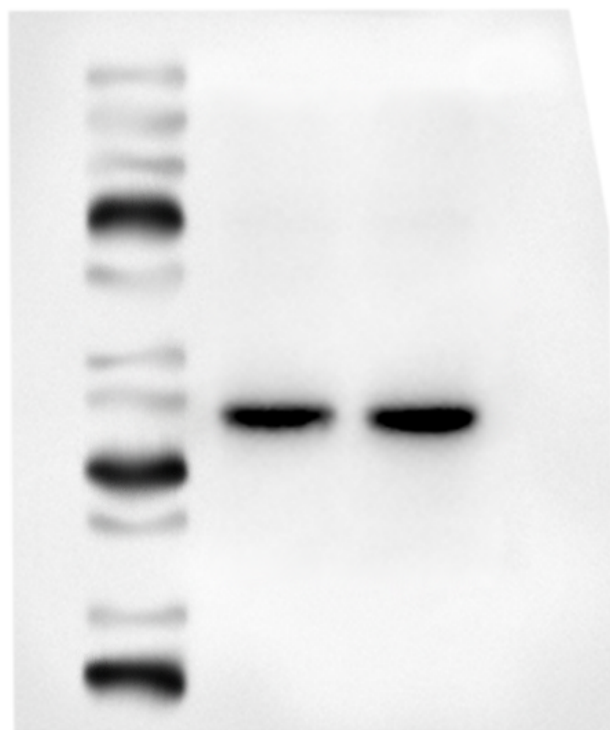

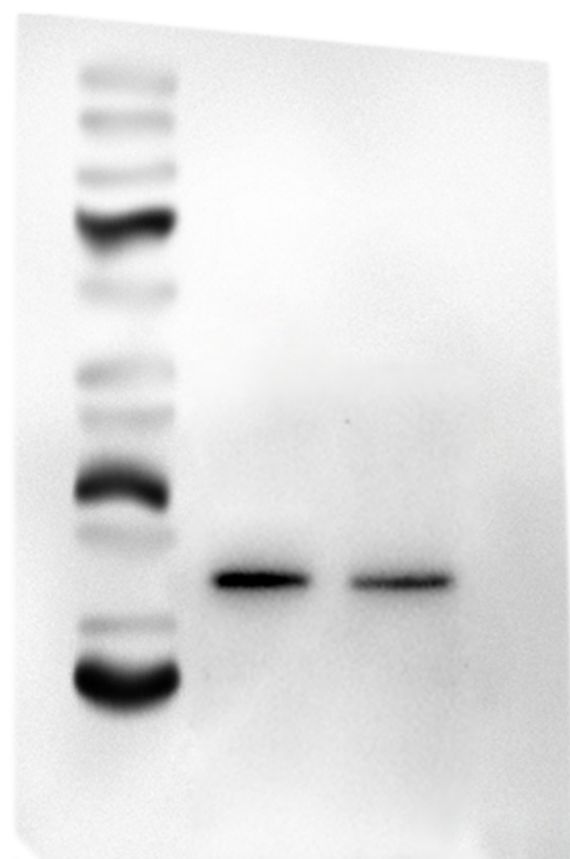

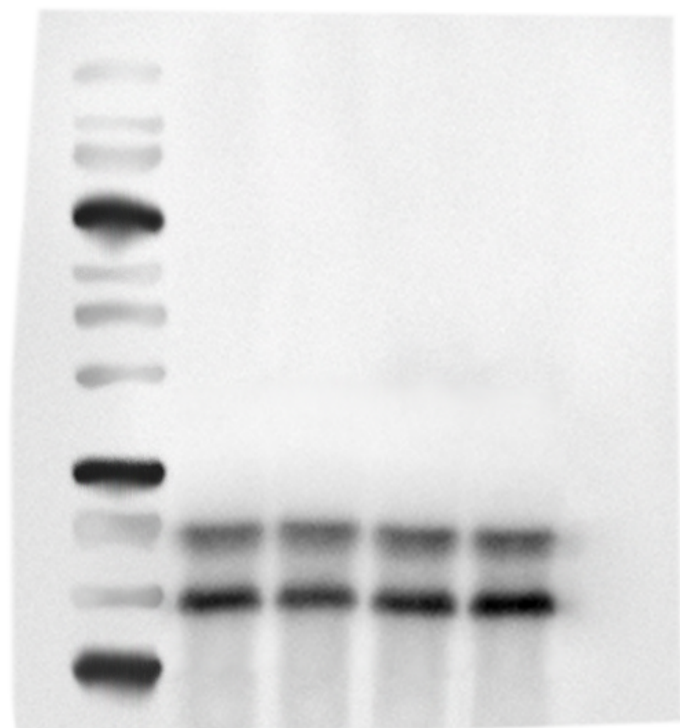

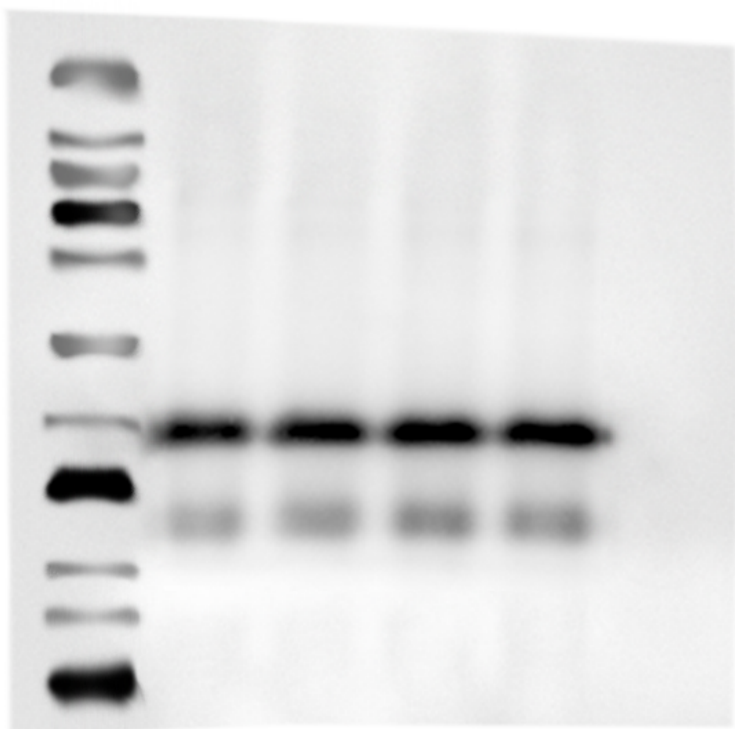

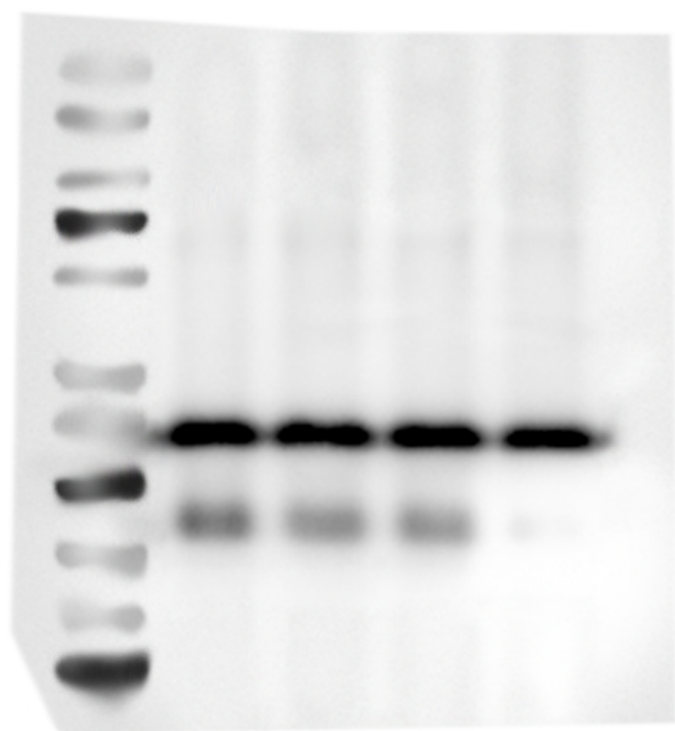

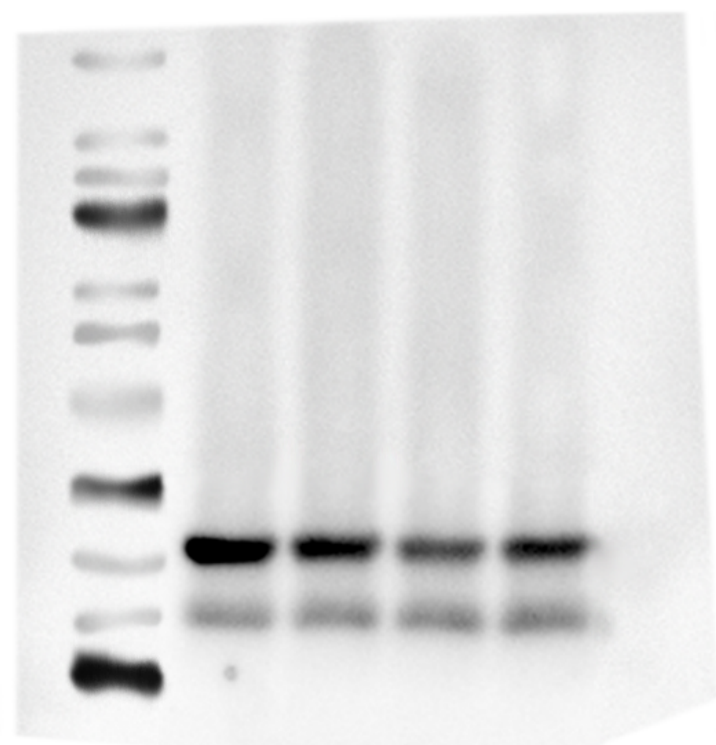

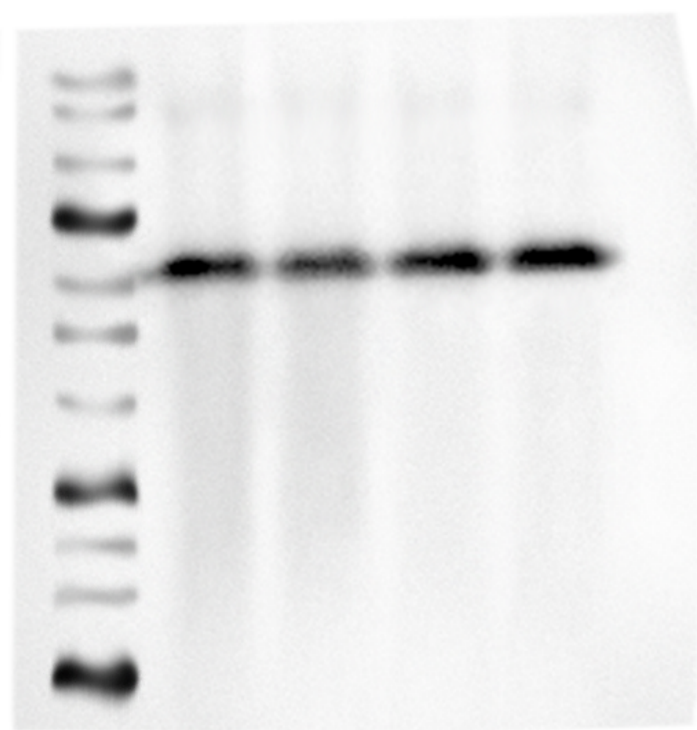

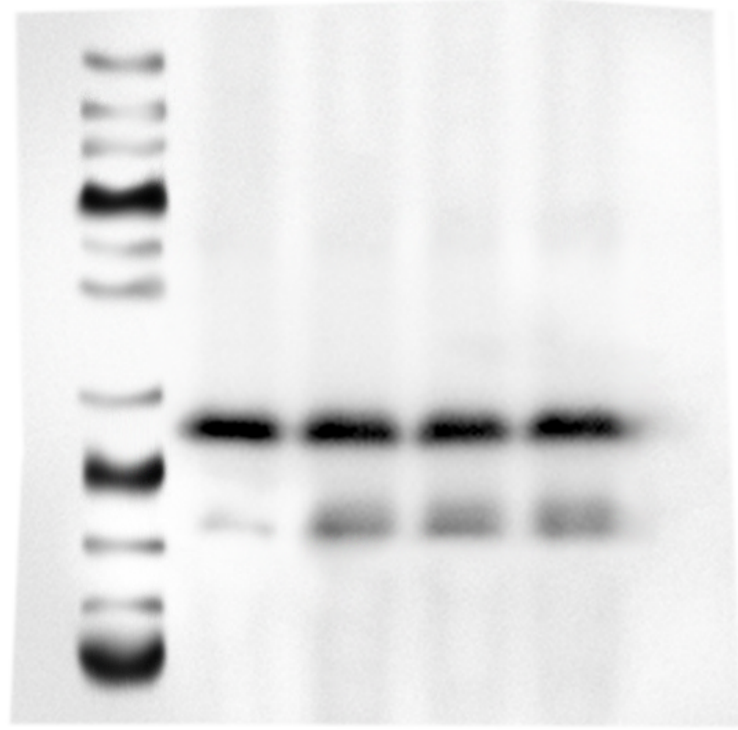

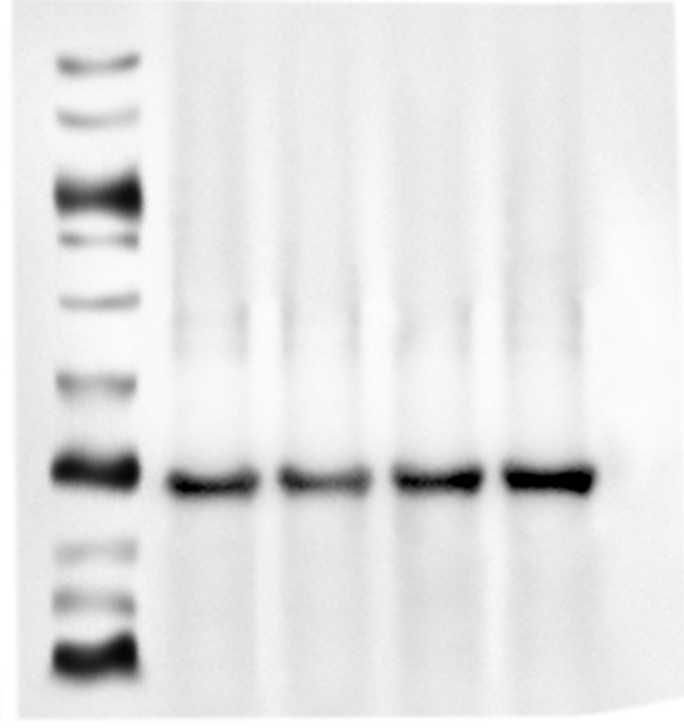

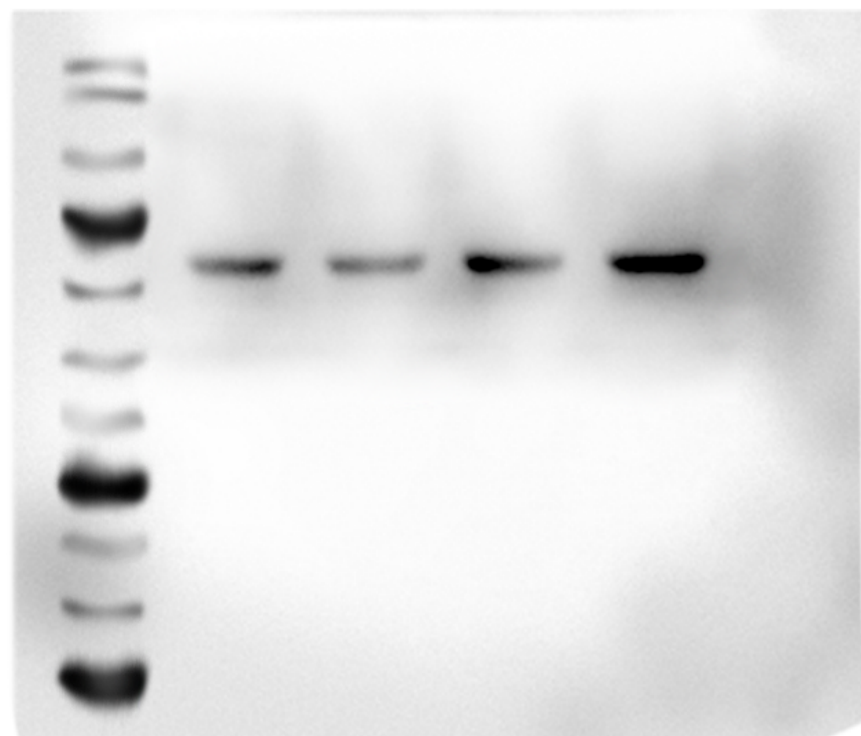

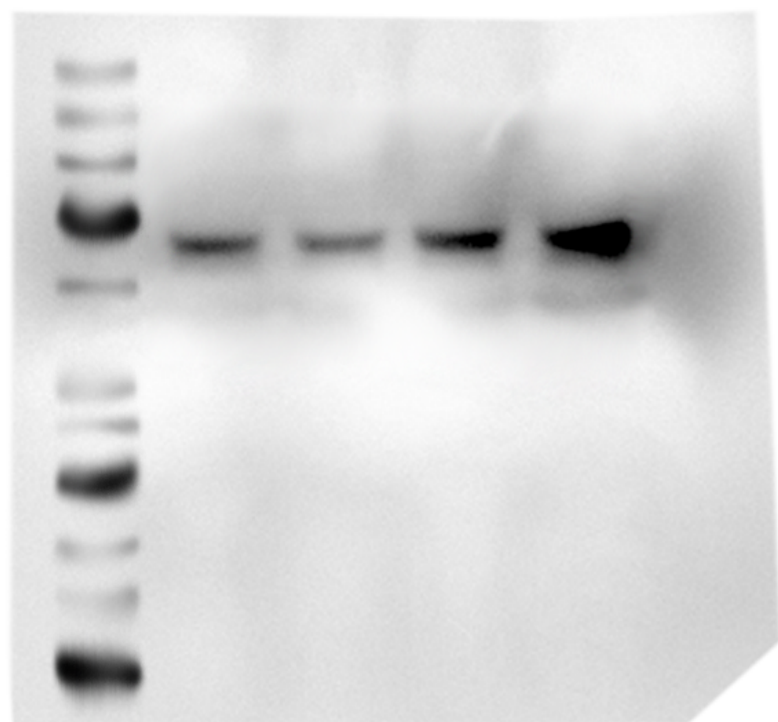

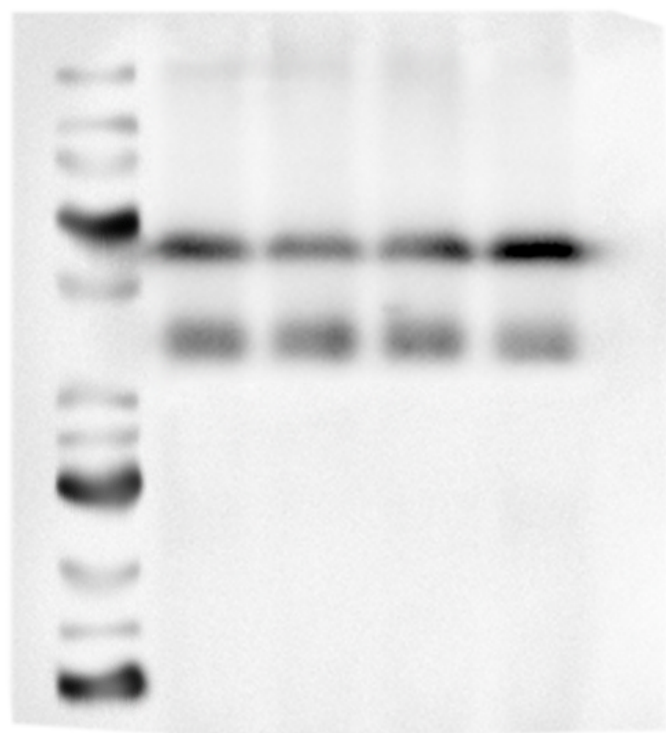

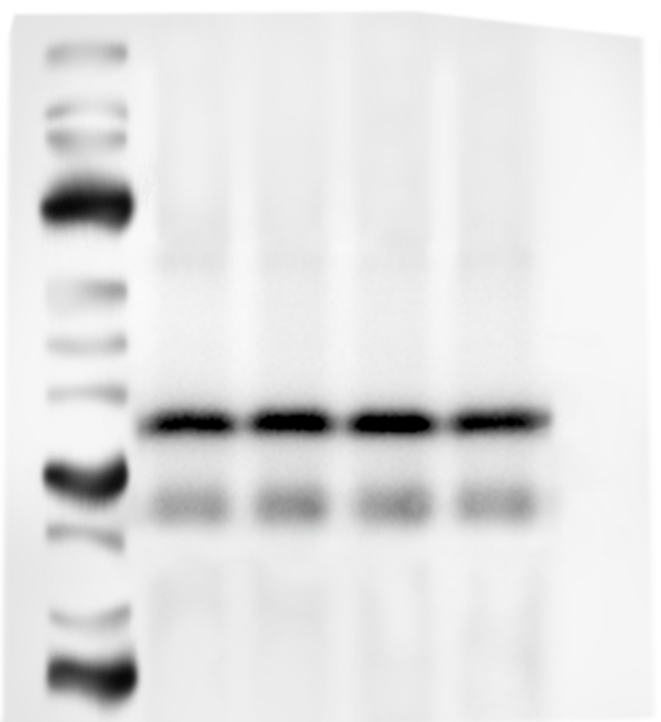

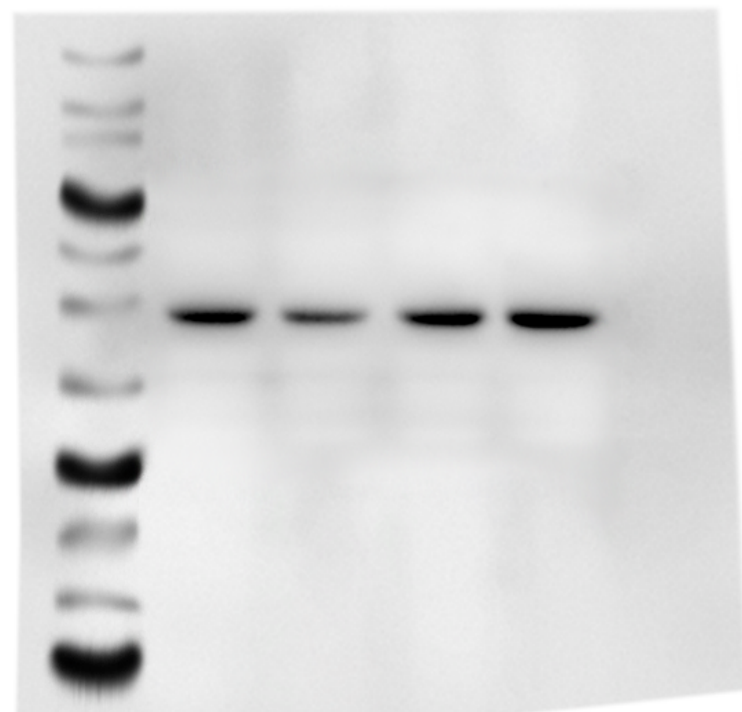

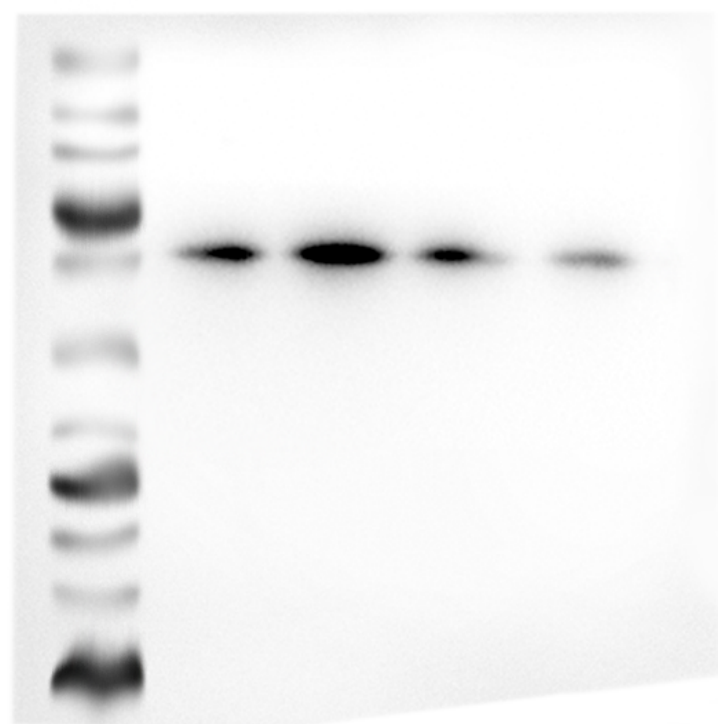

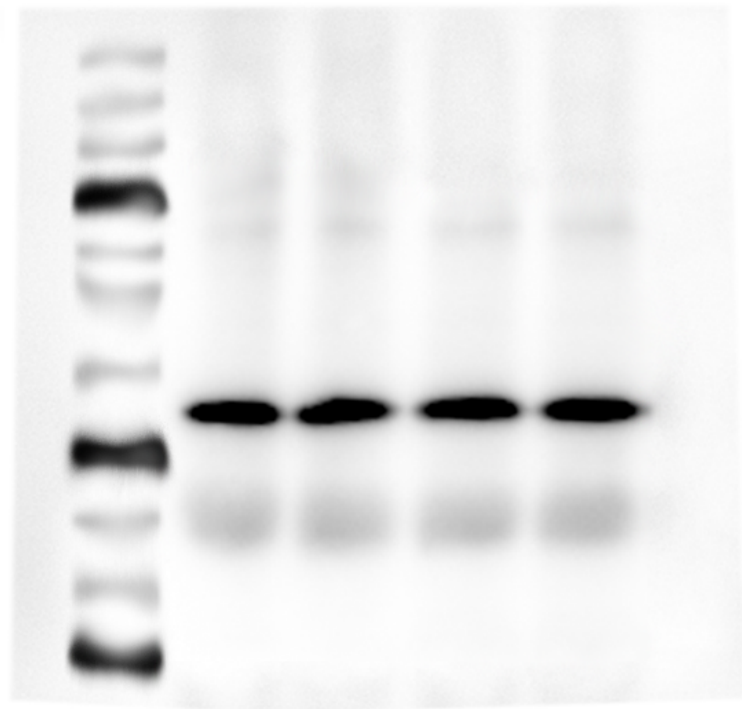

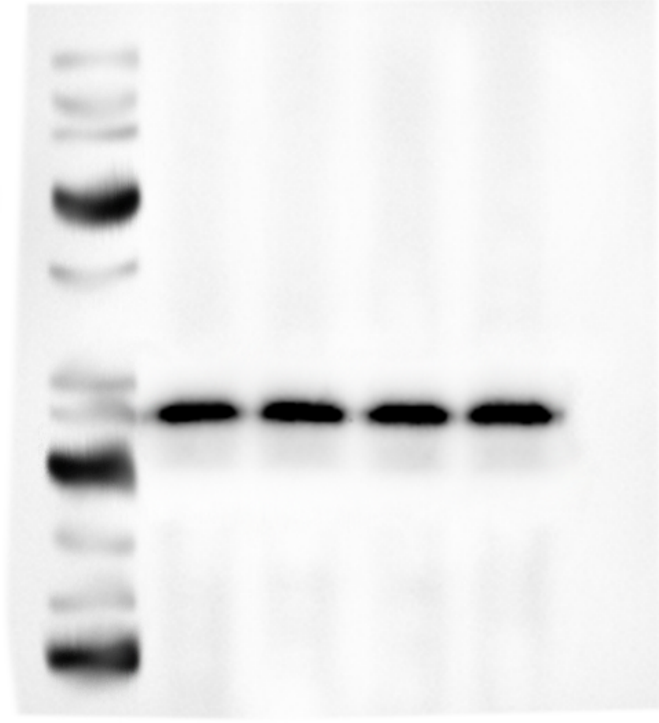

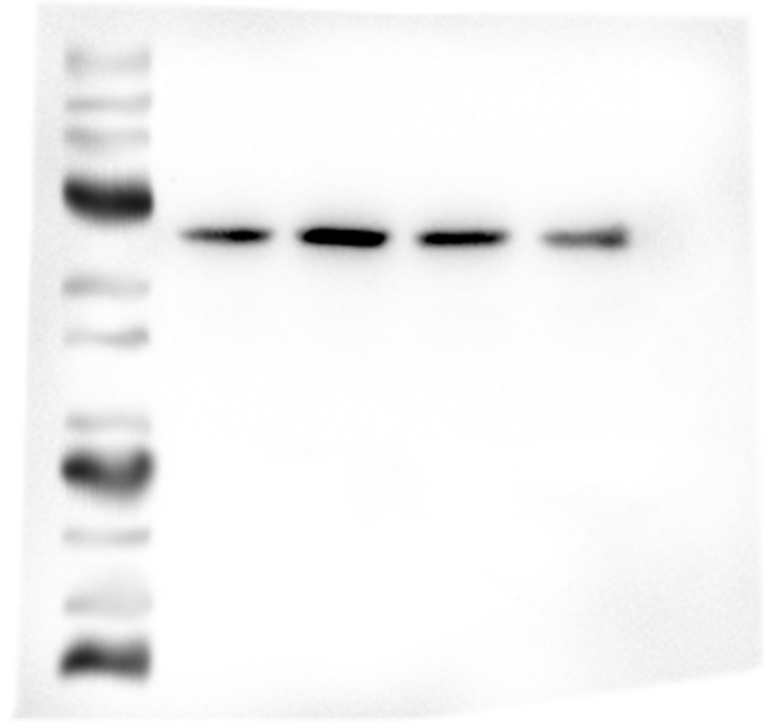

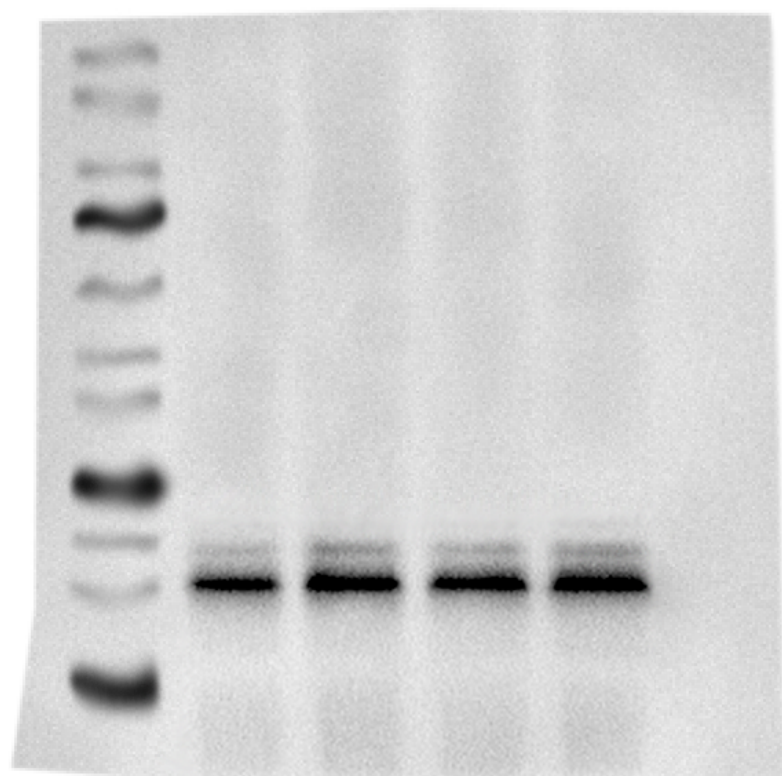

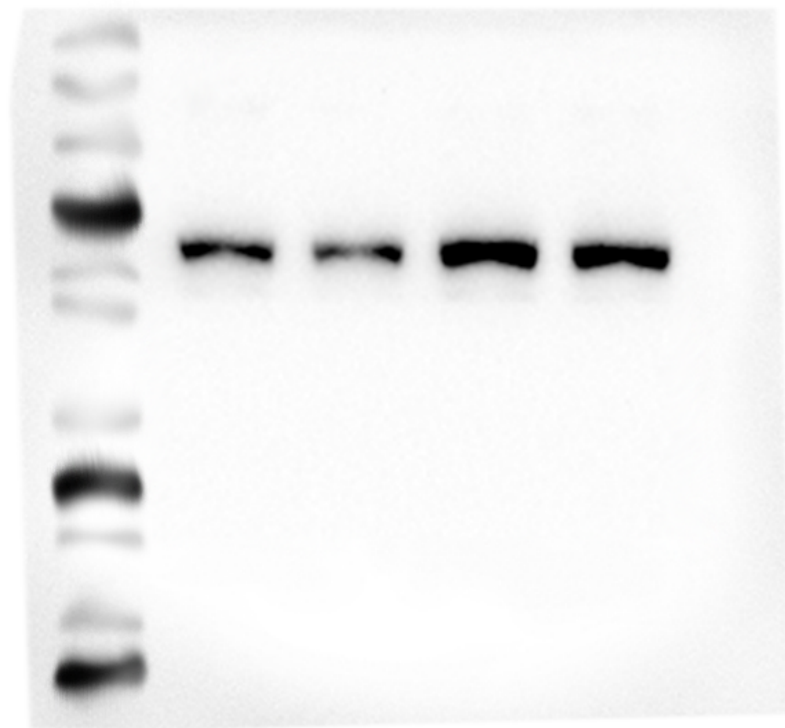

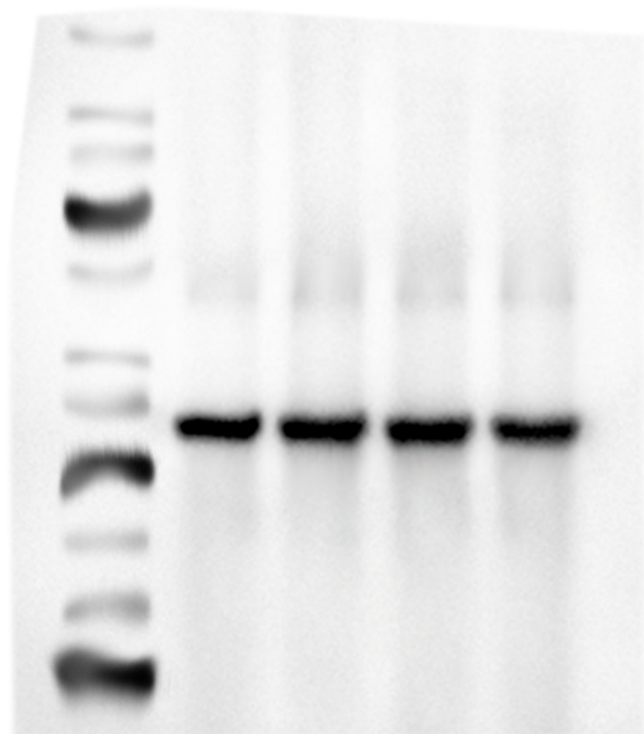

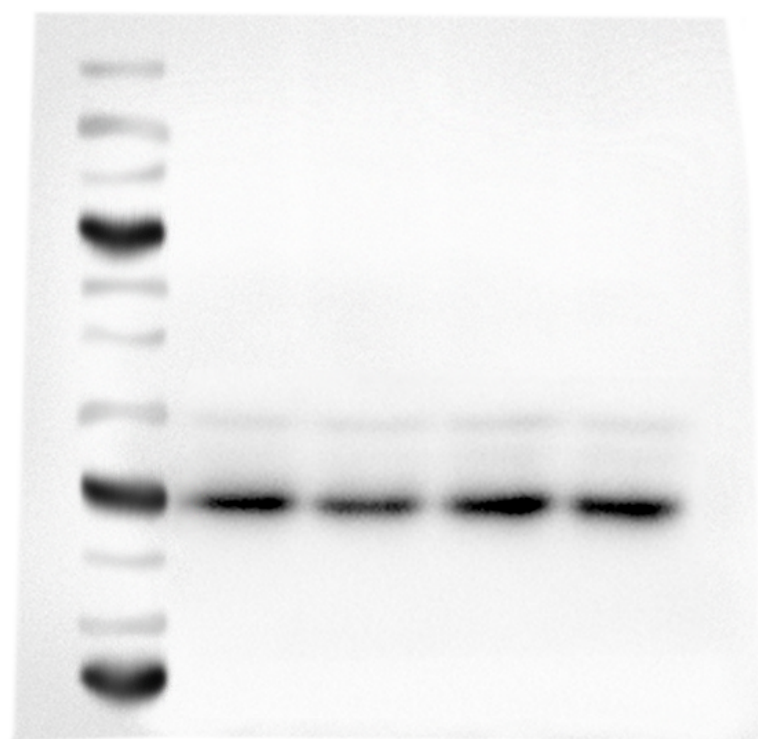

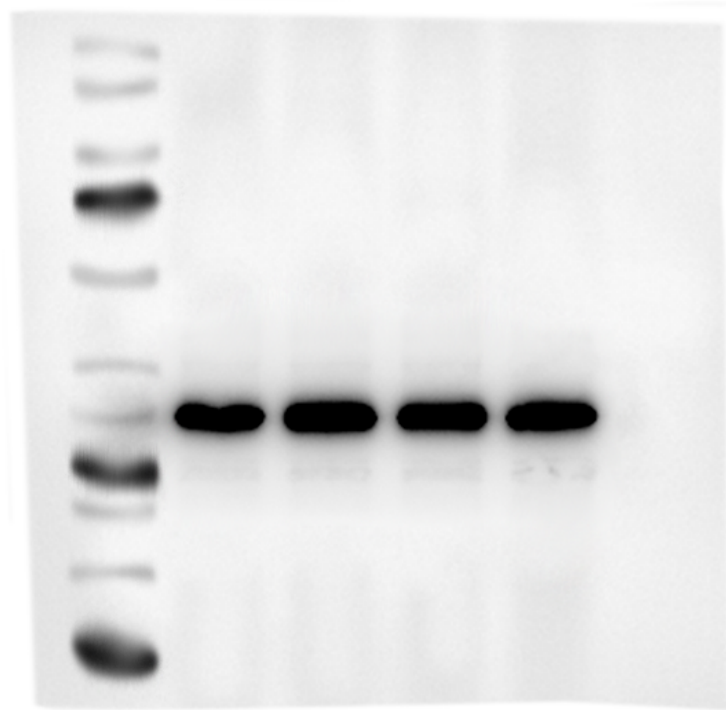

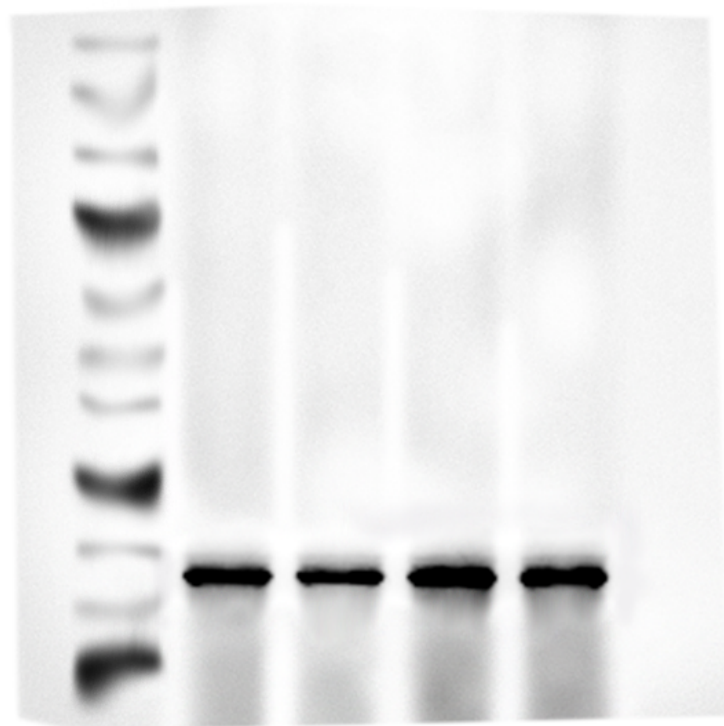

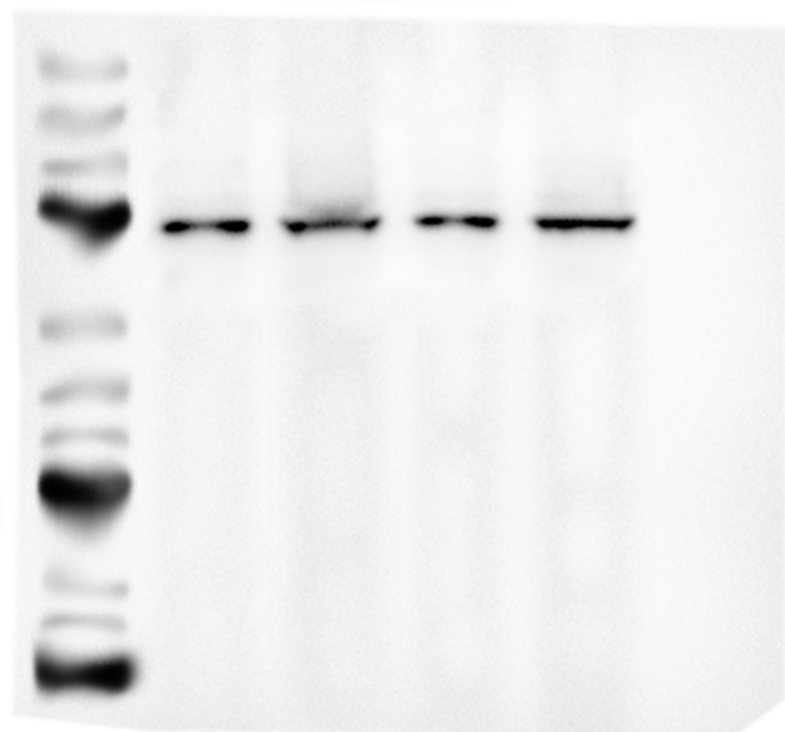

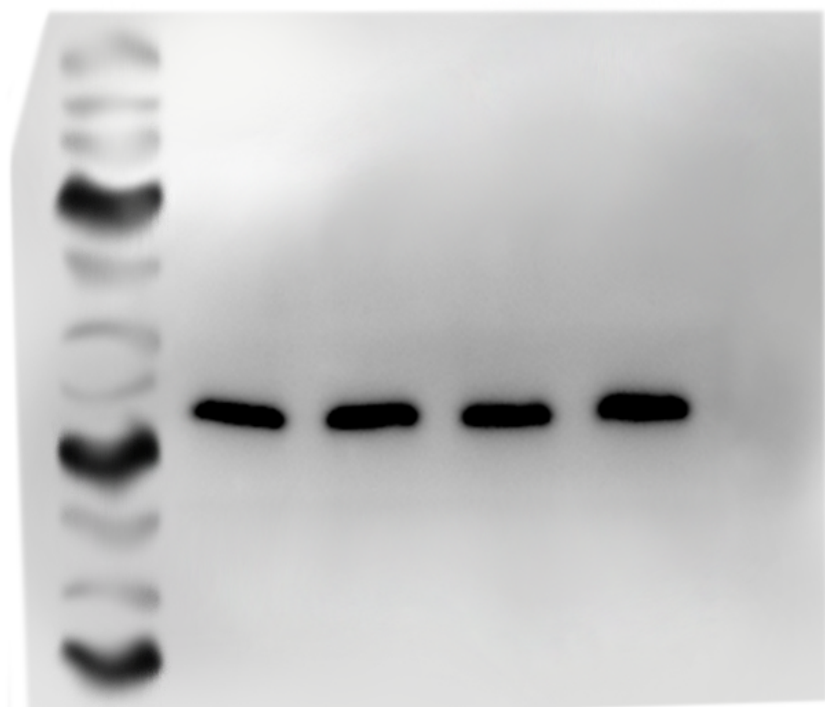

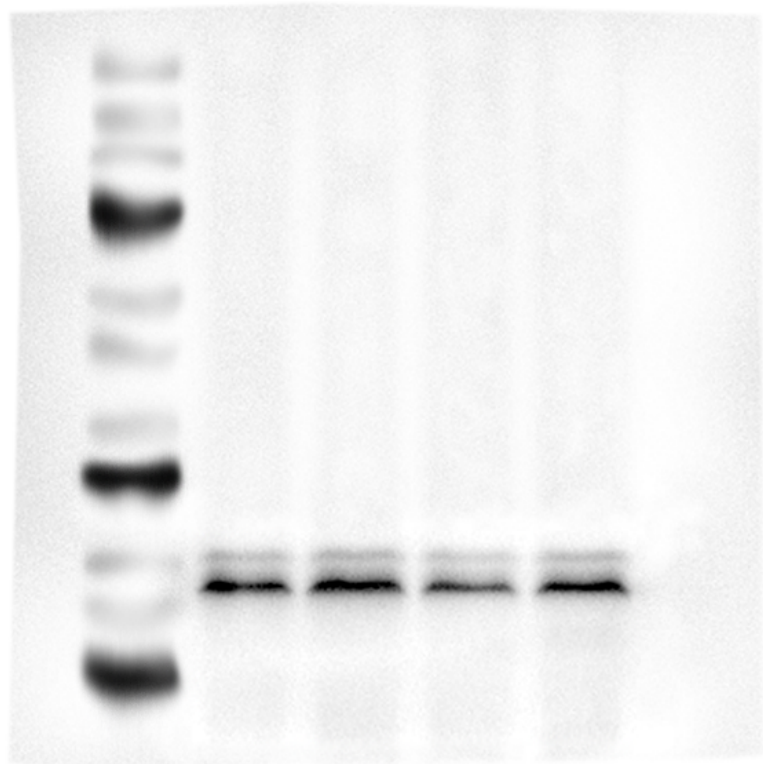

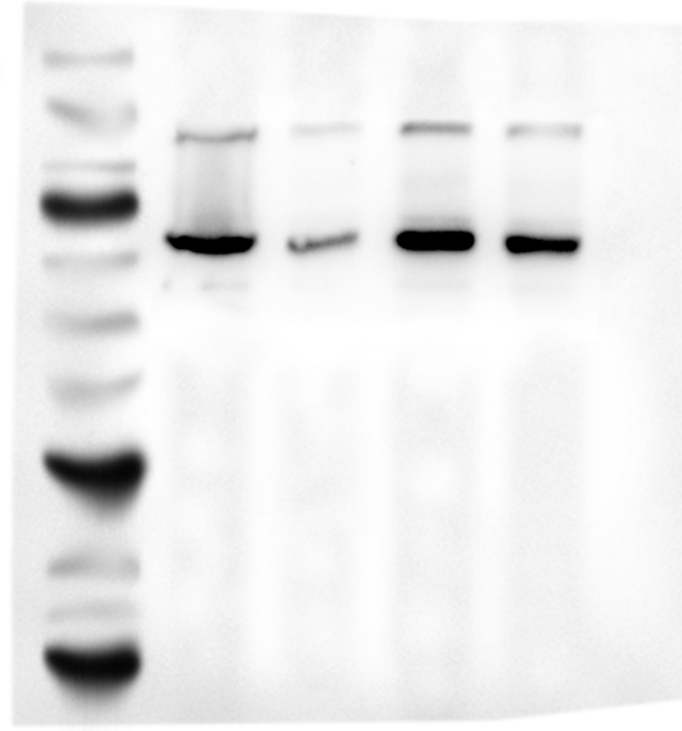

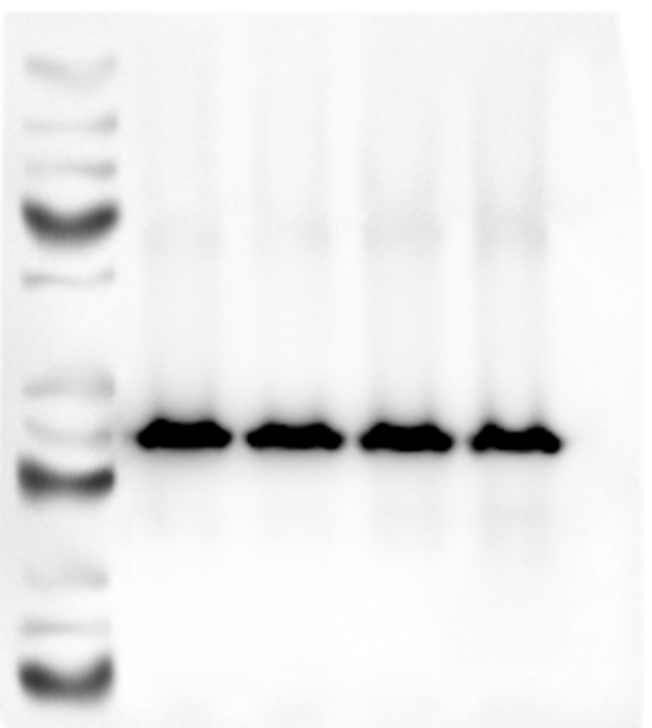

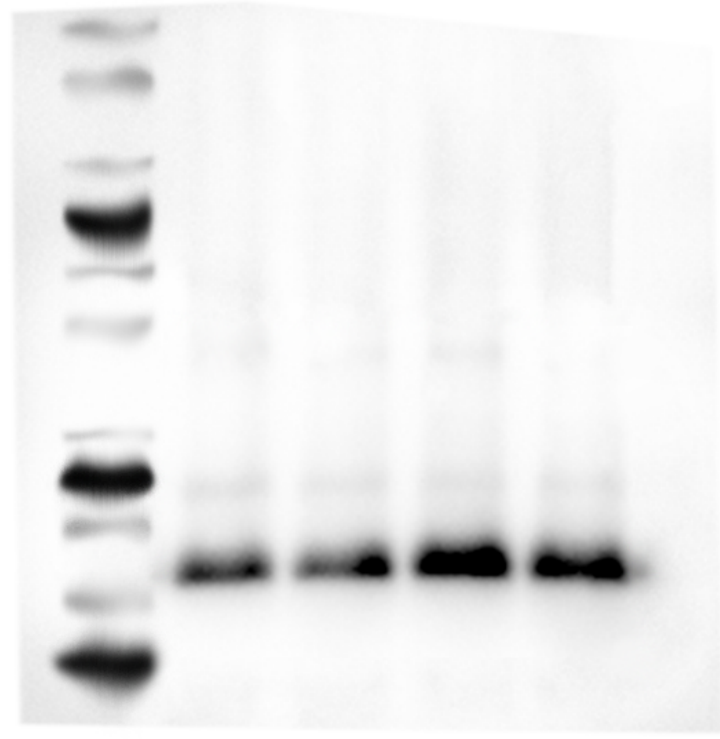

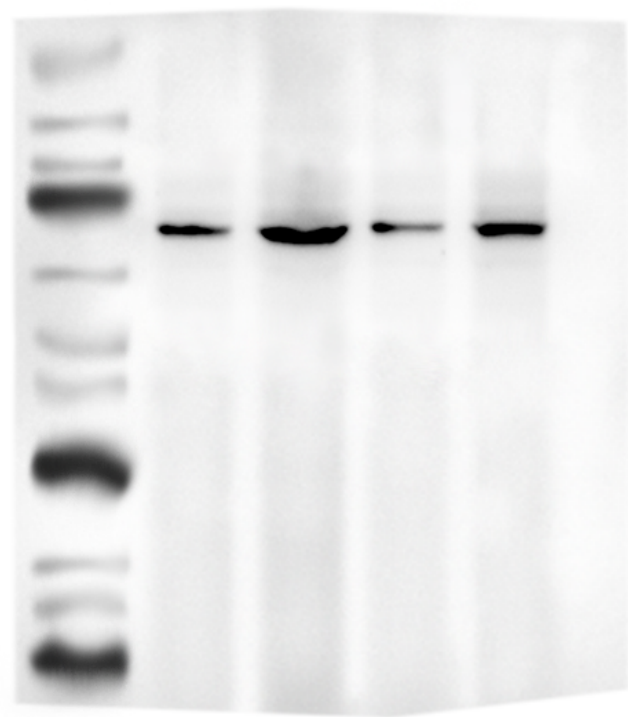

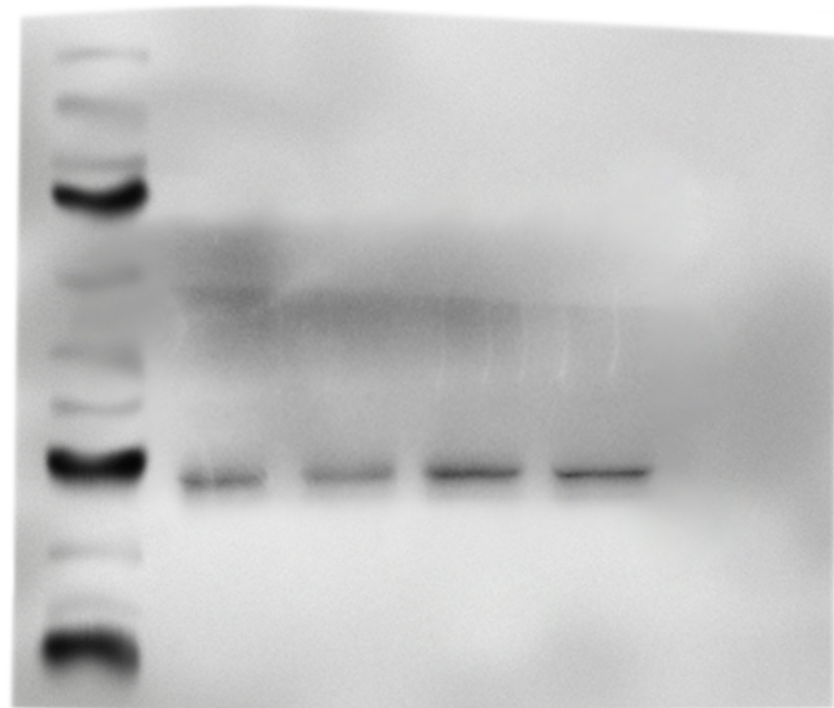

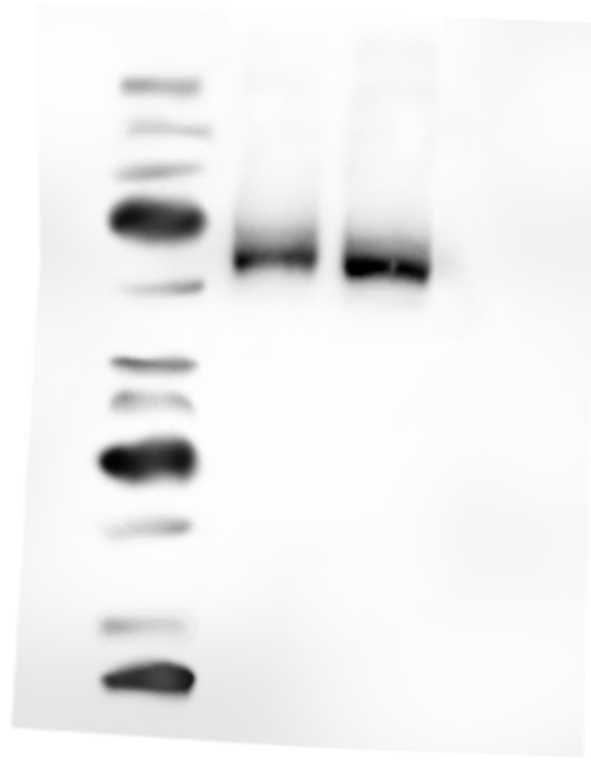

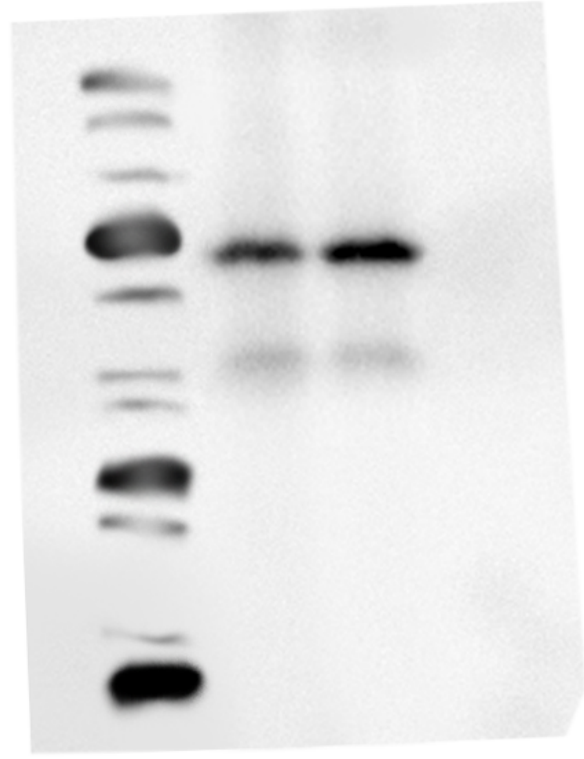

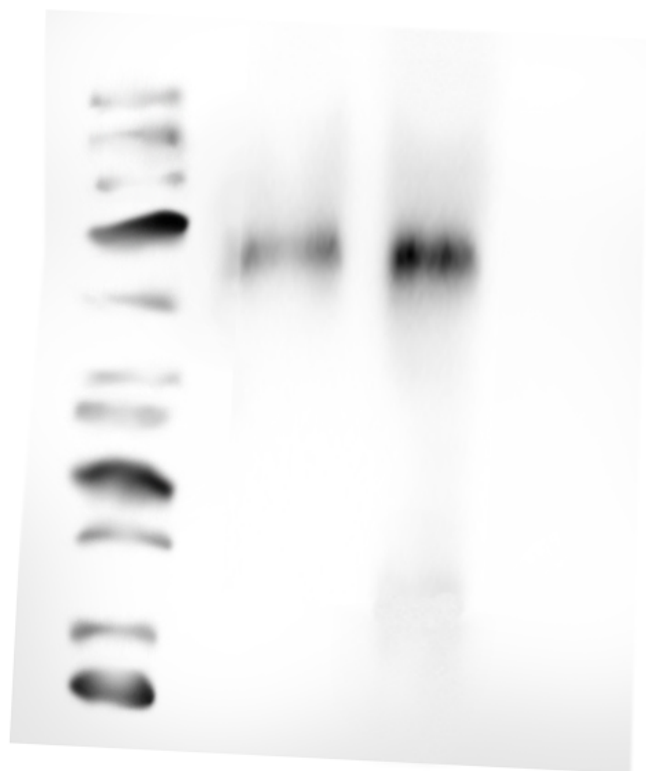

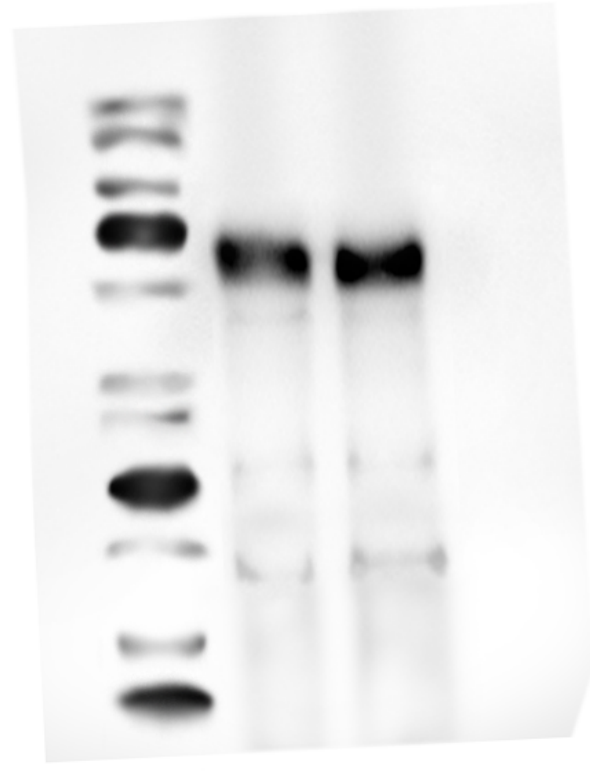

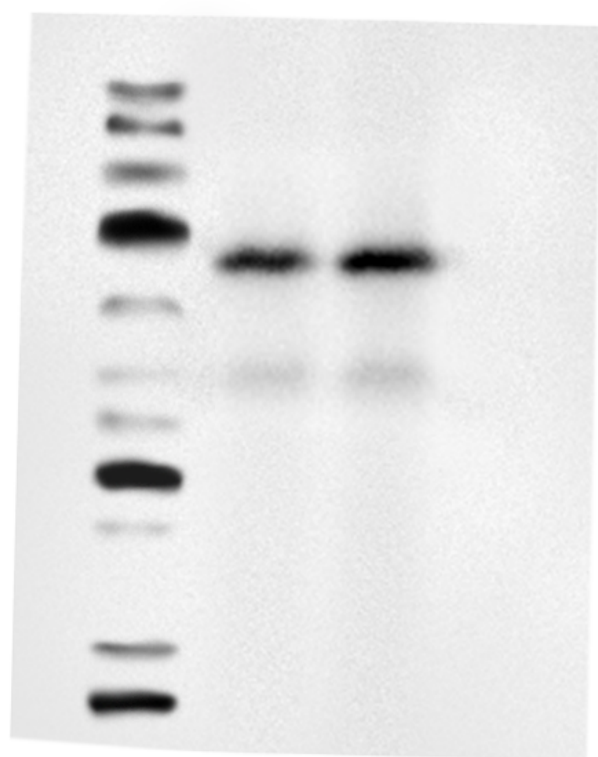

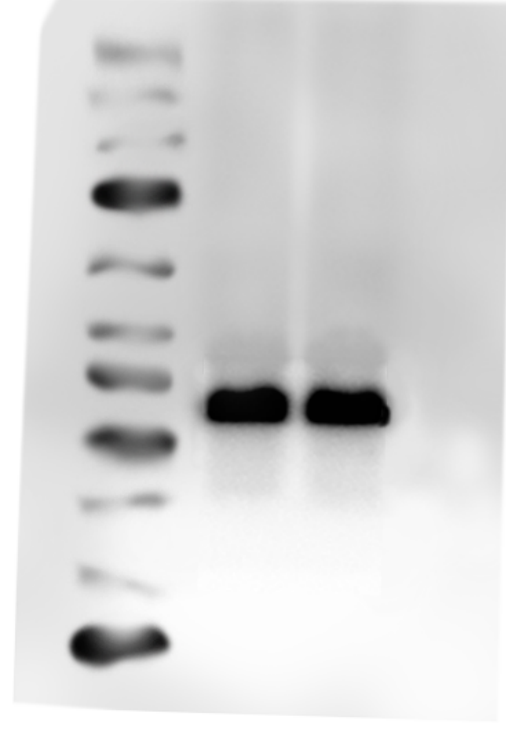

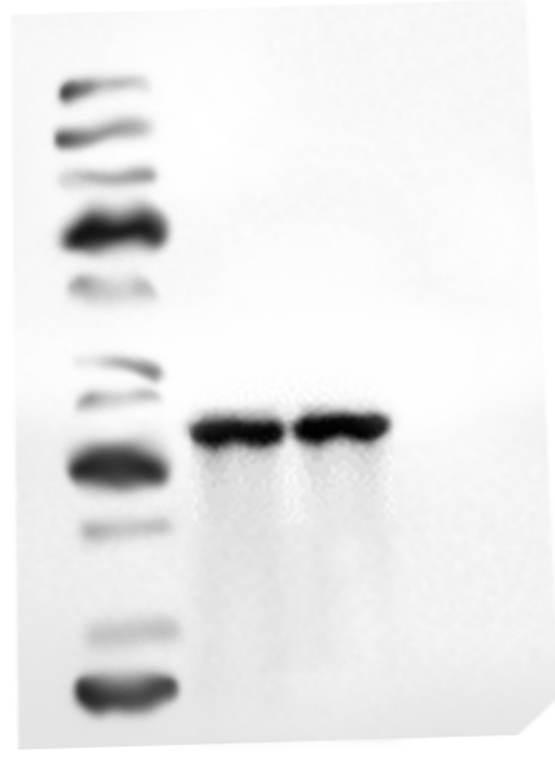

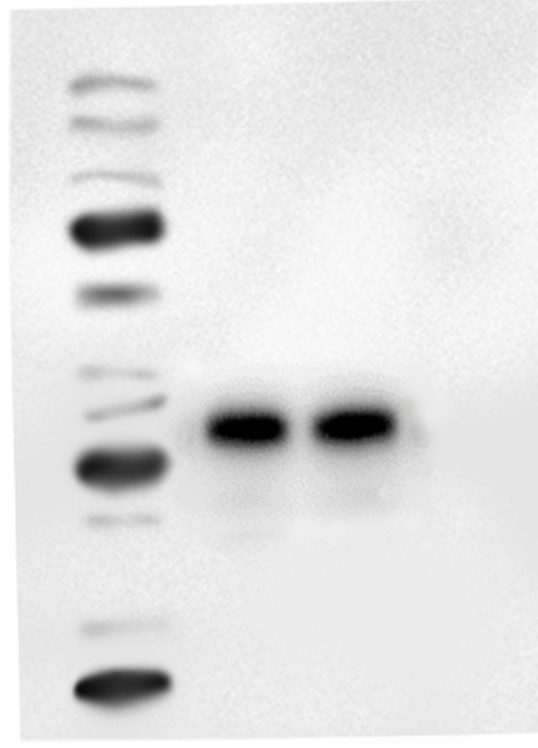

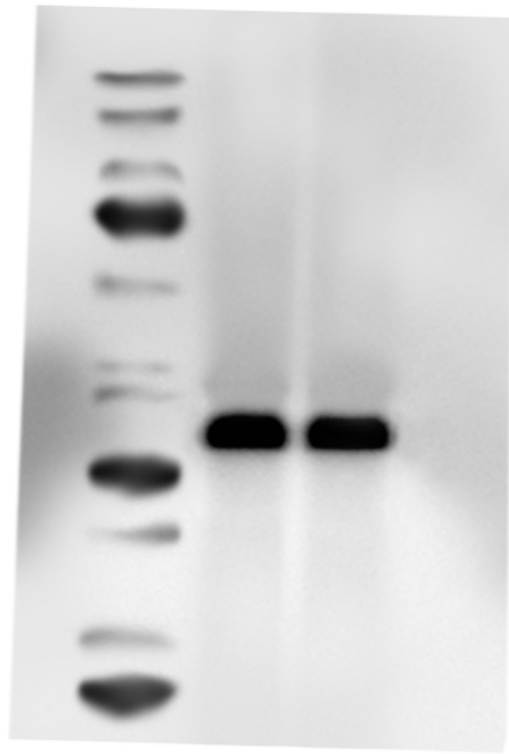

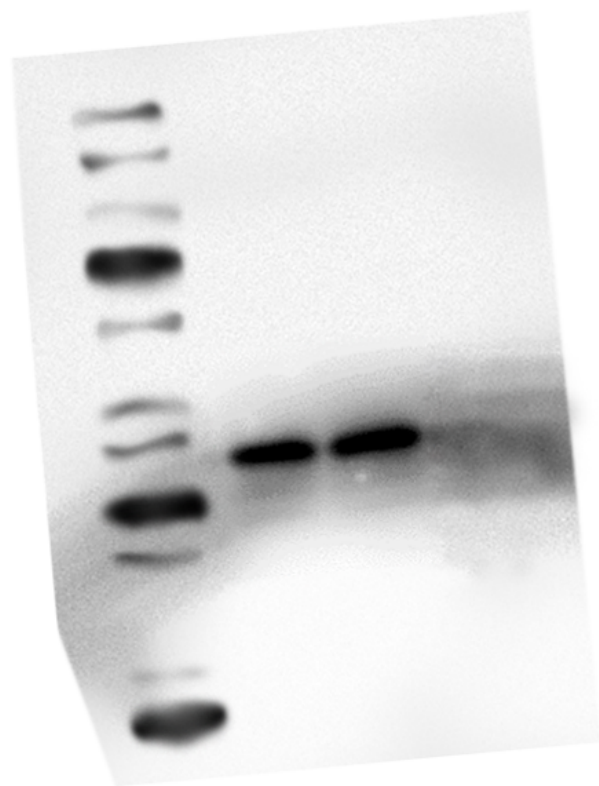

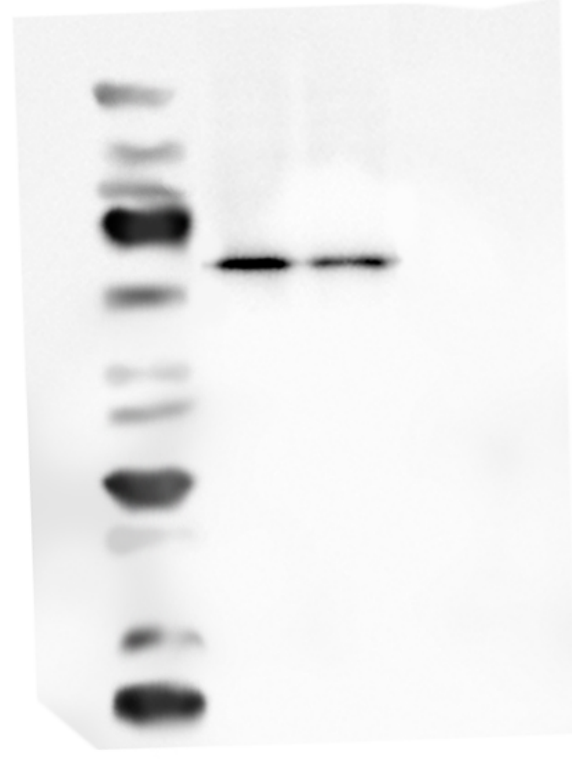

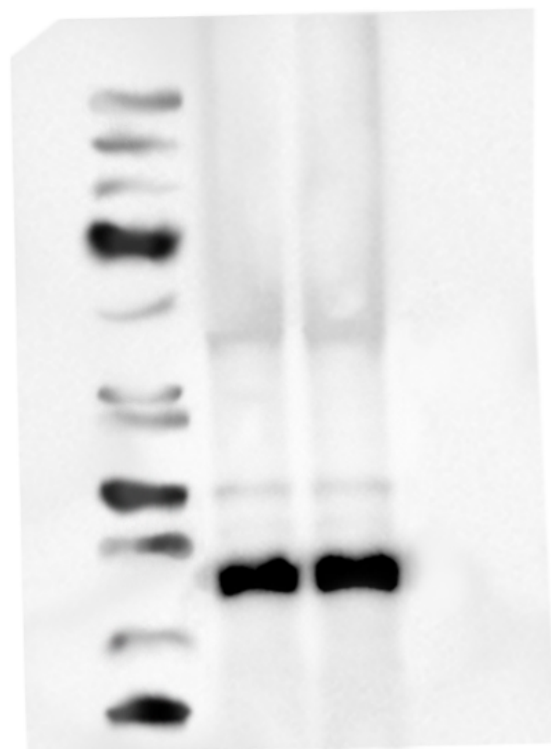

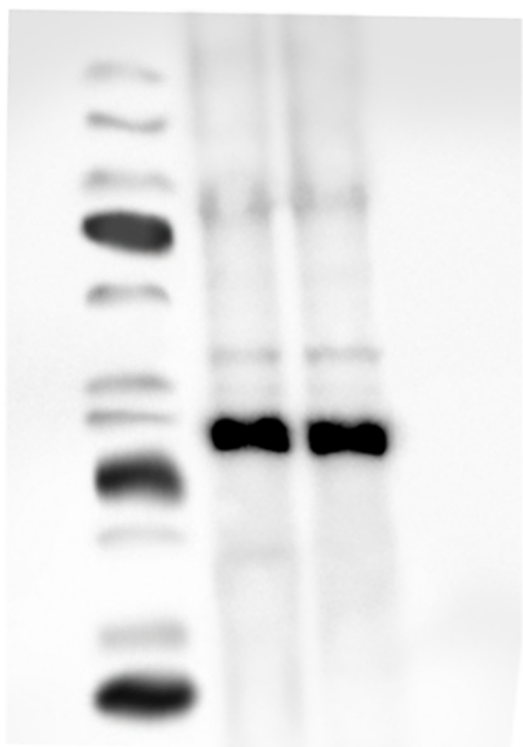

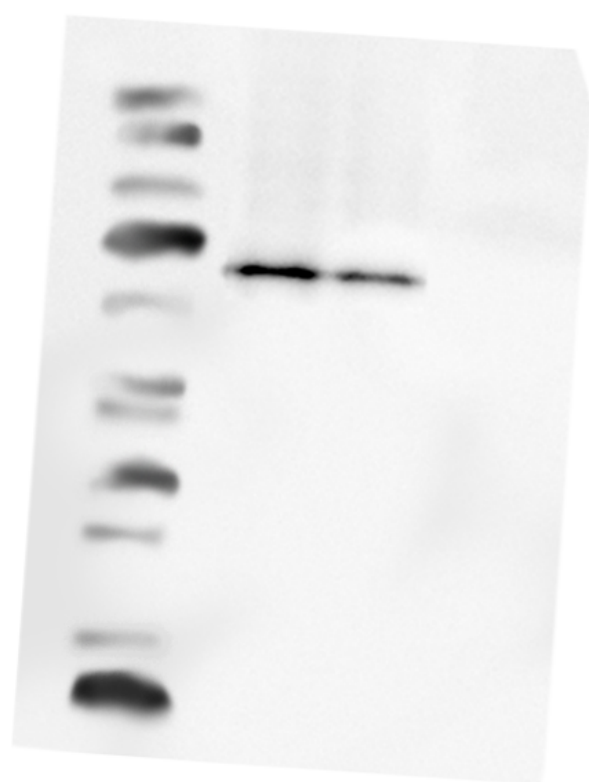

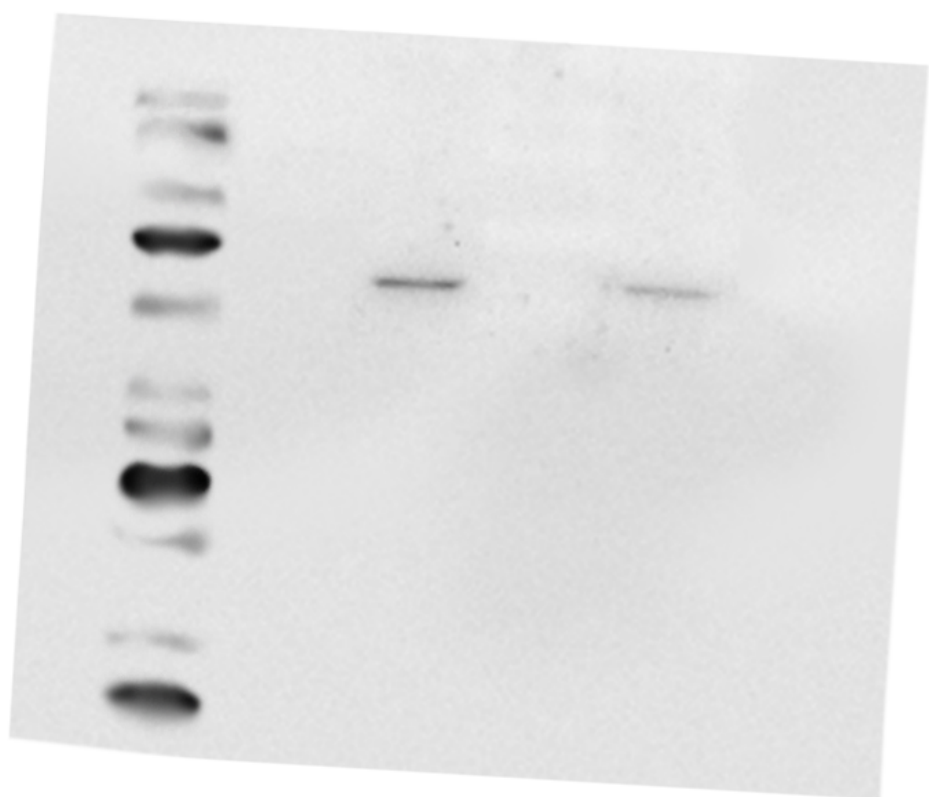

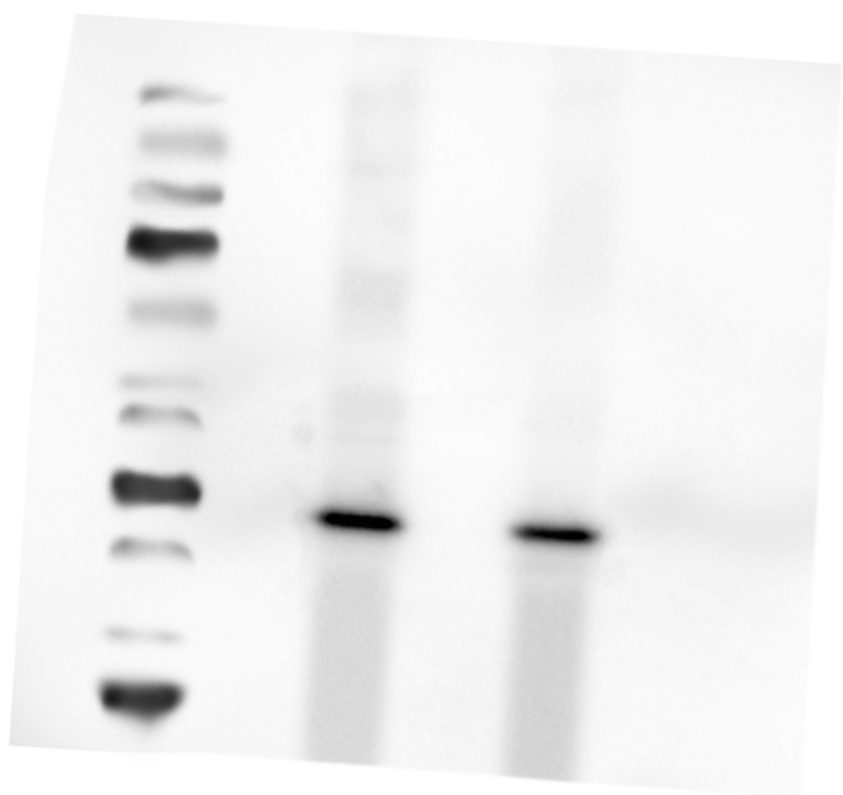

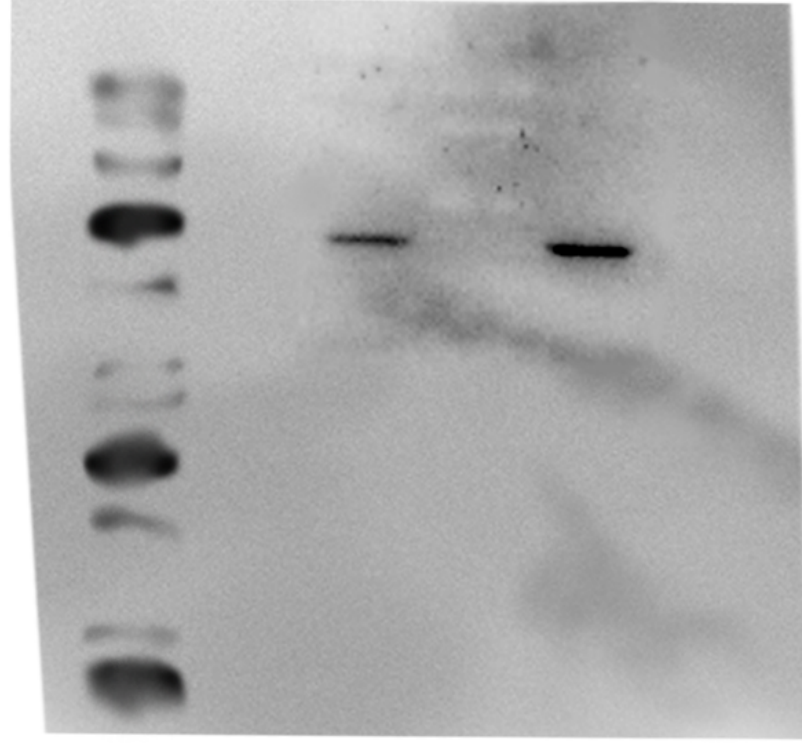

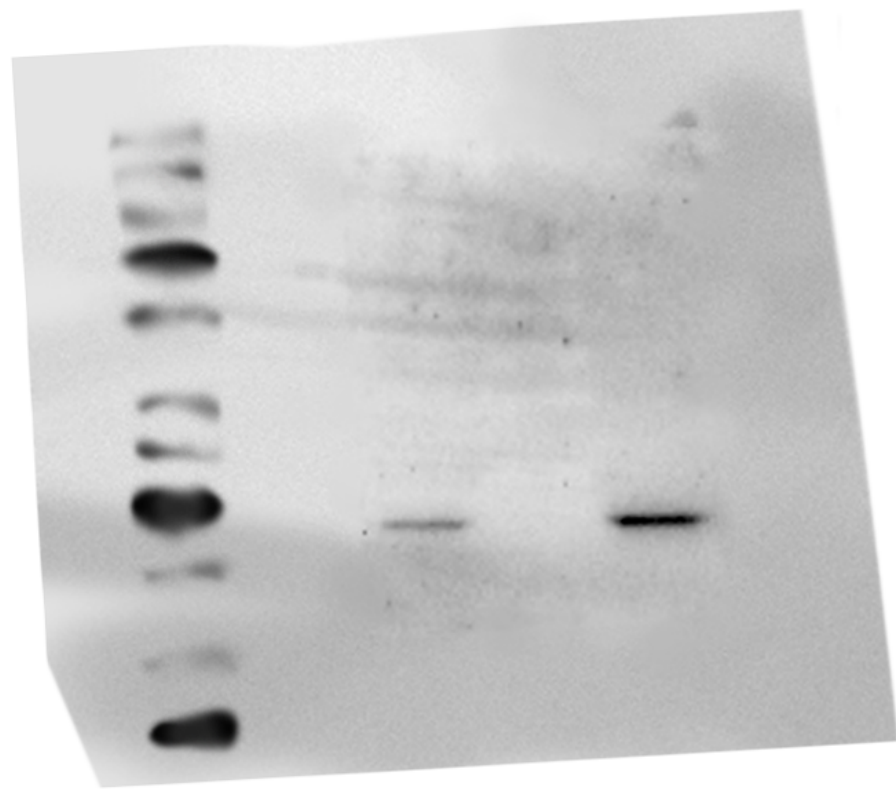

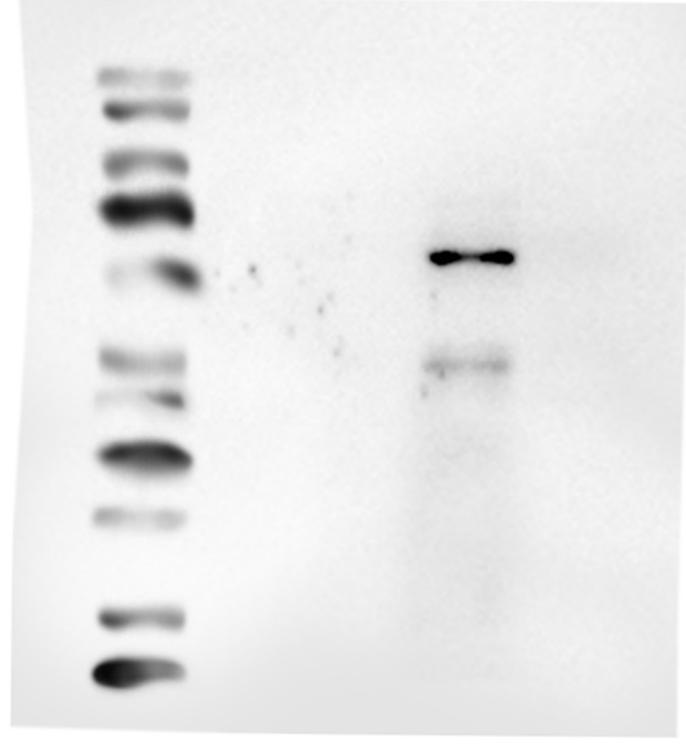

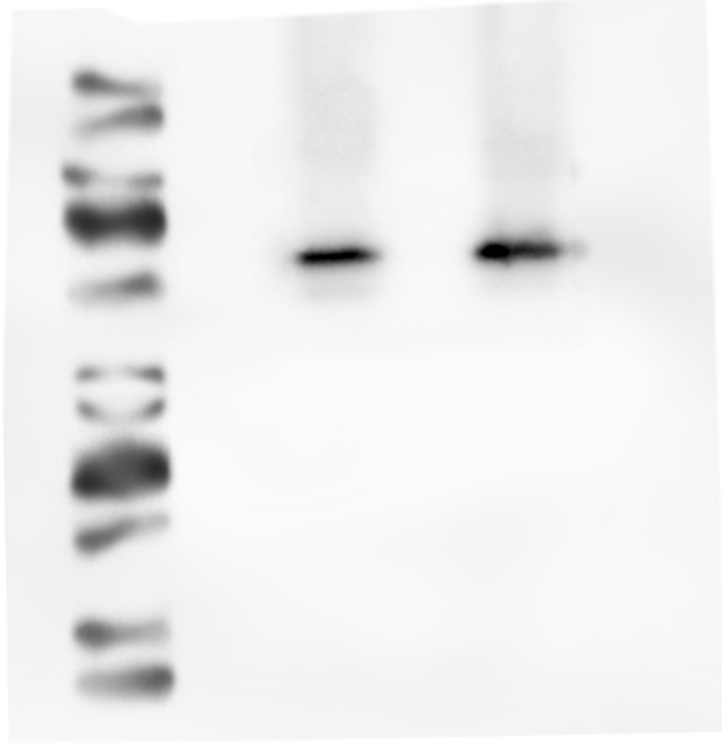

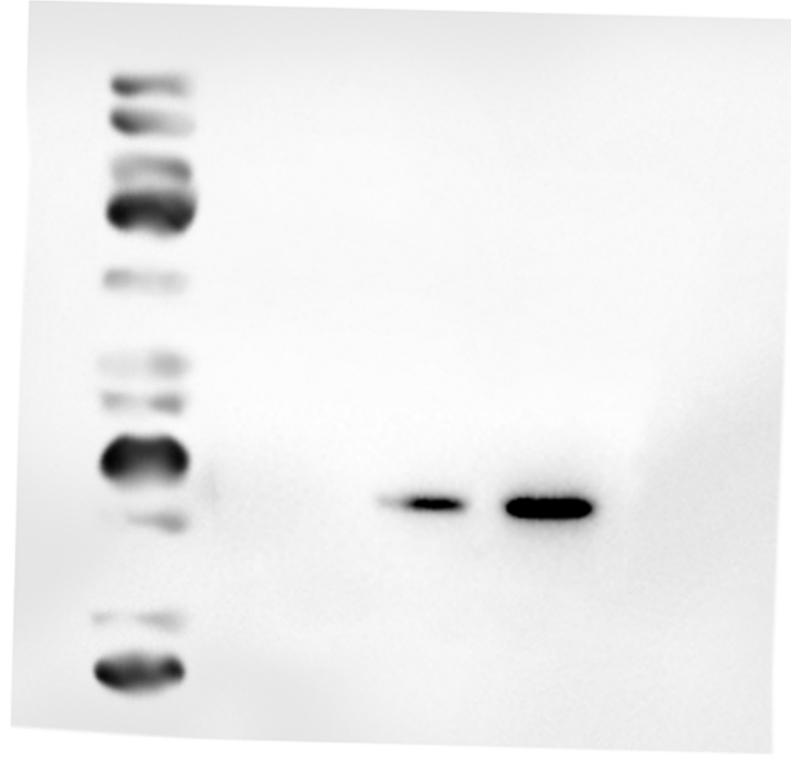

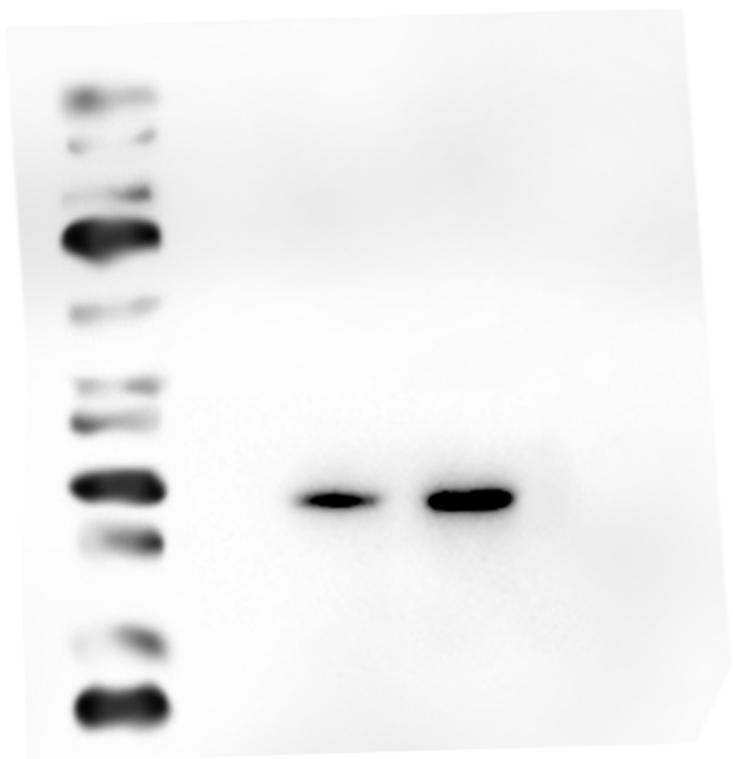

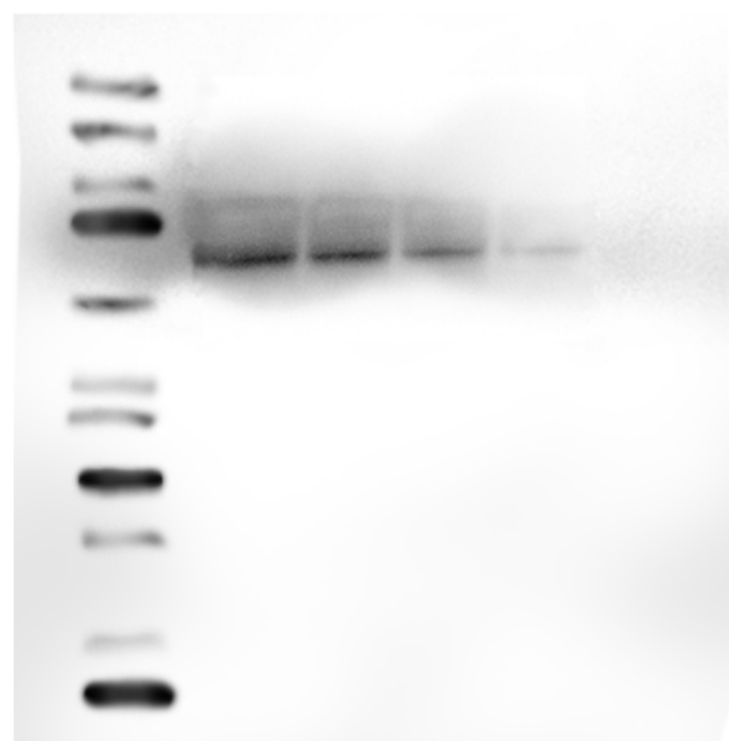

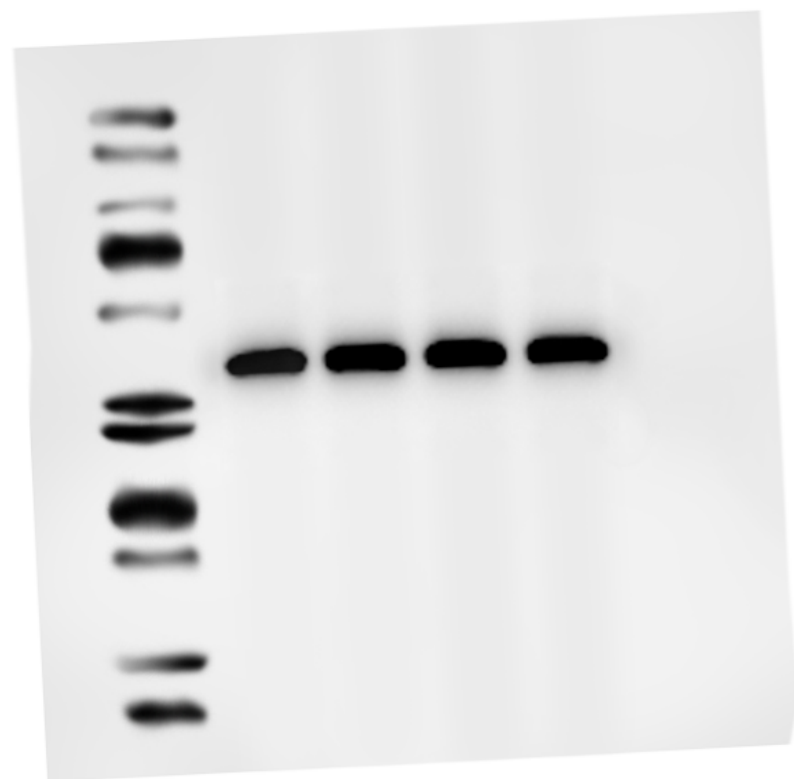

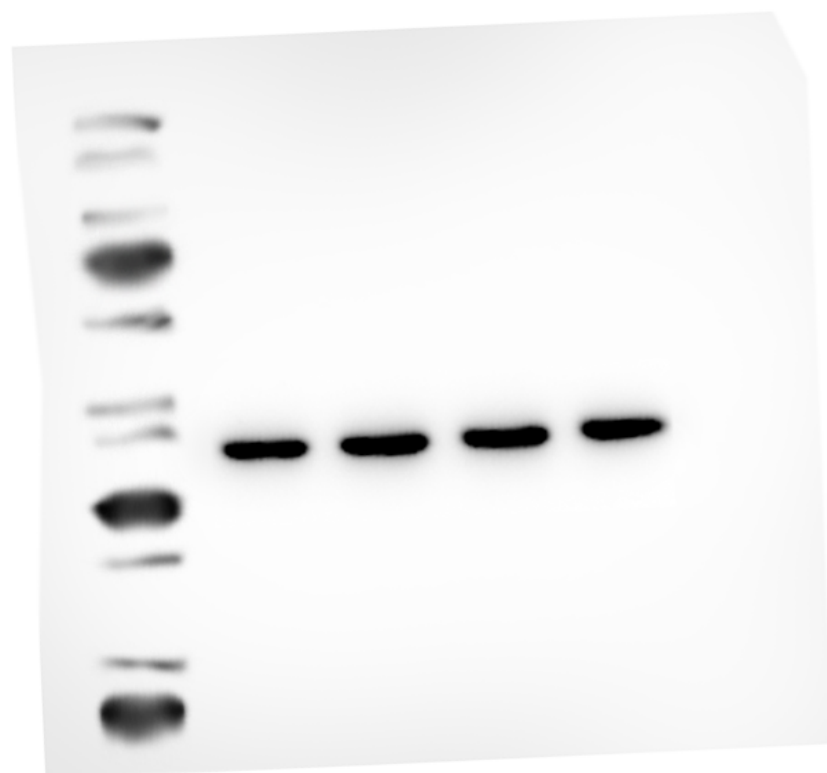

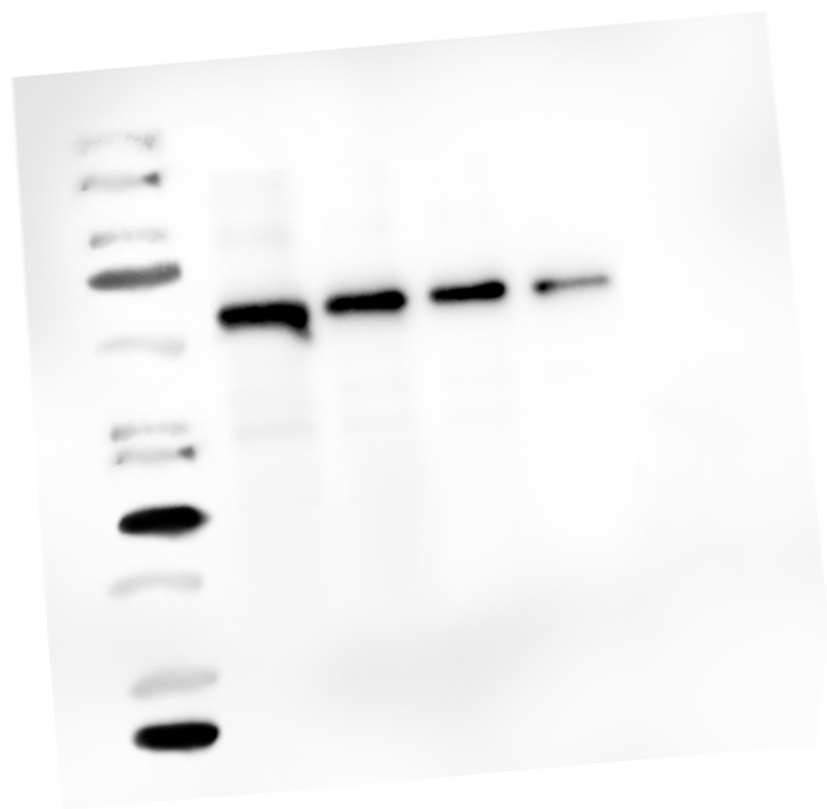

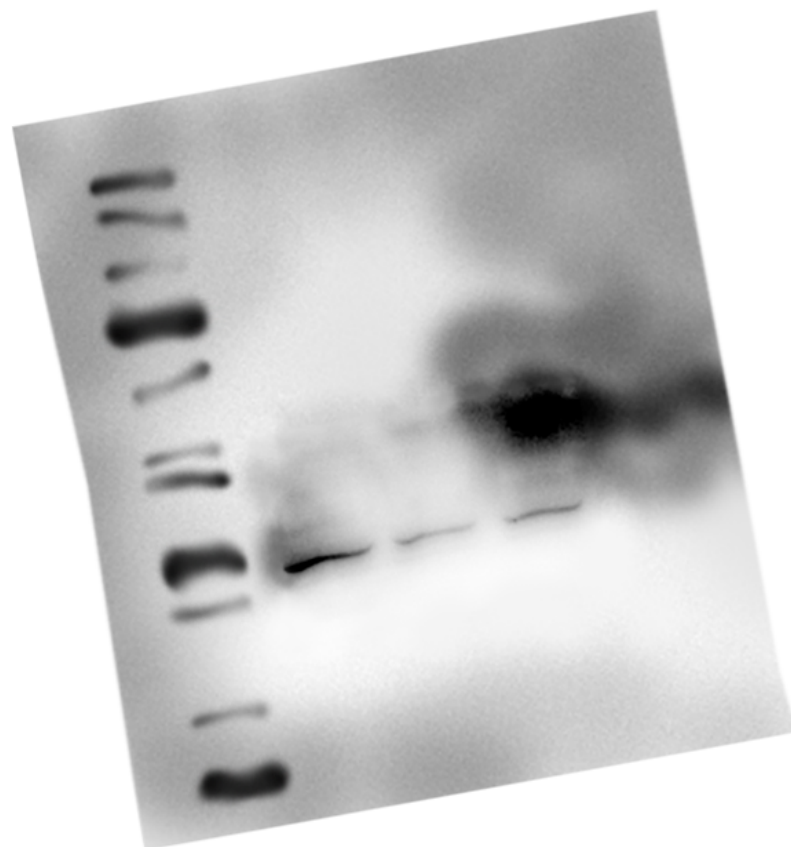

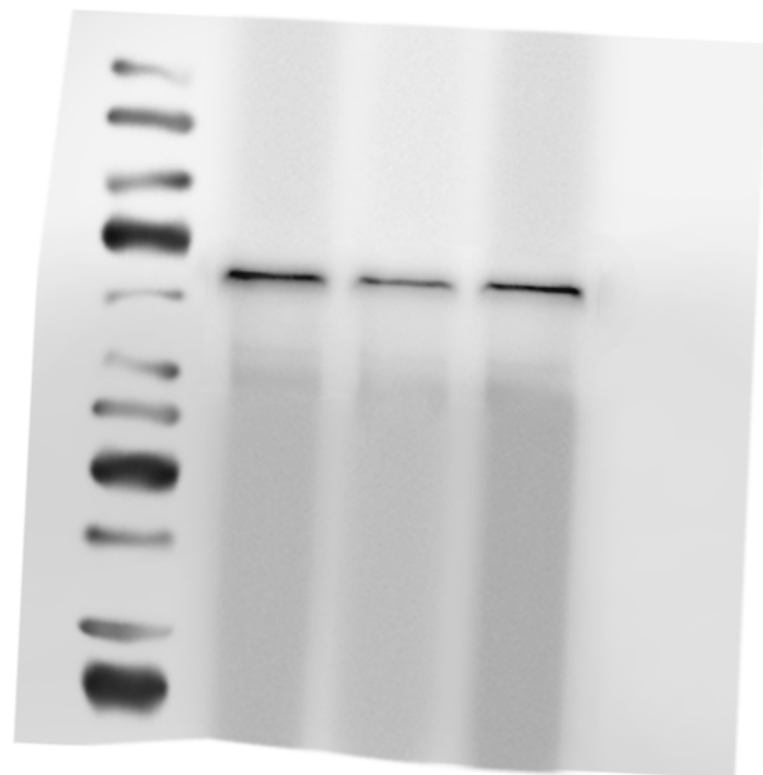



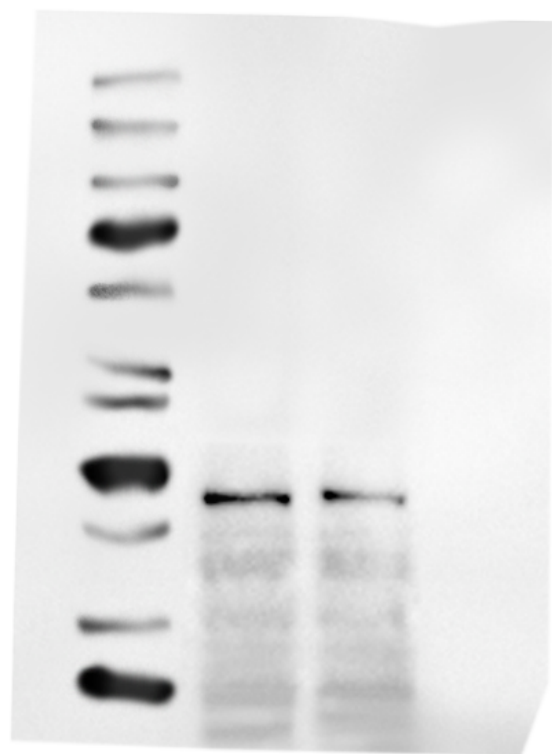

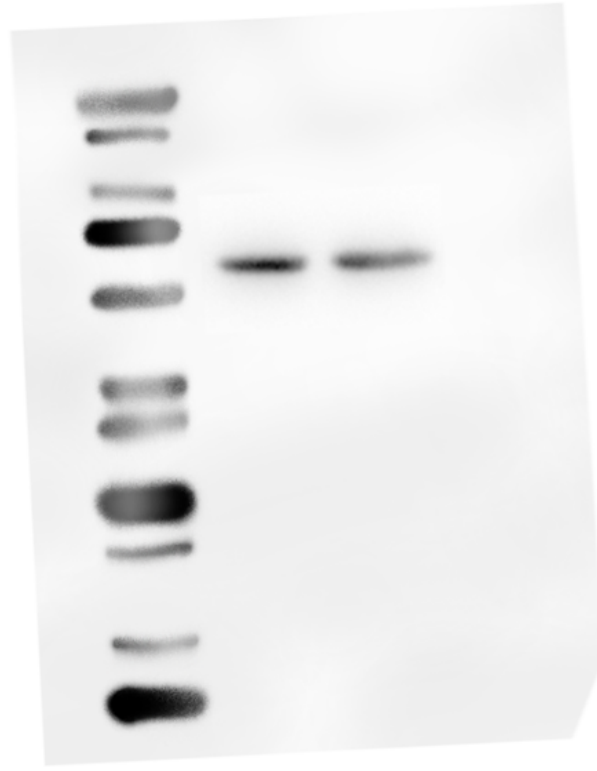

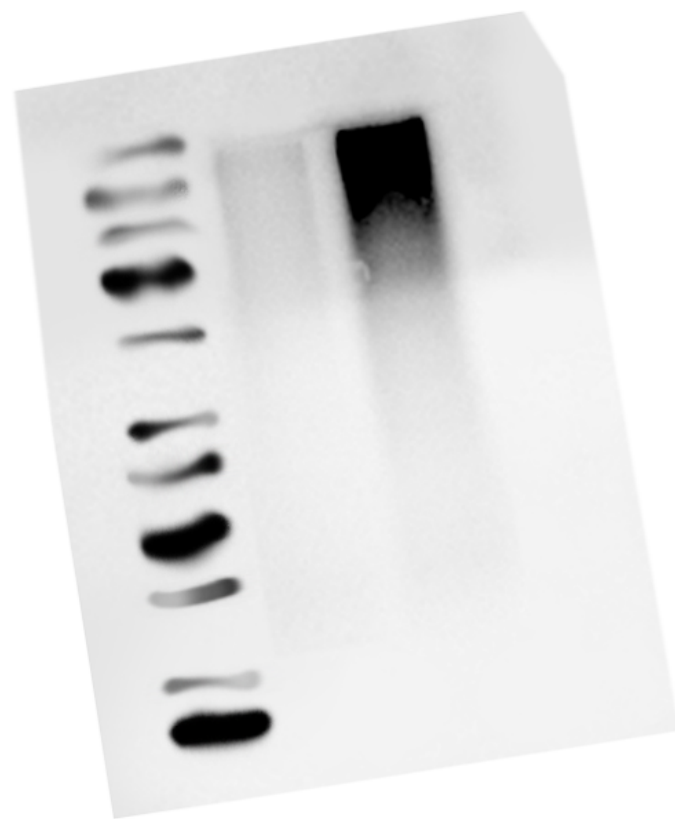

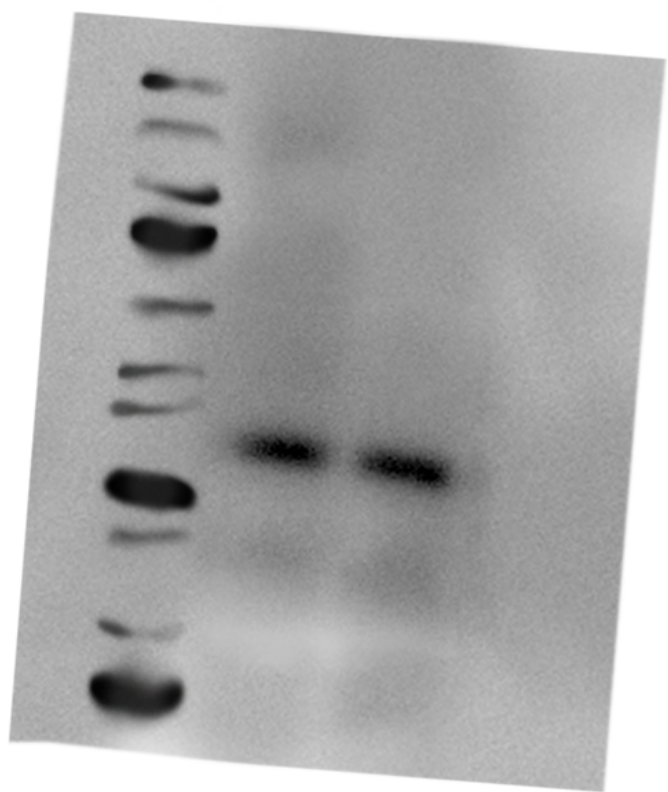

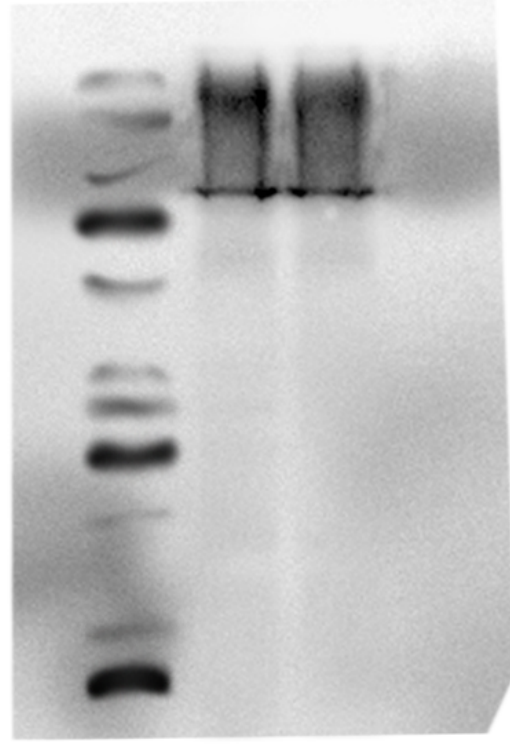

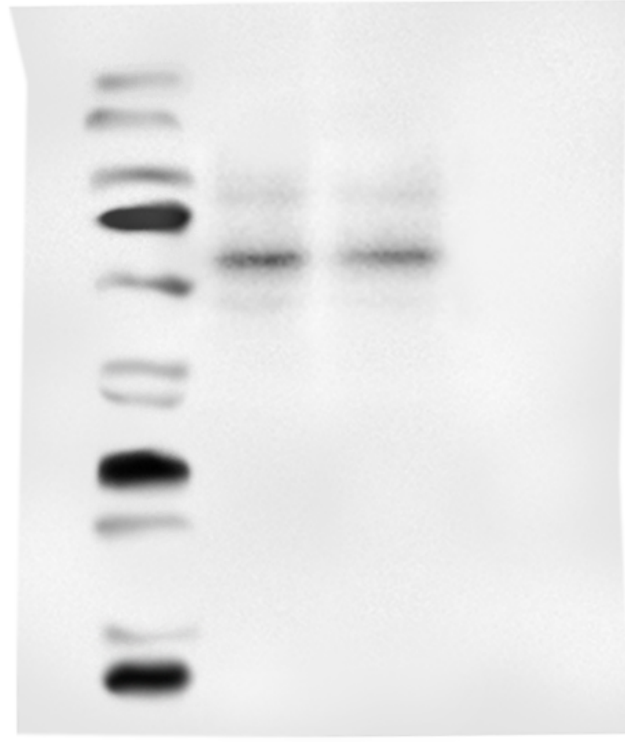

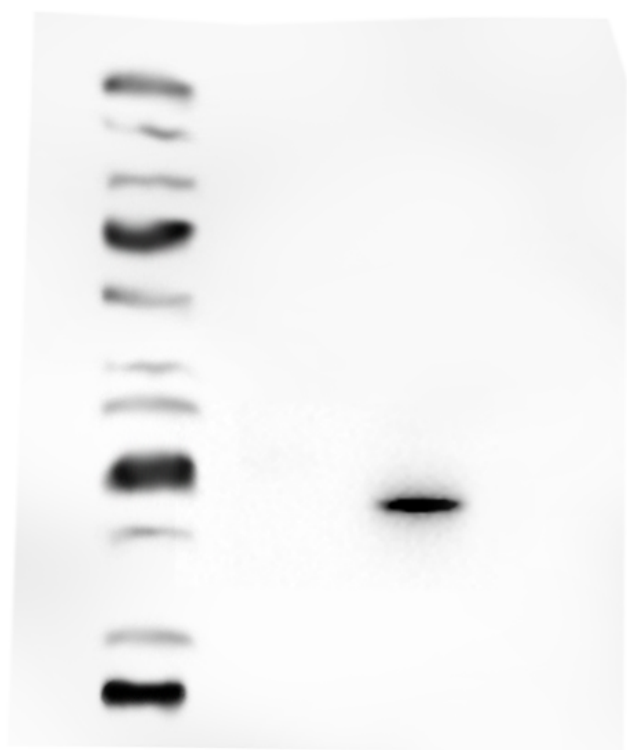

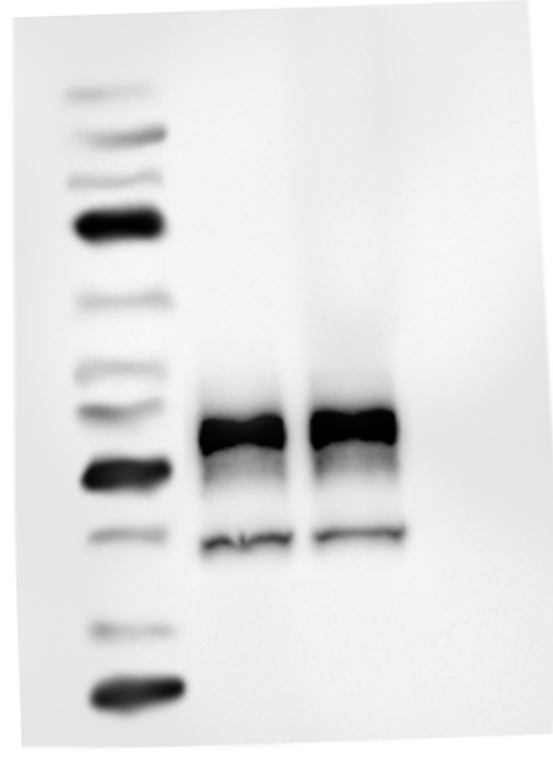

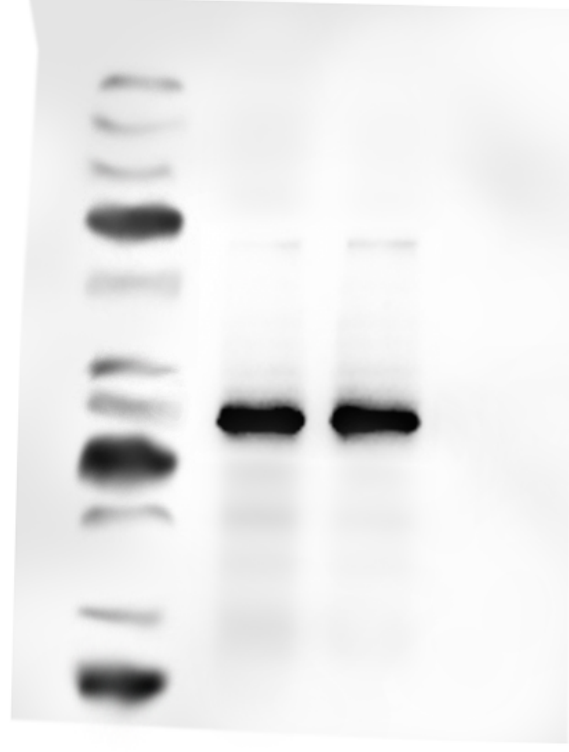

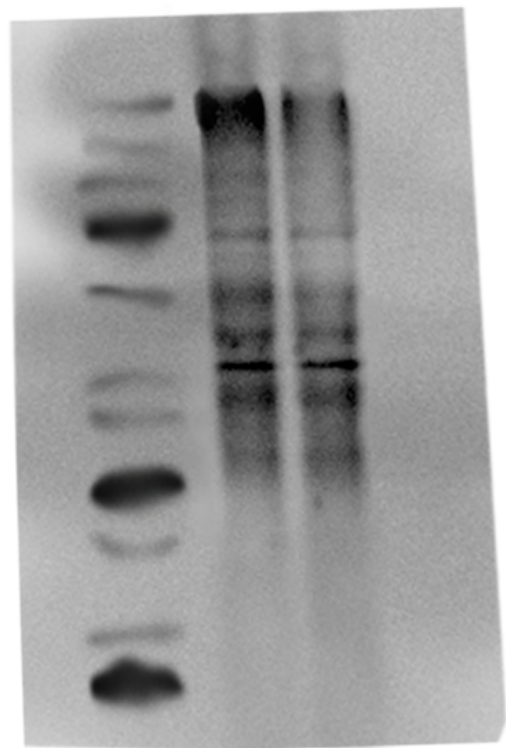

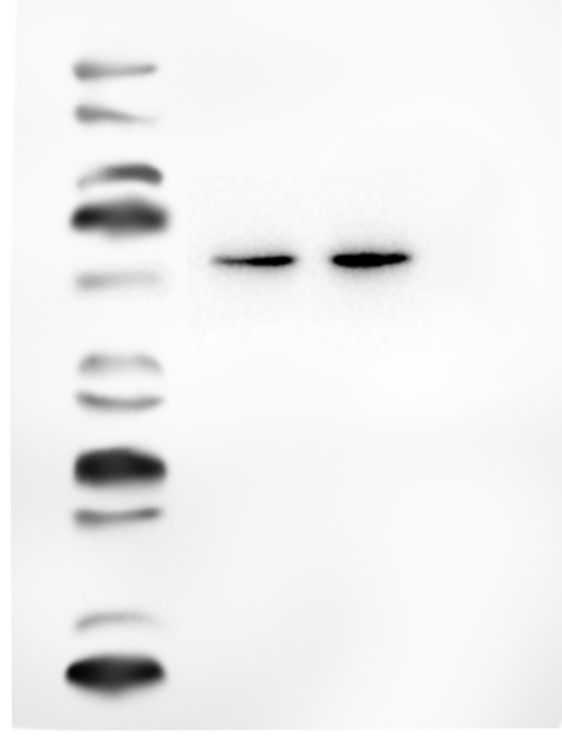

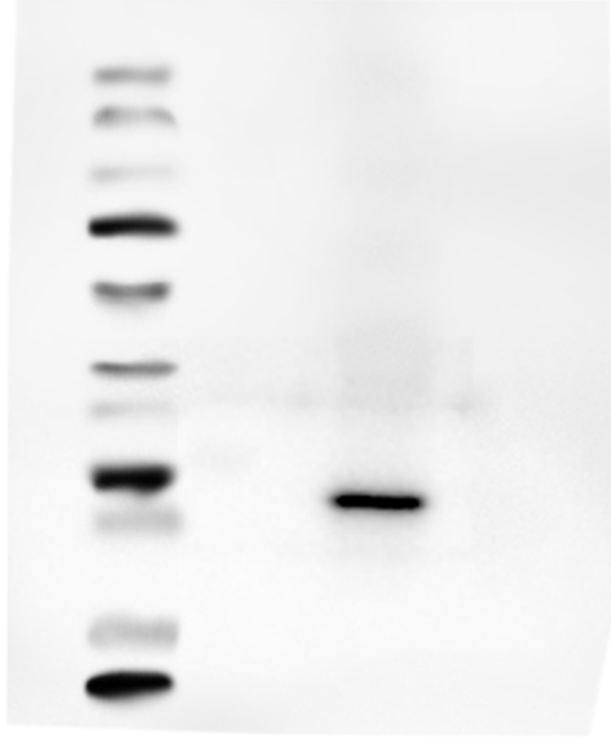

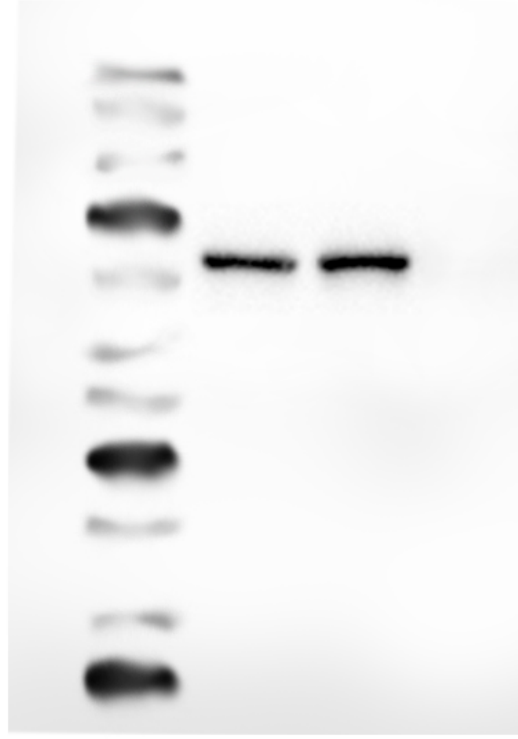

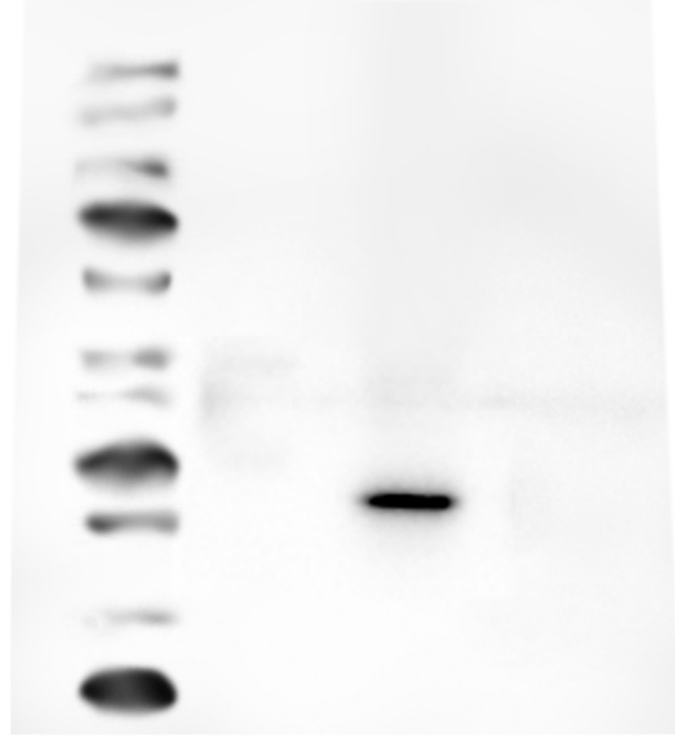

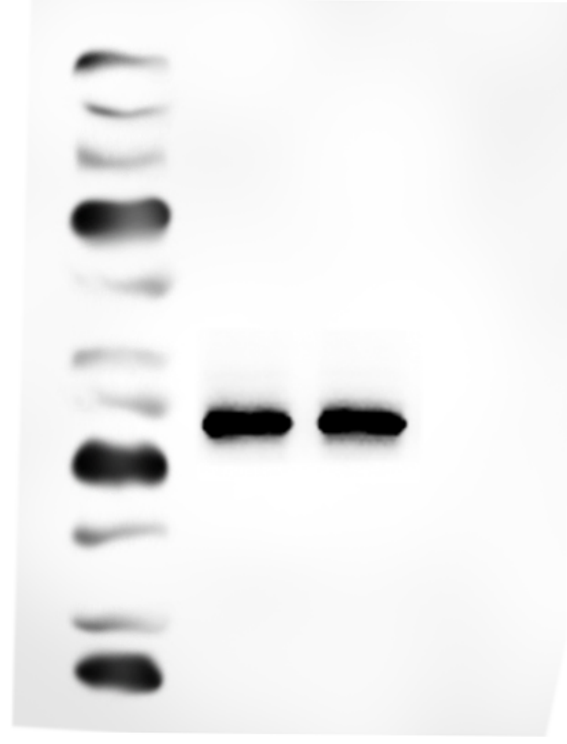

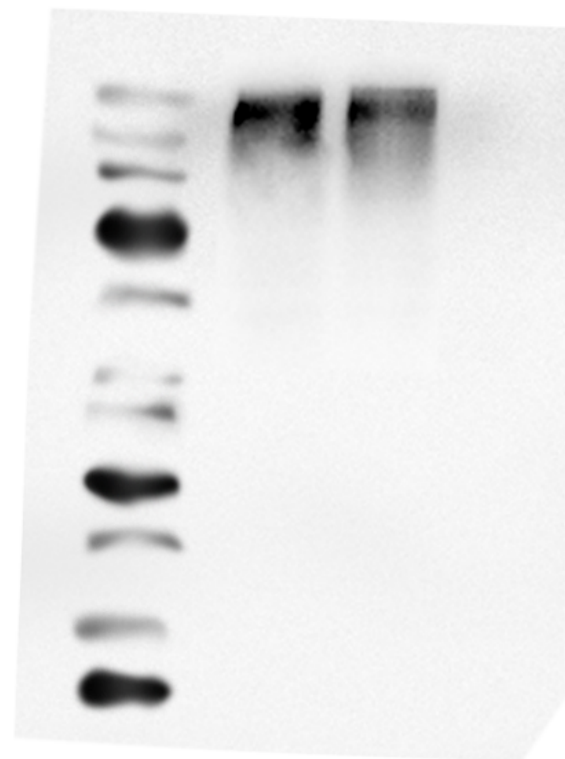

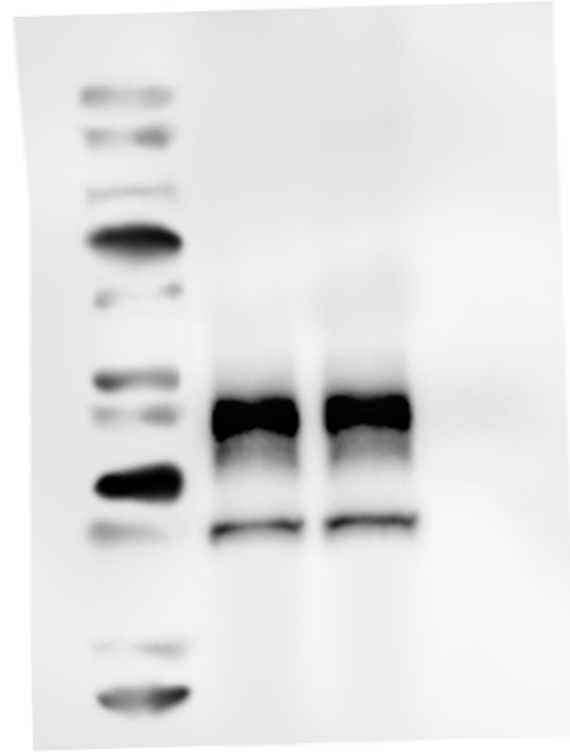

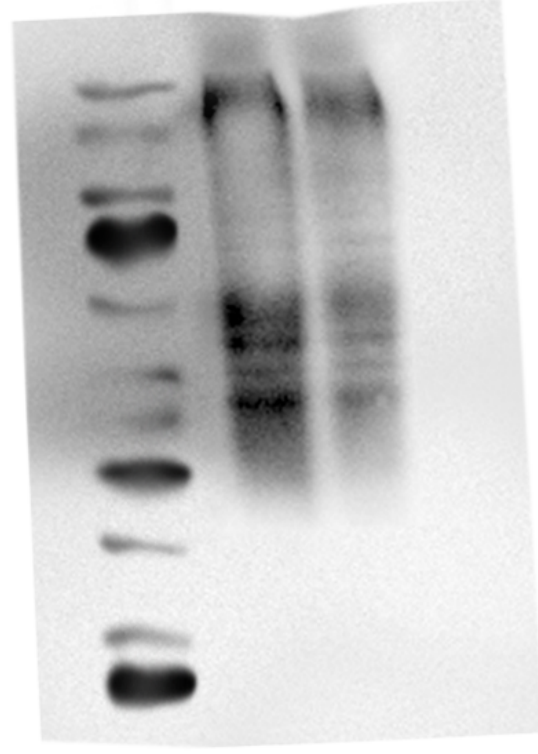

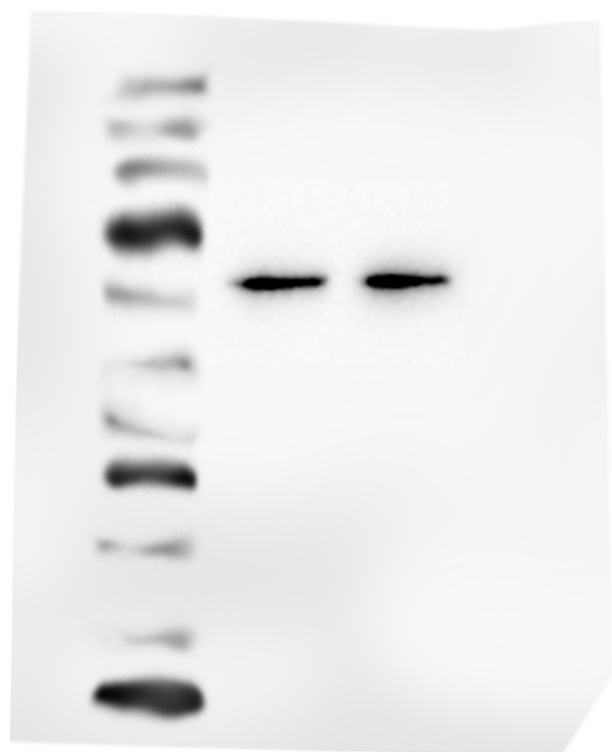

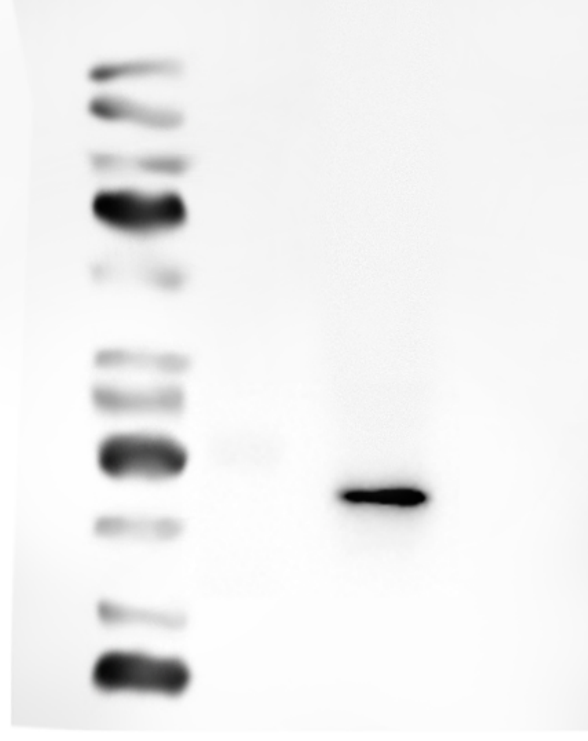

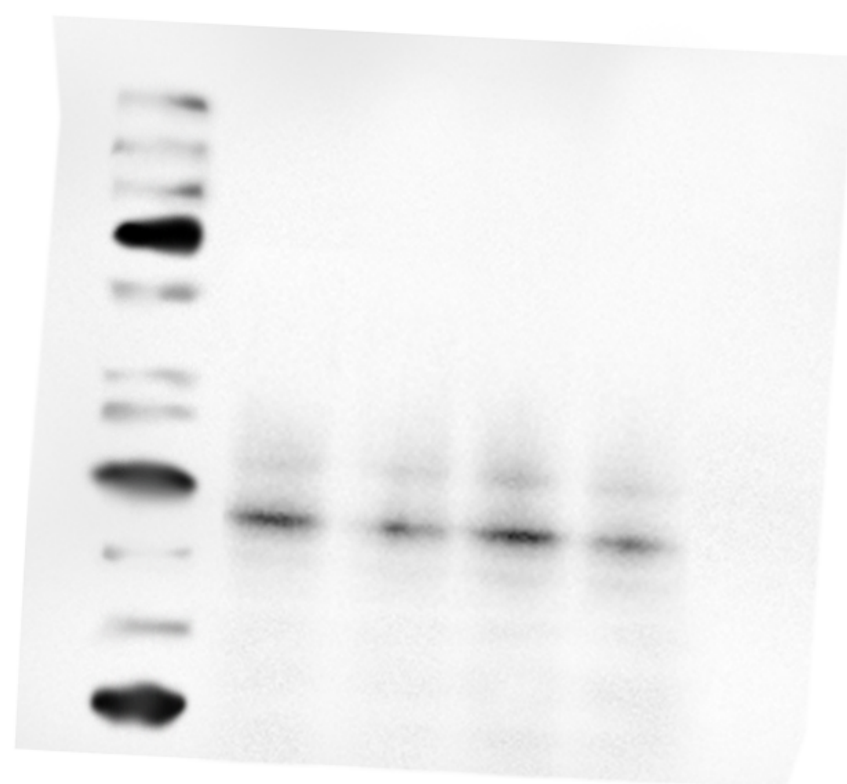

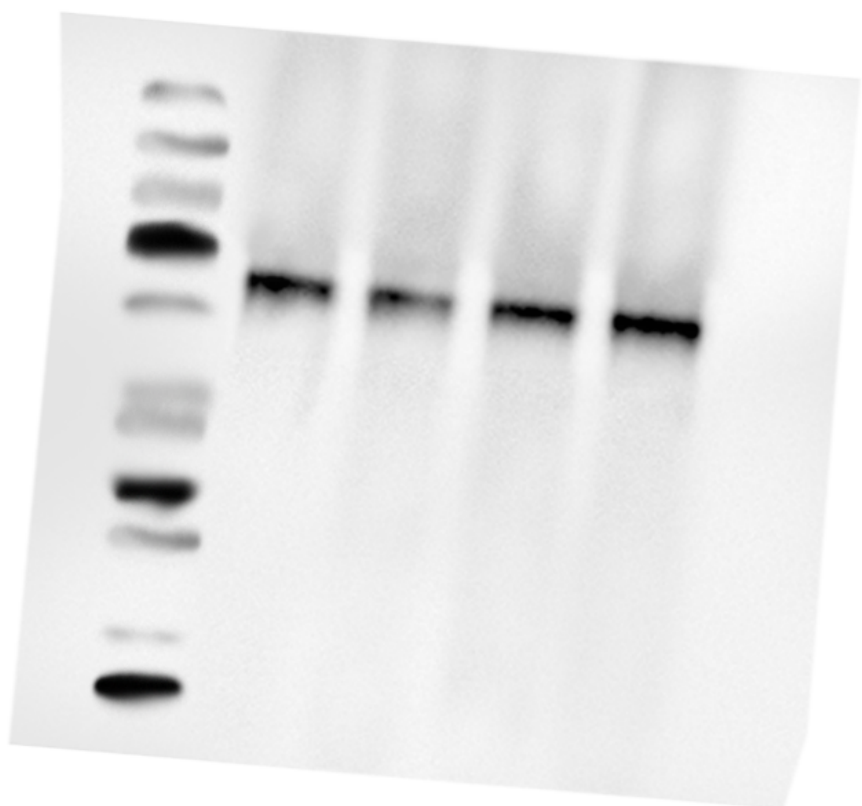

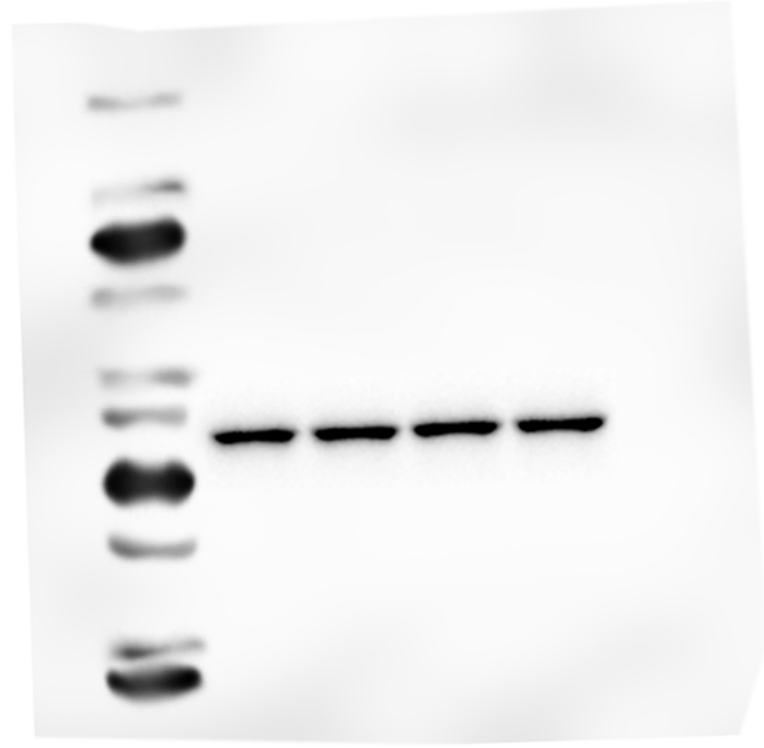

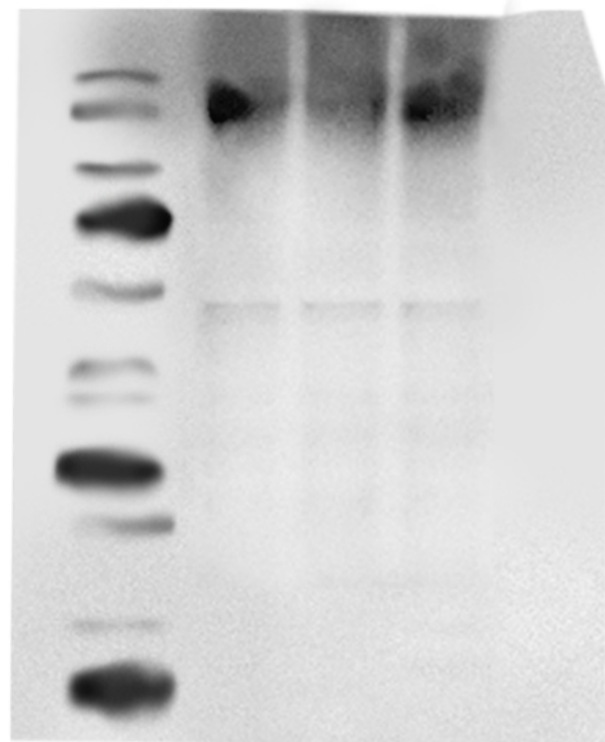

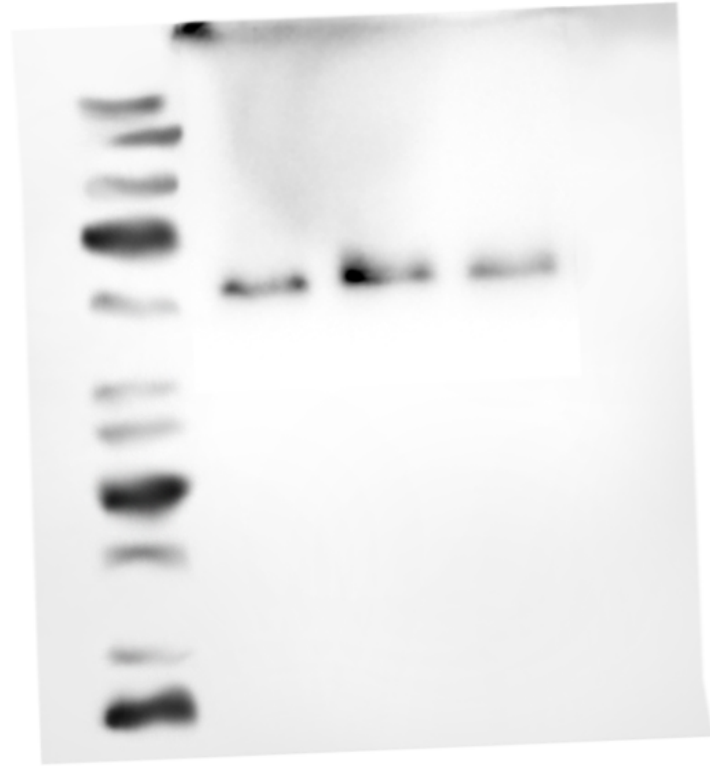

01 1000 1900

1 1

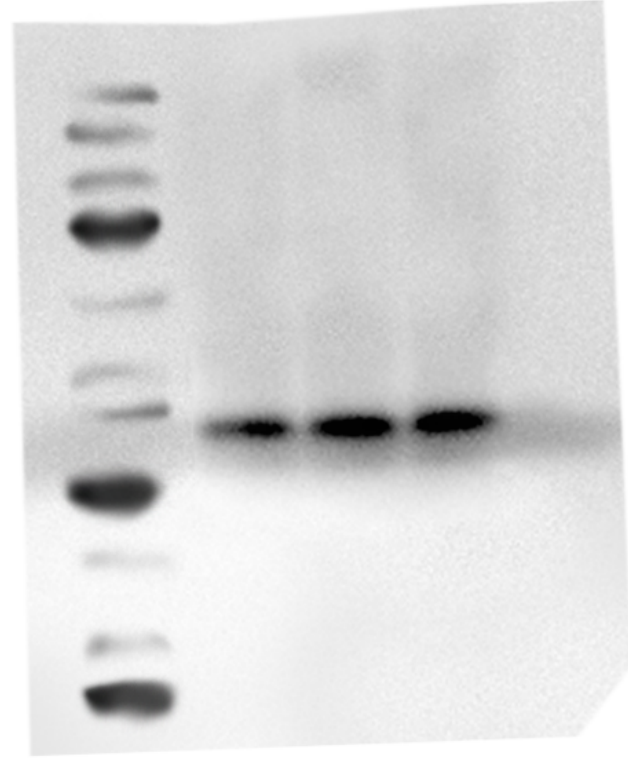

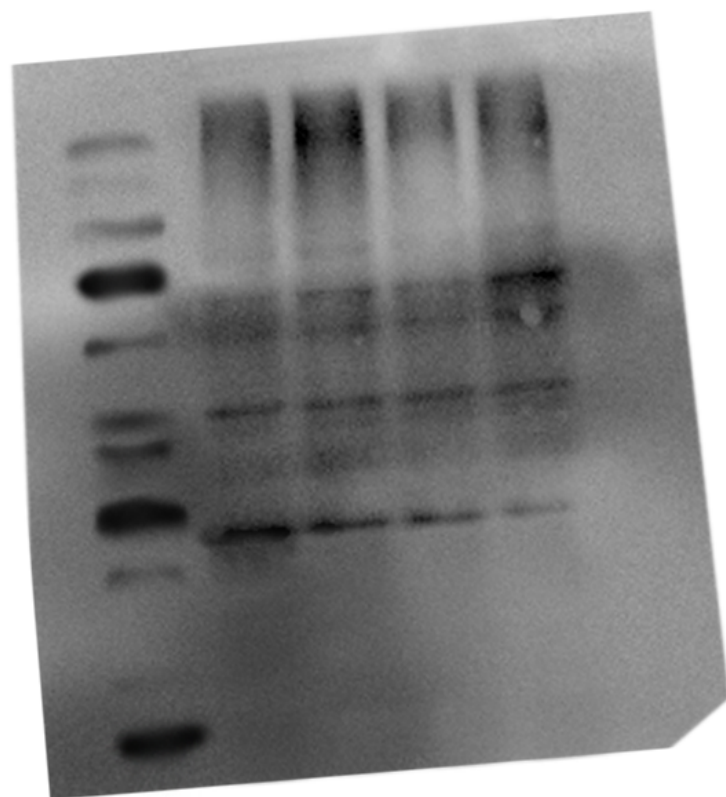

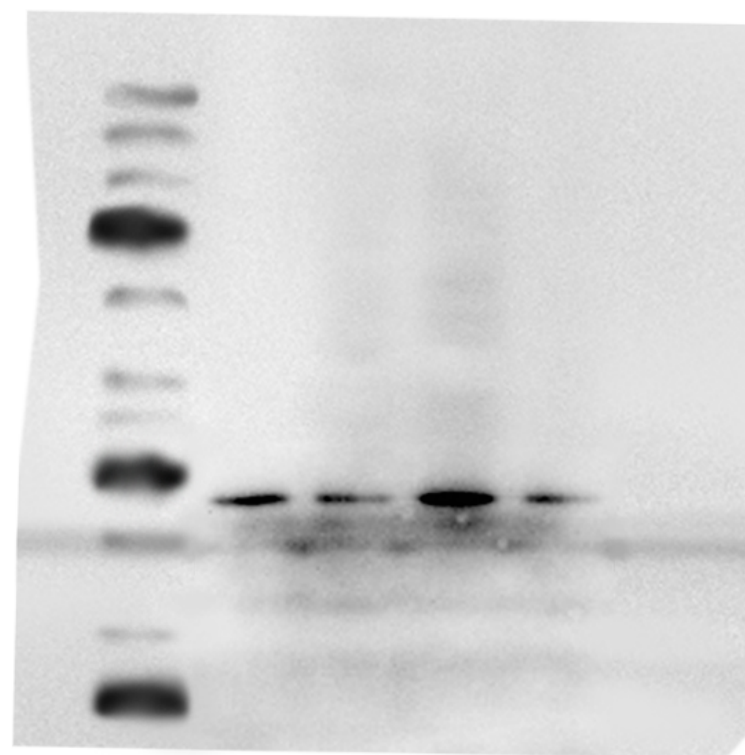

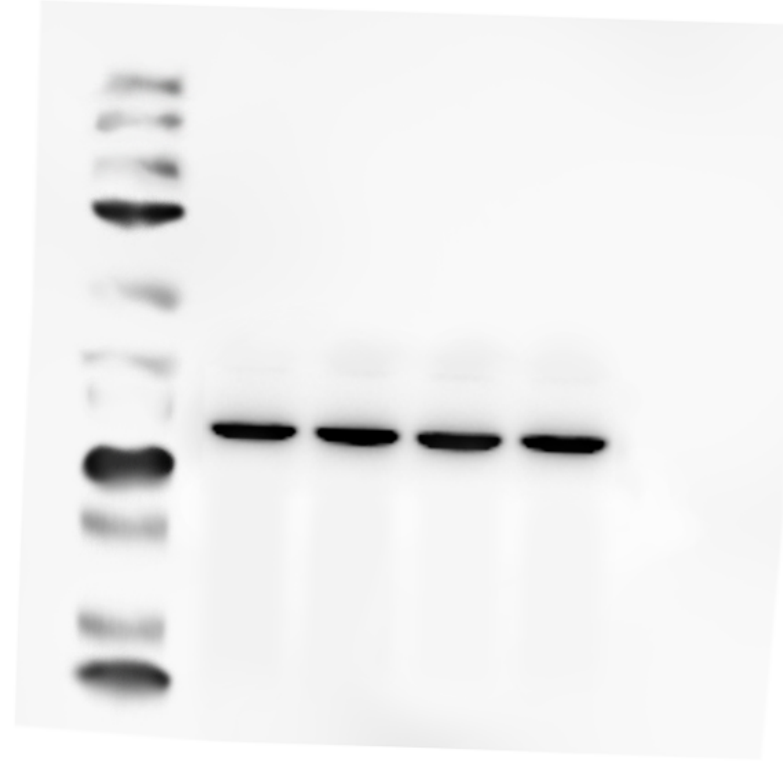

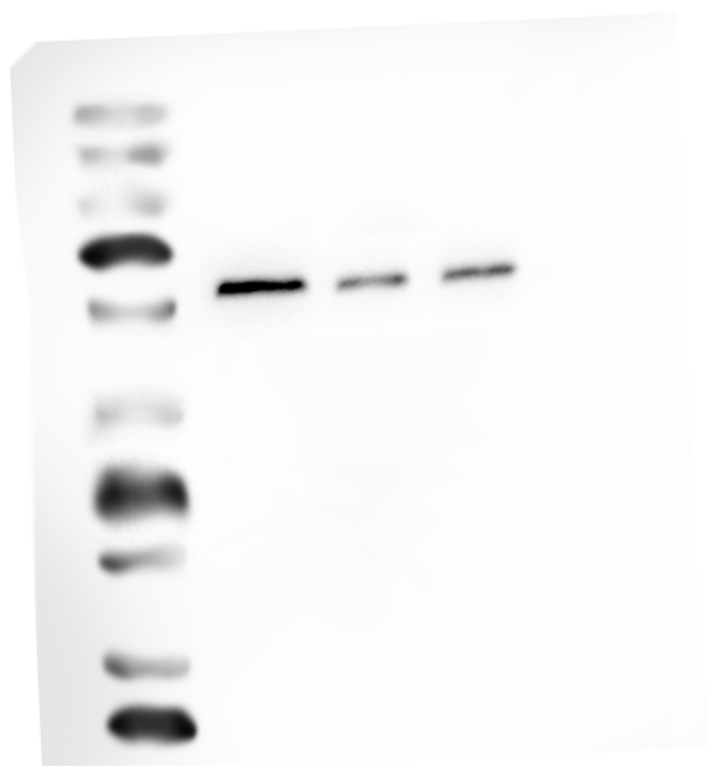

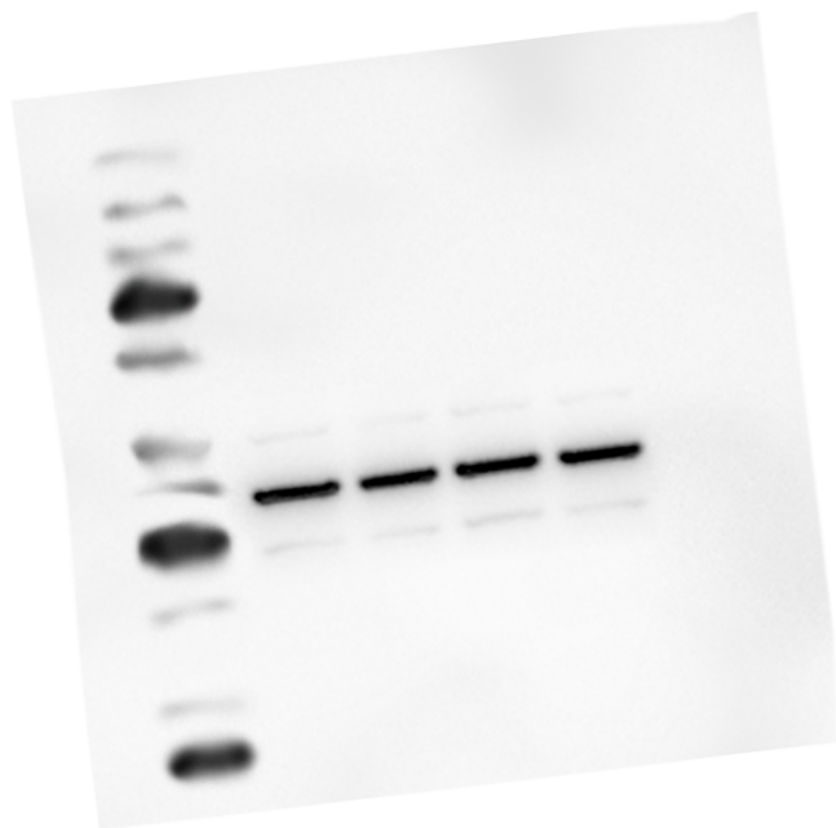

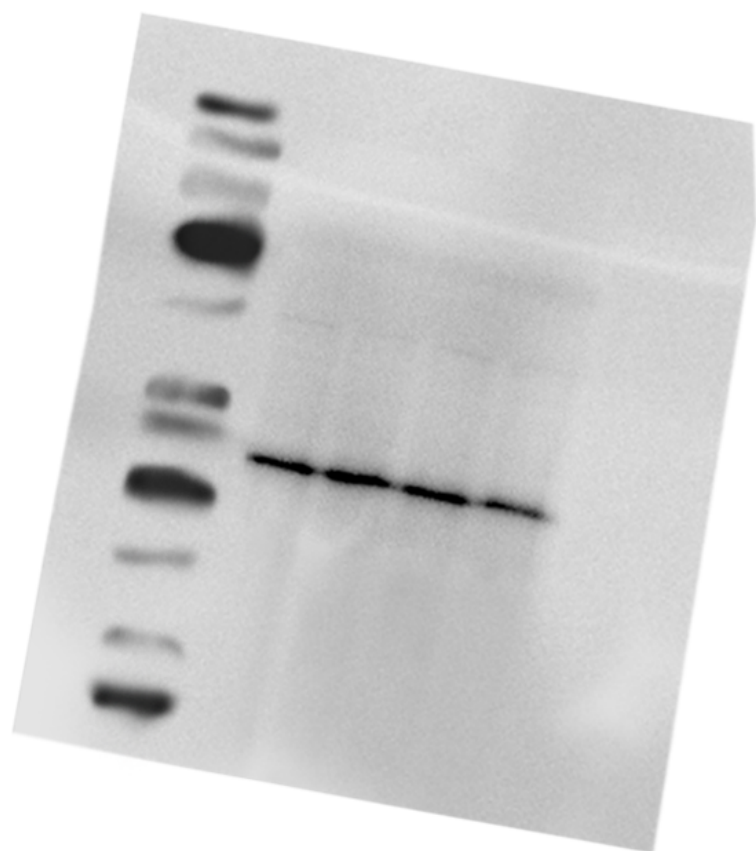

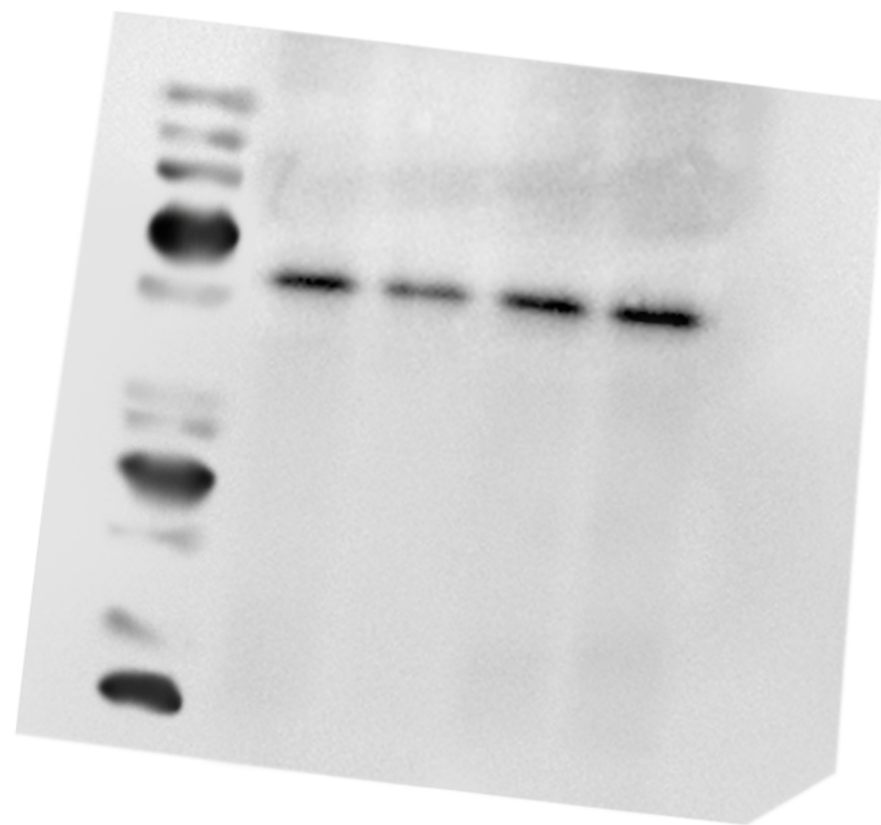

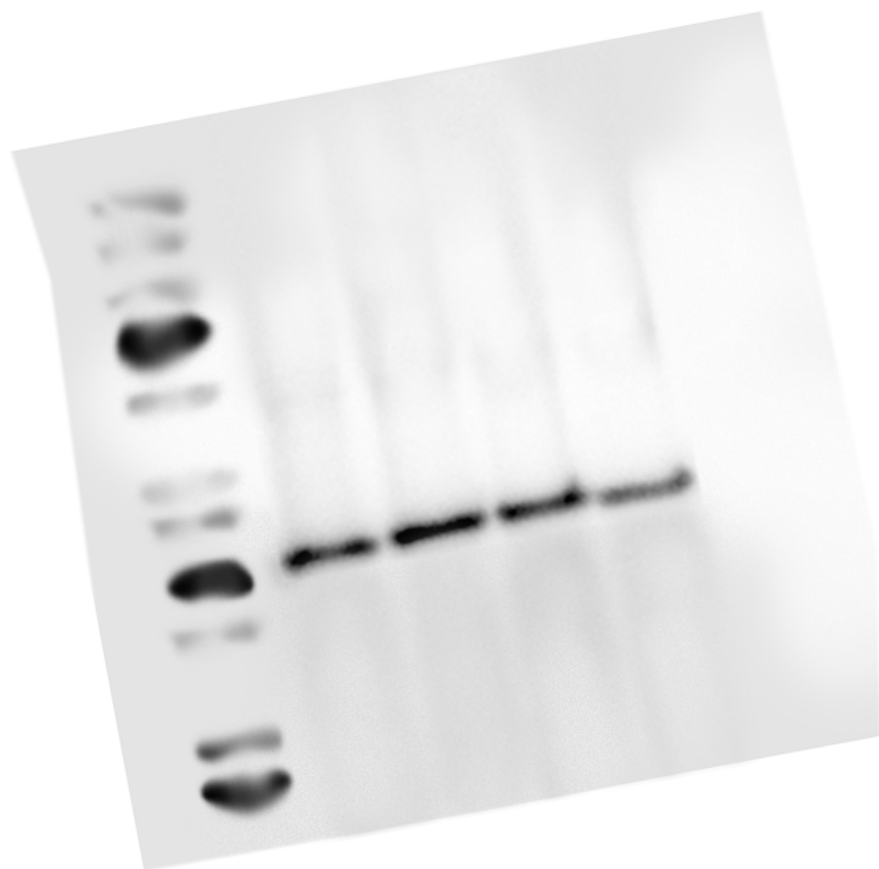

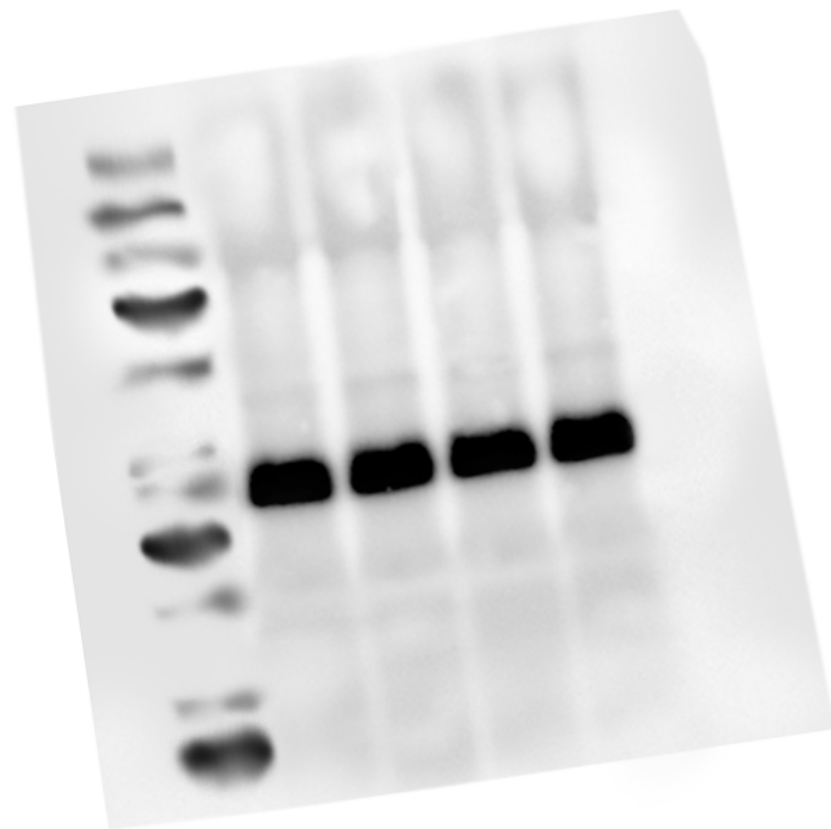

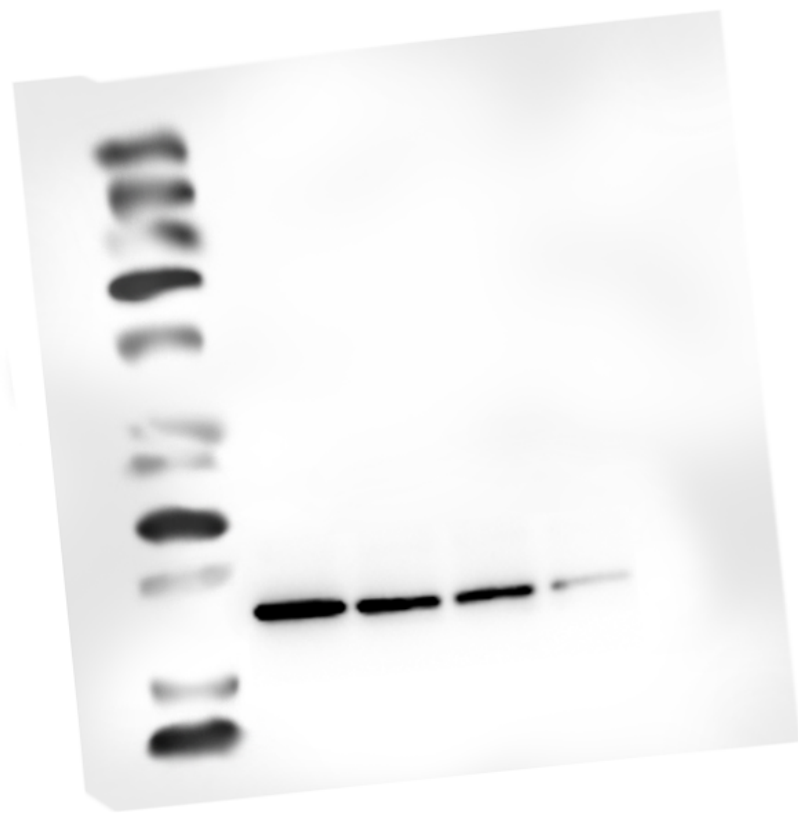

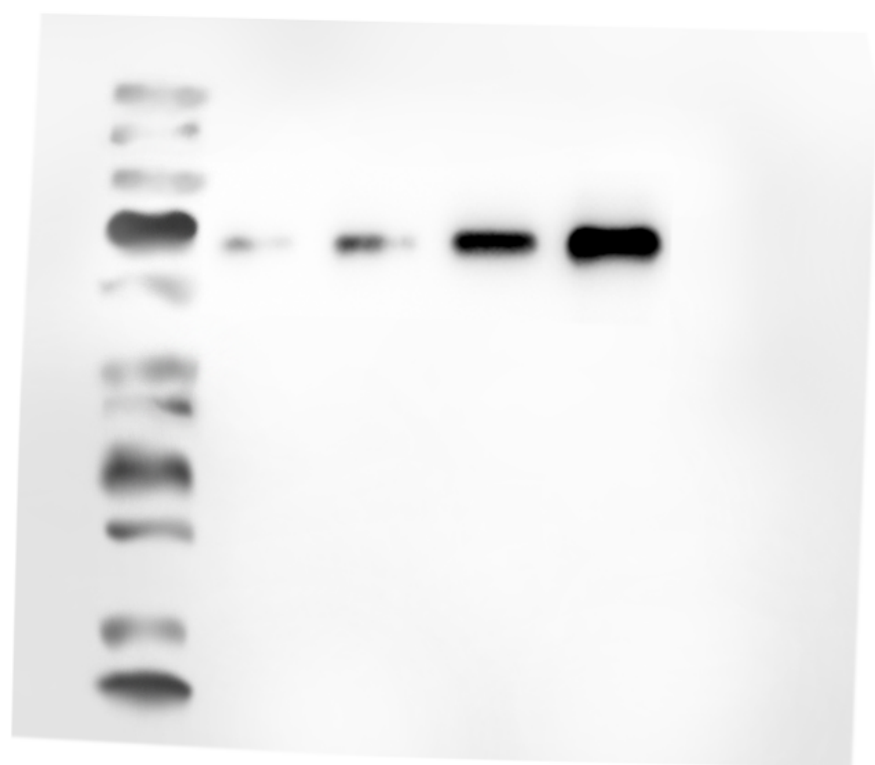

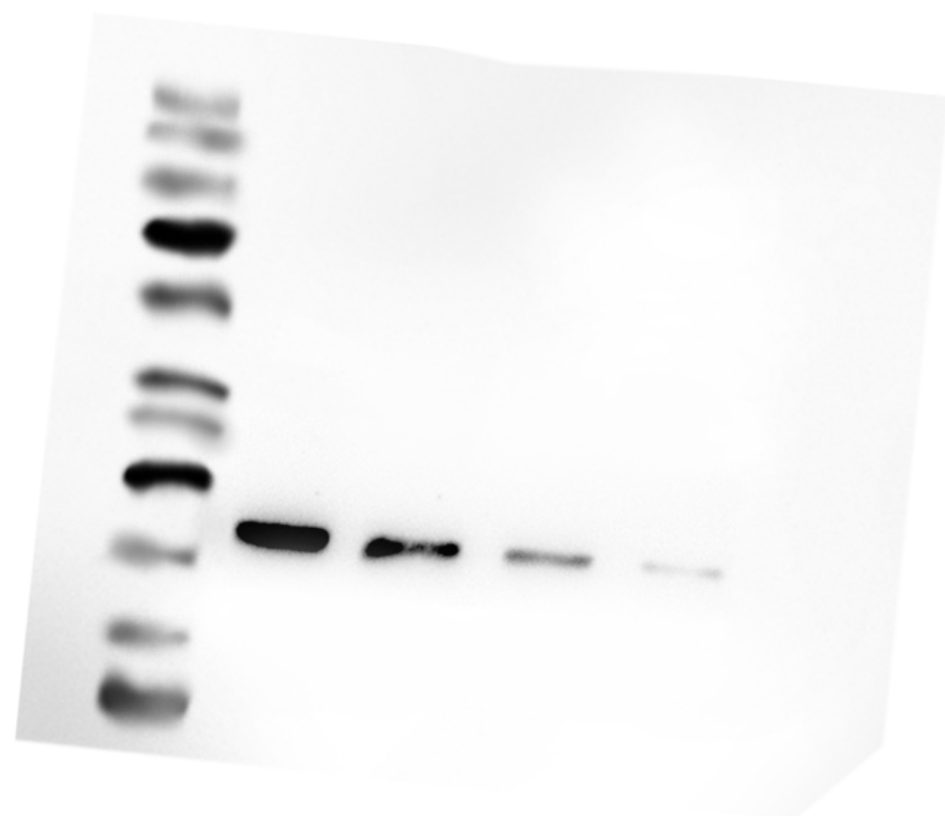

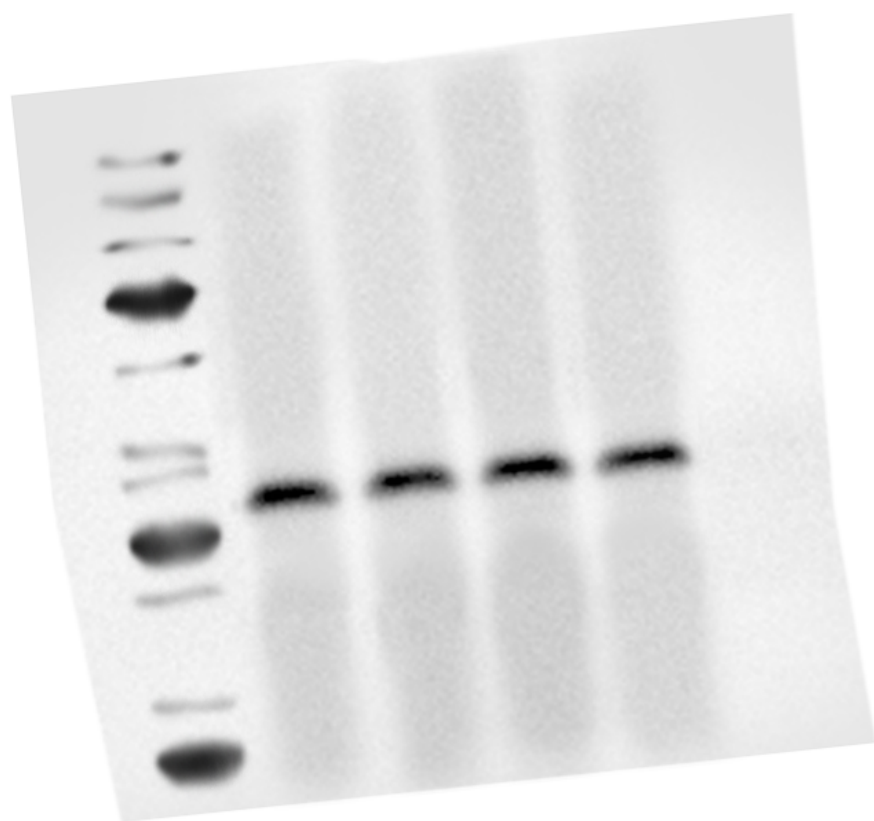

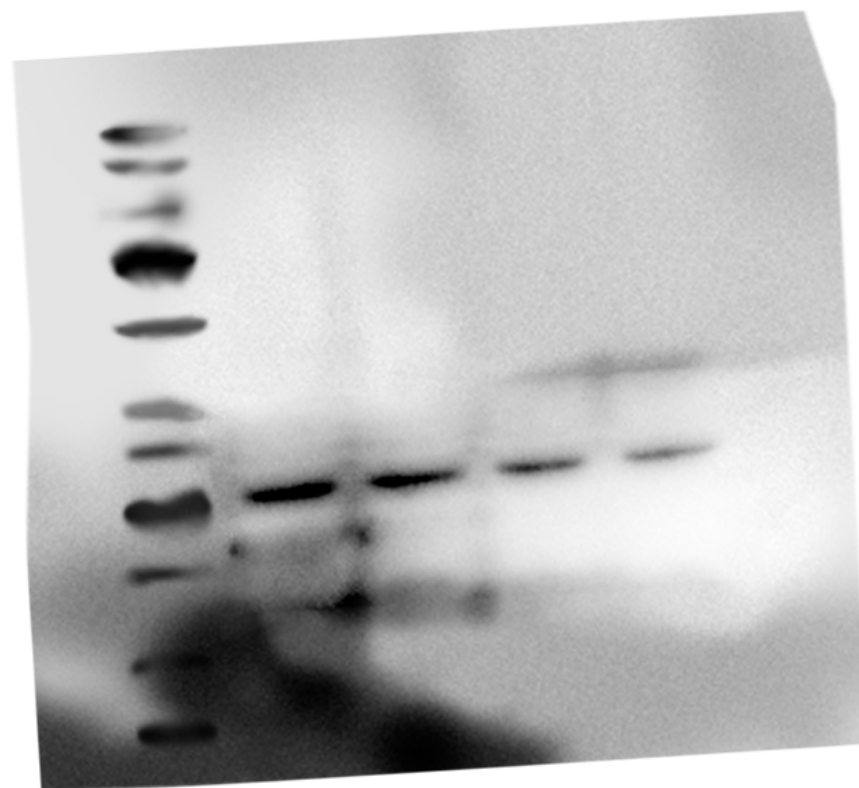

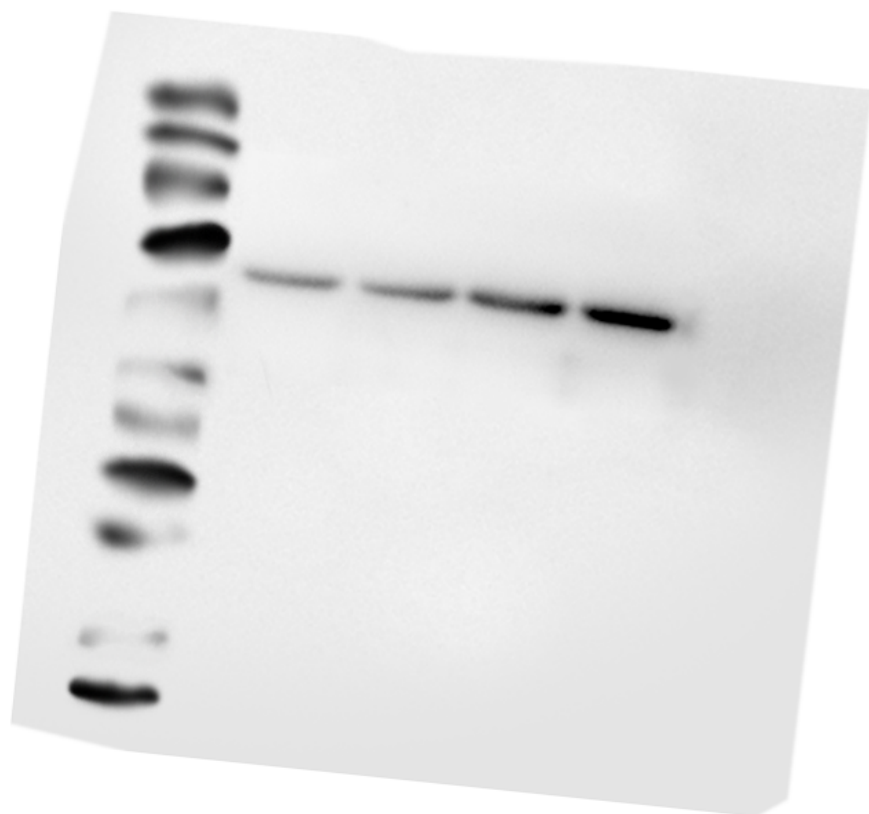

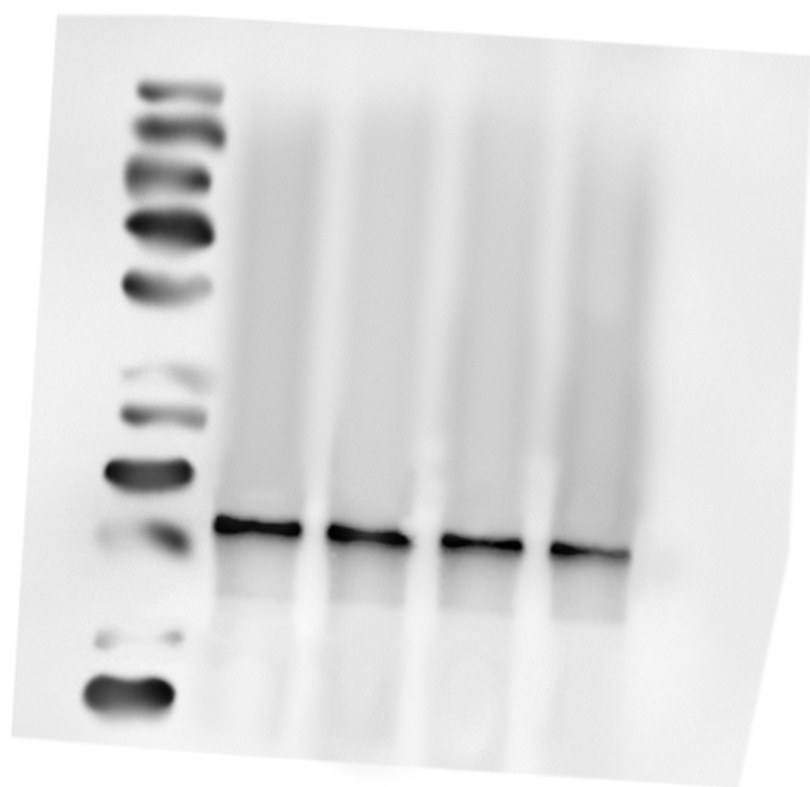

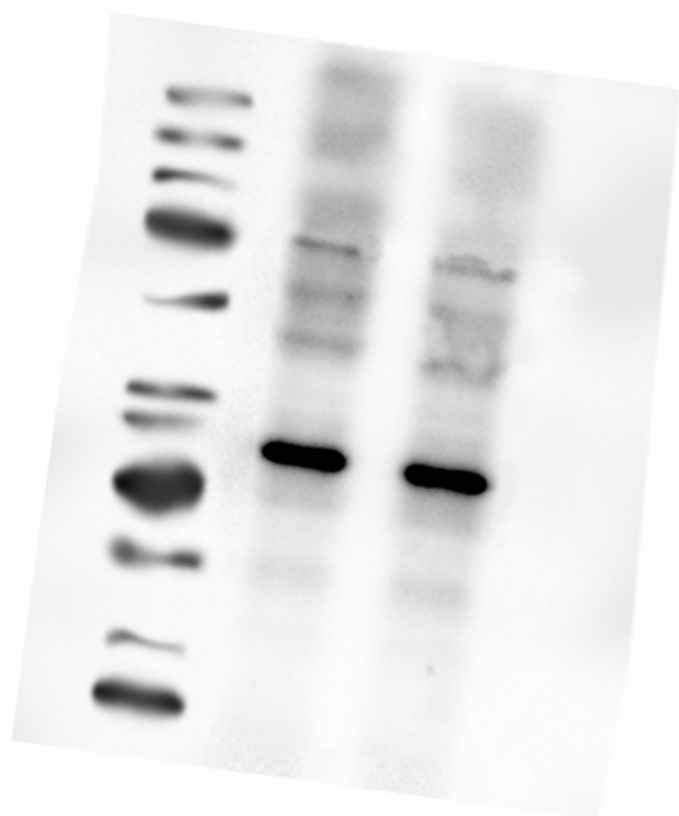

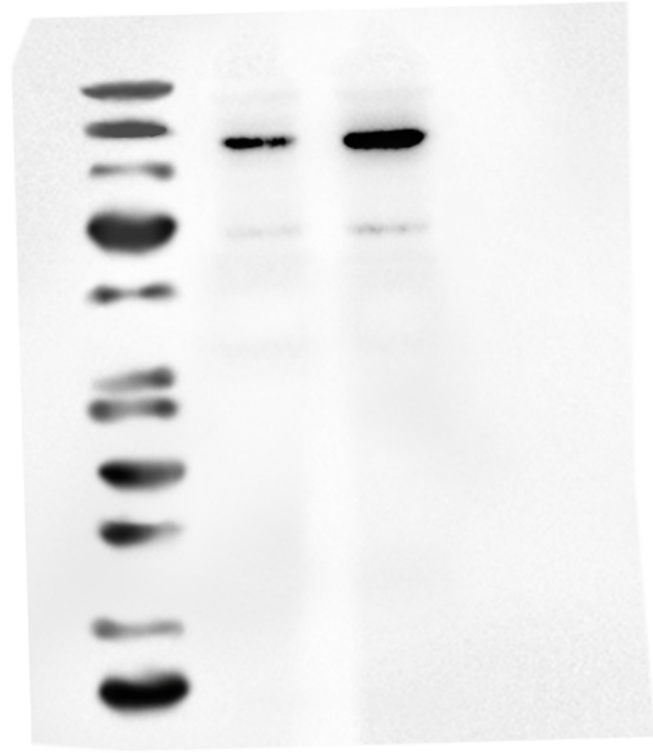

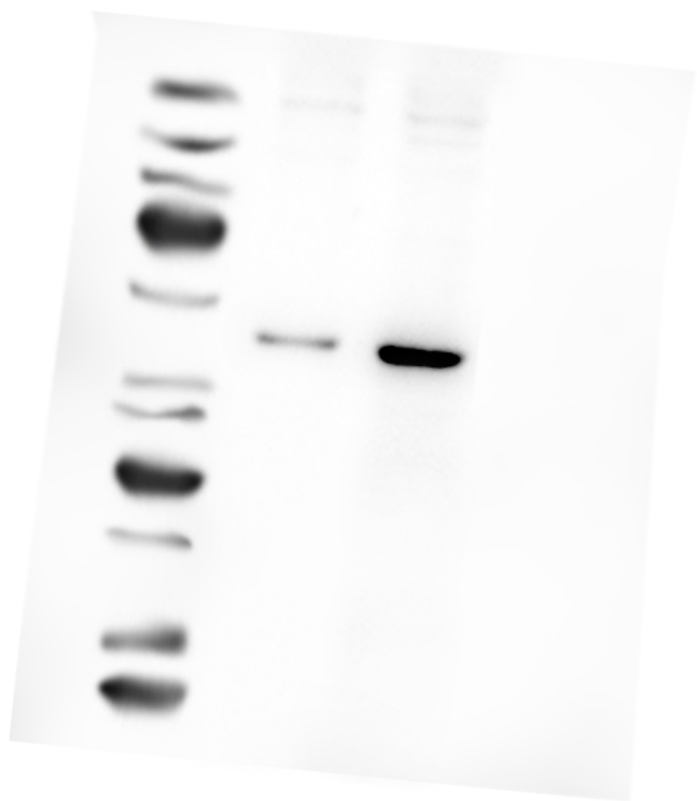

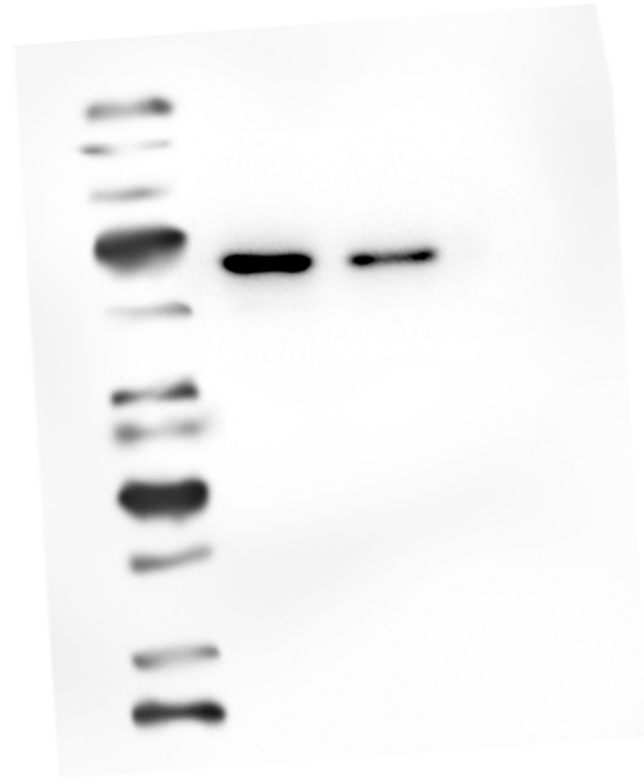

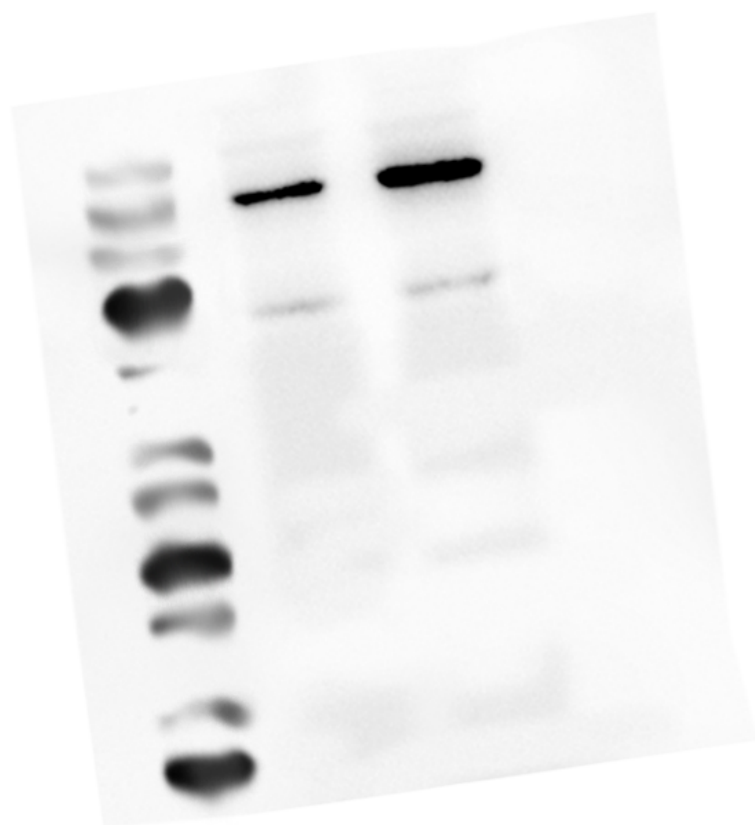

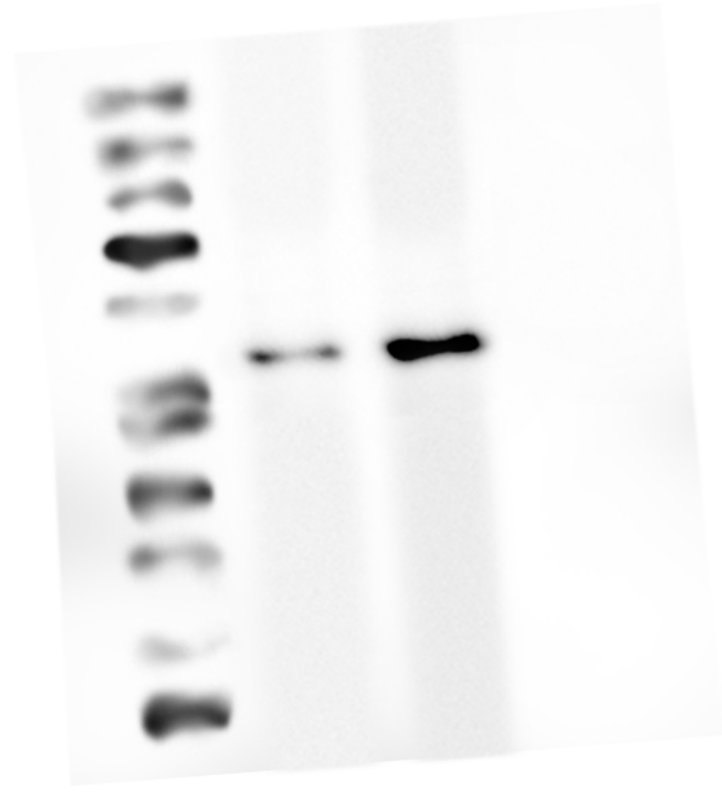

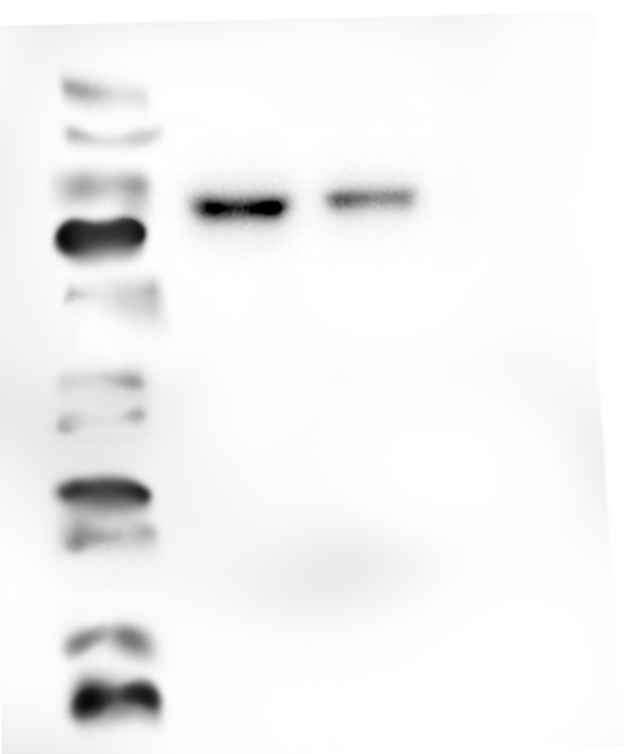

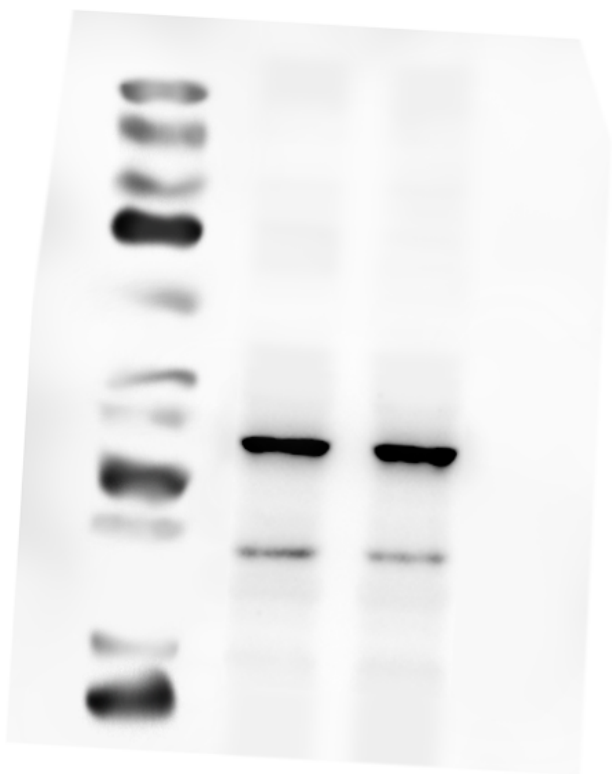

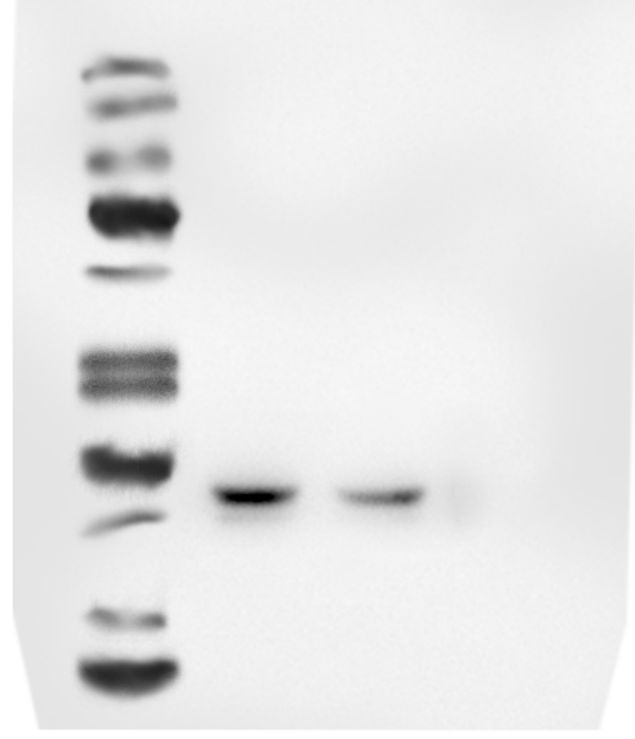

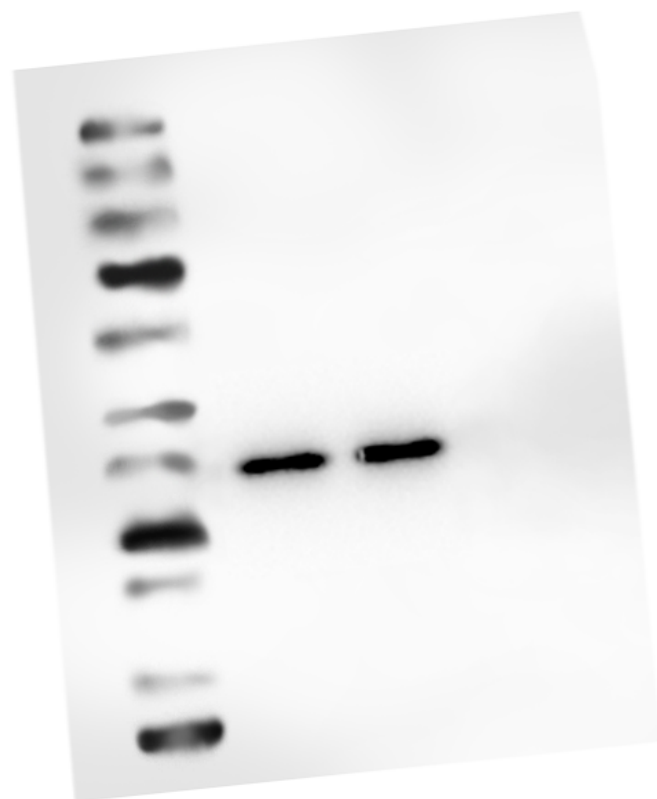

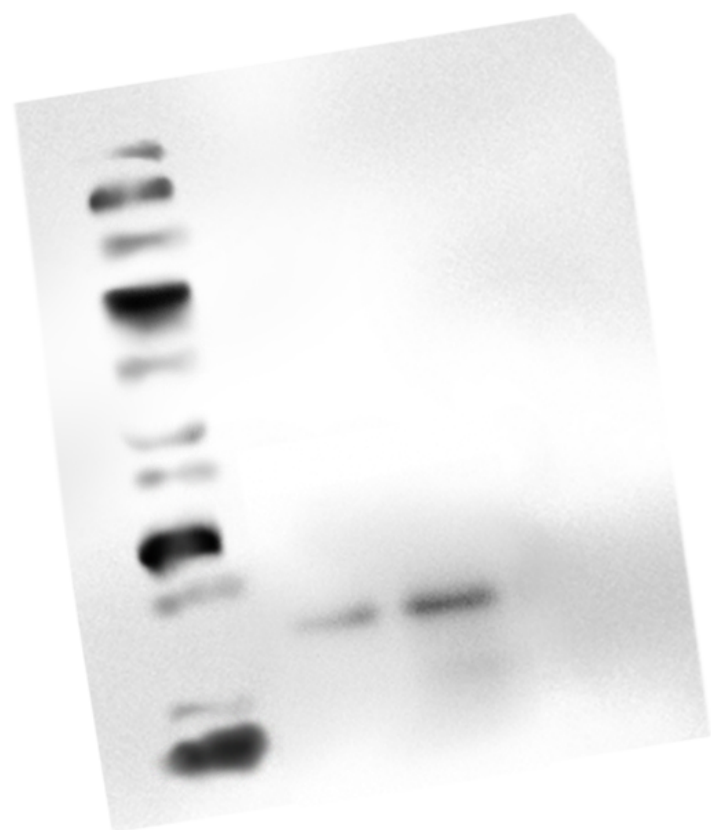

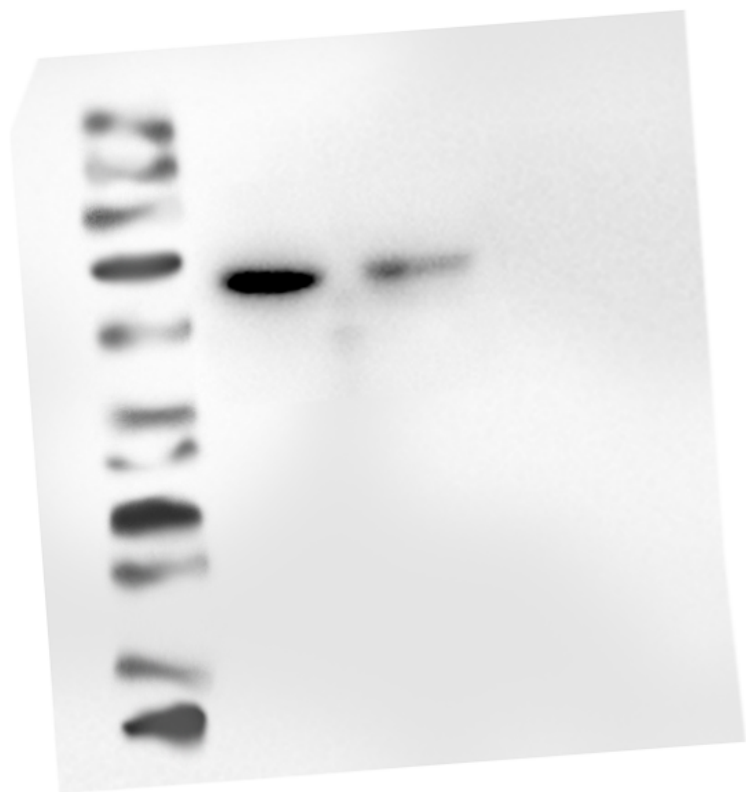

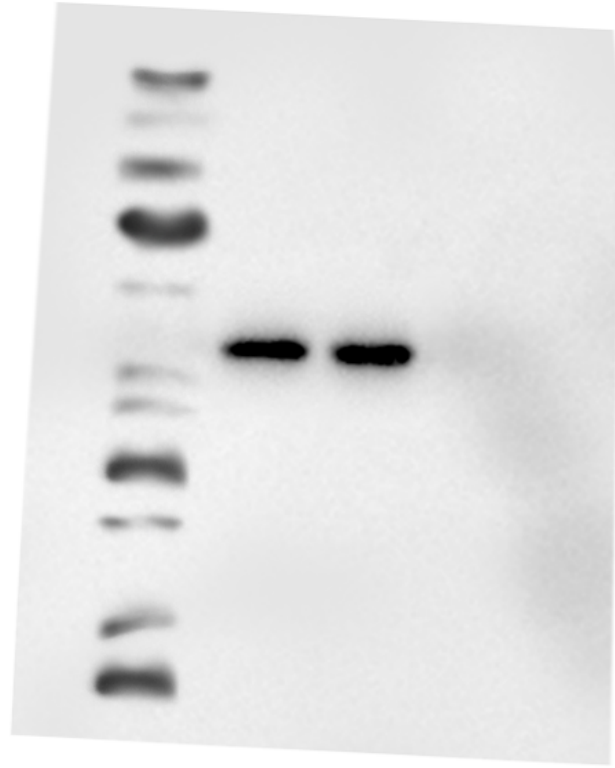

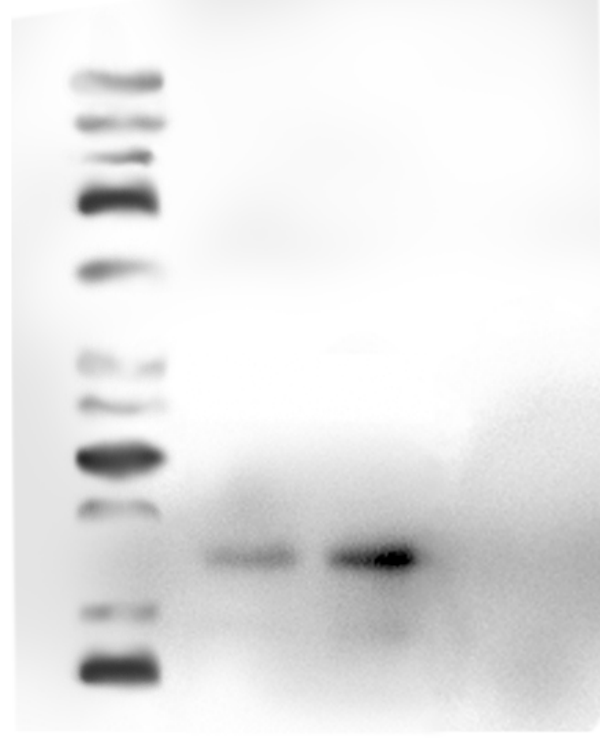

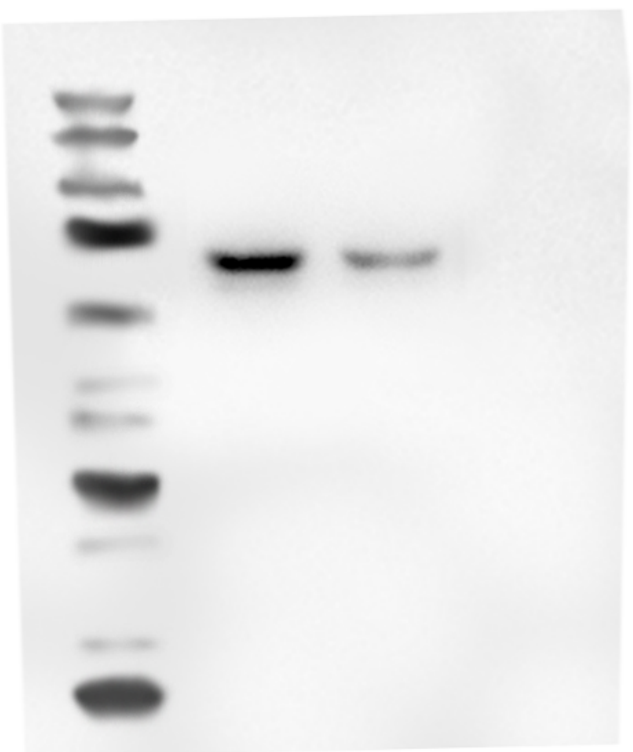

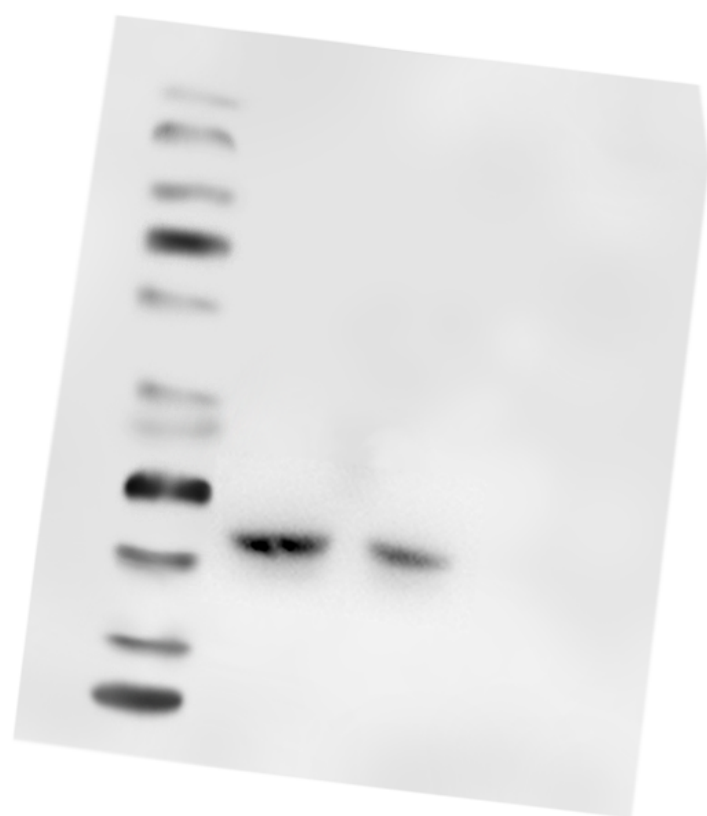

Supplement: Supplementary file 3 — Uncropped Blots [file 41419_2026_8415_MOESM3_ESM.pdf]

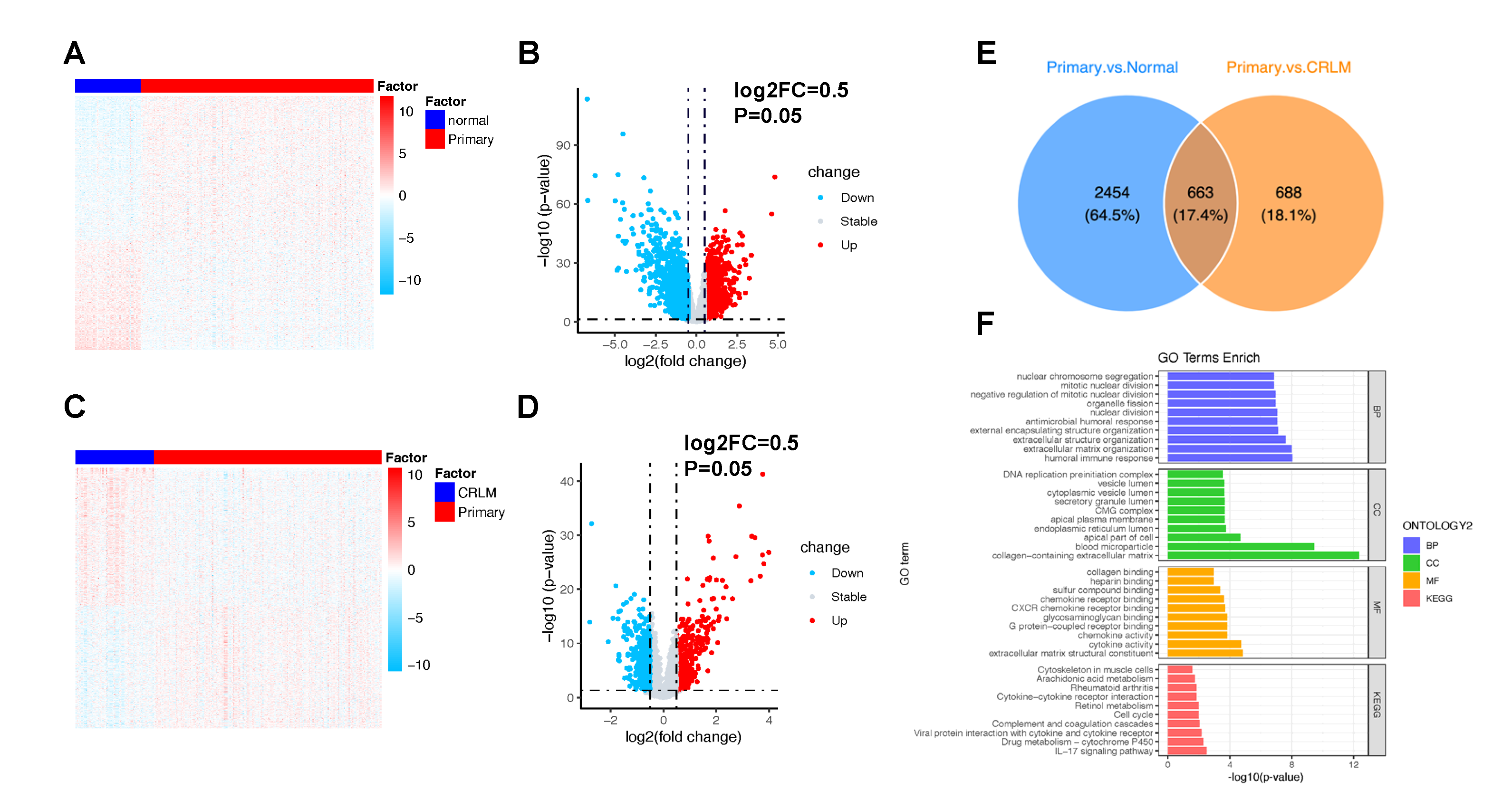

Supplement: Supplementary file 4 — Supplementary Figure S1 [file 41419_2026_8415_MOESM4_ESM.tif]

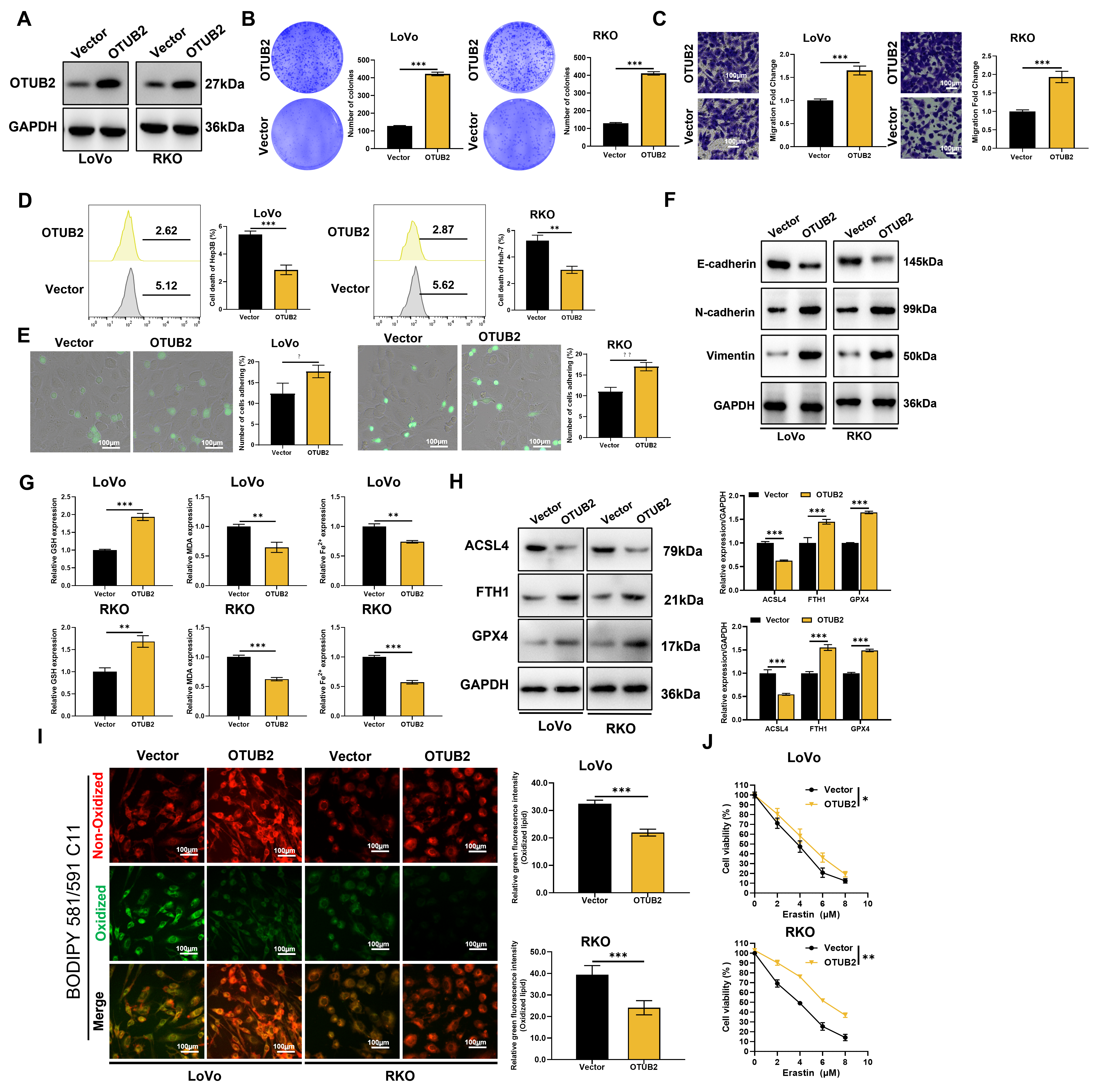

Supplement: Supplementary file 5 — Supplementary Figure S2 [file 41419_2026_8415_MOESM5_ESM.tif]
